# Identification of Novel sRNAs in Mycobacterial Species

**DOI:** 10.1371/journal.pone.0079411

**Published:** 2013-11-14

**Authors:** Chen-Hsun Tsai, Catherine Baranowski, Jonathan Livny, Kathleen A. McDonough, Joseph T. Wade, Lydia M. Contreras

**Affiliations:** 1 McKetta Department of Chemical Engineering, University of Texas at Austin, Austin, Texas, United States of America; 2 Wadsworth Center, New York State Department of Health, Albany, New York, United States of America; 3 Channing Laboratory, Brigham and Women’s Hospital, Harvard Medical School, Boston, Massachusetts, United States of America; 4 Department of Biomedical Sciences, University at Albany, SUNY, Albany, New York, United States of America; Glaxo Smith Kline, Denmark

## Abstract

Bacterial small RNAs (sRNAs) are short transcripts that typically do not encode proteins and often act as regulators of gene expression through a variety of mechanisms. Regulatory sRNAs have been identified in many species, including *Mycobacterium tuberculosis*, the causative agent of tuberculosis. Here, we use a computational algorithm to predict sRNA candidates in the mycobacterial species *M. smegmatis* and *M. bovis* BCG and confirmed the expression of many sRNAs using Northern blotting. Thus, we have identified 17 and 23 novel sRNAs in *M. smegmatis* and *M. bovis* BCG, respectively. We have also applied a high-throughput technique (Deep-RACE) to map the 5′ and 3′ ends of many of these sRNAs and identified potential regulators of sRNAs by analysis of existing ChIP-seq datasets. The sRNAs identified in this work likely contribute to the unique biology of mycobacteria.

## Introduction

The genus *Mycobacterium* contains many clinically relevant pathogens, including *Mycobacterium tuberculosis* and *Mycobacterium leprae*, the etiologic agents of tuberculosis (TB) and leprosy, respectively. *M. tuberculosis* alone was responsible for 8.7 million incident cases and 1.4 million deaths globally in 2011 [1]. The treatment of TB has become increasingly difficult due to its high drug resistance and adaptability; hence, the development of new and more effective treatments for TB is imperative.

Bacterial “small RNAs” (sRNAs) are small (50–400 nt), typically untranslated transcripts. Many sRNAs play important roles in gene regulation in response to environmental changes [2]. sRNAs can originate from their own independent genes or through the processing of larger transcripts [3]. To exert their function, sRNAs typically base-pair with target messenger RNAs (mRNAs), resulting in altered transcription, mRNA stability, or translation [4]. sRNAs are key regulators of pathogenesis in many bacterial species [5]. Recently, RNA-seq has been widely applied to identify novel sRNA candidates in many bacterial species [6]–[10], including *M. tuberculosis* [11]. sRNAs have also been identified/predicted in *M. tuberculosis* using other experimental approaches [12]–[14] and computational analysis of DNA sequence [13], [15], [16]. In total, 63 sRNAs have been experimentally validated in *M. tuberculosis*. sRNAs have also been identified in other mycobacterial species: 34 and 15 sRNAs have been experimentally validated in *Mycobcaterium bovis* BCG and *Mycobacterium smegmatis*, respectively [13].

In a previous study, we used computational predictions from the SIPHT (sRNA Identification Protocol using High-throughput Technologies) [17] algorithm to identify 144 sRNA candidates in *M. bovis* BCG. We selected 34 conserved sRNA candidates which we experimentally confirmed by Northern blot [13]. In the current study, we expanded our search to include all the remaining SIPHT predictions for *M. bovis* BCG as well as all SIPHT predictions for *M. smegmatis* (these were not explored in the previous study). By combining SIPHT predictions with large-scale Northern blot validation, we have identified 23 and 17 novel sRNAs in *M. bovis* BCG and *M. smegmatis*, respectively. Thus, we have substantially increased the number of experimentally validated sRNAs in mycobacterial species. We also analyzed existing ChIP-seq datasets to identify possible regulators of sRNA expression, and used Deep-RACE, a technique that combines high throughput RNA-seq with Rapid Amplification of cDNA Reads (RACE) [18], to identify sRNA 5′ and 3′ ends. Lastly, it is worth noting that this work is one of the first efforts to better coordinate genome annotation; all sRNA candidates identified in this study have been renamed with a new nomenclature [19].

## Materials and Methods

### Strains and Plasmids

*M. bovis* BCG (Pasteur strain, Trudeau Institute), and *M. tuberculosis* H37Rv were grown in mycomedium (as previously reported, [13]). *M. bovis* BCG and *M. tuberculosis* cultures were grown for 7 days, with shaking, to late-log phase. Cultures of *M. smegmatis* MC^2^155 were grown shaking at 37°C, in trypticase soy media supplemented with 0.05% Tween 80 for 18 hours with shaking (late-log phase).

### Phylogenetic Selection of Computationally Predicted sRNA Candidates

Small RNA candidates of *M. smegmatis* were predicted using the SIPHT program with the same parameters as described previously [17], [20]. SIPHT identifies potential sRNA candidates based on the presence of intergenic sequence conservation upstream of putative Rho-independent terminators. SIPHT has been widely applied in sRNA studies [21]–[23], and its reliability has been tested and compared with other algorithms [24].

### RNA Isolation and Northern Blot Analysis

RNA was isolated as previously reported [13]. Northern blot analysis was performed as previously reported [13]; probes were designed according to SIPHT predicted sequences and tested in *M. bovis* BCG, *M. smegmatis* and *M. tuberculosis* [13]. All the oligonucleotides that were used in this study are listed in Table S1.

### ChIP-seq Analysis

We analyzed existing ChIP-seq datasets for 55 *M. tuberculosis* transcription factors extracted from a previous study [25]. ChIP-seq peak positions were compared to the 5′ end positions of *M. bovis* BCG and *M. tuberculosis* sRNAs from the current study and two previous studies [12], [15]. For *M. bovis* BCG sRNAs, we first identified the equivalent region of the *M. tuberculosis* H37Rv genome. Possible sRNA regulators were selected if the ChIP-seq peak was located within 100 bp upstream and 20 bp downstream of an sRNA 5′ end.

### Deep 5′ and 3′ RACE

Deep 5′ RACE and Deep 3′ RACE were performed as previously described [18] with the following exceptions. Deep 5′ RACE libraries and Deep 3′ RACE libraries were pooled and sequenced together using an Ion Torrent 316 chip (Wadsworth Center Applied Genomic Technologies Core Facility). For Deep 5′ RACE, sequence reads were identified by the presence of the expected adapter sequence at the read 5′ end. Adapter sequences were removed and reads of >40 nt were mapped to the reference genomes using BWA [26]. For Deep 3′ RACE, sequence reads were identified by the presence of the expected adapter sequence. Adapter sequences were removed. The oligo-dT stretch was removed by identifying the first consecutive pair of bases not including a “T” and removing all sequence upstream of this. Sequences of >40 nt were mapped to the reference genomes using BWA [26]. For both Deep 5′ RACE and Deep 3′ RACE, 5′ and 3′ ends were identified as the position with the most sequence reads, and with a minimum of 5 reads. Sequences of all primers used for Deep RACE are listed in Table S2.

## Results and Discussion

### Prediction of sRNAs in silico using SIPHT

**Figure 1:**
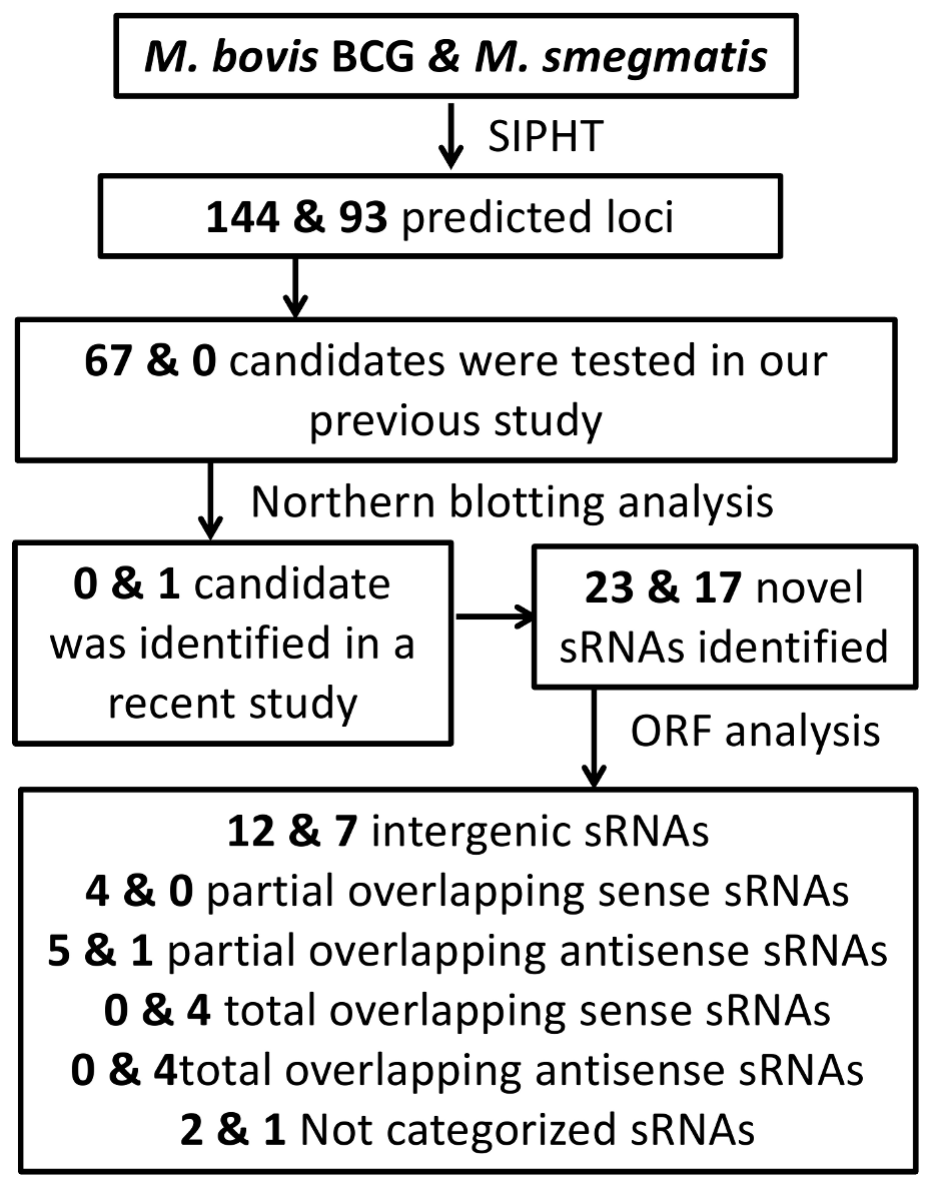
Schematic for sRNA identification. This schematic shows the combination of computational approaches and Northern blotting analysis used to identify the reported novel sRNAs in *M. bovis* BCG and *M. smegmatis*.

Using SIPHT, we identified 93 candidate sRNAs in *M. smegmatis* (refseq: NC_008596) (Table S3) and 144 candidate sRNAs in *M. bovis* BCG (refseq: NC_008769) (Table S4). Tables S3 and S4 include a detailed description of the predicted coordinates, orientations, sizes and neighboring upstream and downstream genes. Northern probes were designed according to SIPHT prediction. Figure 1 summarizes the overall approach that was employed in this work for sRNA identification and confirmation.

### 17 Novel sRNAs Identified in Mycobacterium Smegmatis

**Figure 2:**
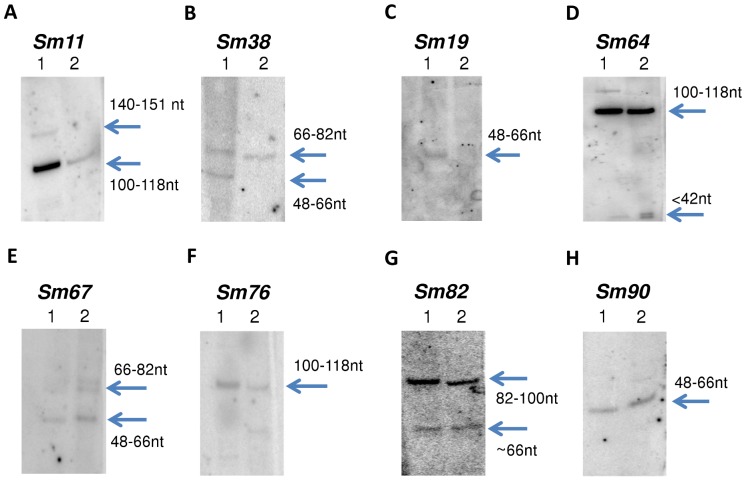
Northern blotting confirmation of sRNA candidates in *M.* smegmatis. Selected images of Northern blotting analysis for validated *M. smegmatis* sRNAs; the remaining images are included in Figure S1. Lane 1 and 2 indicate total RNA samples extracted from *M. smegmatis* and *M. bovis* BCG, respectively. We used Phi-X174/Hae III Marker for the size prediction. The probes we used for this analysis are listed in Table S1.

All 93 *M. smegmatis* sRNA candidates were tested by Northern blot using oligonucleotides in both orientations; expression was confirmed for 18 sRNA (listed in Table 1; see blot pictures in Figure 2 and Figure S1). One of them (Sm32/33) was identified in recent work as IGR-1 with similar size, coordinates and same orientation [27]. Thus, 17 *M. smegmatis* sRNAs identified here have not been experimentally demonstrated in any previous studies. In our previous study [13], we reported homologs of 6 *M. smegmatis* sRNA candidates (Sm32/Sm33, Sm35, Sm46, Sm47, and Sm74) in *M. bovis* BCG (Mpr13/Mcr14, Mpr20, Mpr3, Mpr4, and Mpr5, respectively). These were confirmed directly in *M. smegmatis* by Northern blotting in current study and listed in the 17 novel confirmed sRNAs. A homologue of Sm76 was previously identified in *M. tuberculosis* by RNA-seq [15] and microarray analysis [14] but not otherwise experimentally confirmed. All of the validated sRNAs were in the same orientation to that predicted by SIPHT. This suggests that the sequence specificity of SIPHT for this prediction is higher than in our previous work, in which 9 out of 37 of the validated sRNAs were in the opposite orientation to the prediction [13]. All confirmed sRNAs were assigned gene names according to a recently-proposed nomenclature [19].

**Table 1:** sRNAs confirmed by Northern blotting analysis in *M. smegmatis.*

			Homology confirmed by Northern analysis in:			
	5′ end*	3′ end*	*M. bovis* BCG	*M. tuberculosis*	New nomenclature	Length Confirm by Northern blotting analysis(nt)
**Intergenic sRNAs**						
Sm19	5029661	5029530			ncMSMEG14931Ac	100–118
Sm32/33#	417709	417796	✓		ncMSMEG10373A	44
Sm35	1458488	1458562	✓		ncMSMEG11363A	118
Sm46	5864890	5864989	✓		ncMSMEG15796A	82
Sm49	1086797	1087035			ncMSMEG11016A	48–66
Sm64	2523008	**2522888**	✓	✓	ncMSMEG12439Ac	100–118
Sm76	**3690377**	**3690280**	✓	✓	ncMSMEG13628Ac	100–118
Sm82	4392939/4392970	**4393039**	✓		ncMSMEG14302A	66/82–100
**Total overlapping sense sRNAs**						
Sm38	2236980	2237466	✓		ncMSMEG2161A	48–66/66–82
Sm41	3815700/3815647	**3815581**	✓		ncMSMEG3749Ac	48–66/100–118
Sm90	6845964	6846035	✓	✓	ncMSMEG6799A	48–66
Sm93	**858482**	**858588**	✓		ncMSMEG0774A	66–82/100–118
**Total overlapping antisense sRNAs**						
Sm42	4290417/4290487	**4290537**	✓	✓	ncMSMEG4206A	48–66/100–118
Sm67	2600405/2600425	**2600485**			ncMSMEG2514A	48–66/66–82
Sm68	2600389	2600701			ncMSMEG2514B	82–100
Sm74	3111233	3111268	✓		ncMSMEG3037A	66–82
**Partial overlapping antisense sRNAs**						
Sm11	2835860	**2835984/2835999**	✓		ncMSMEG12771A	100–118/140–151
**Not categorized sRNAs**						
Sm47	6242319	6242668	✓		ncMSMEG16173A	311

* The coordinates in bold were determined by 5′ or 3′ Deep-RACE. Where only one end was determined by Deep RACE, the other end was estimated based on the size determined by Northern blot. Where neither end was determined by Deep RACE, SIPHT-predicted coordinates are listed (underlined text).

# Experimentally demonstrated in previous study [27].

Given the practical convenience of testing RNA from both species simultaneously to search for novel sRNA candidates, we used the designed probes for sRNA detection in *M. smegmatis* to also probe expression of these candidates in *M. bovis* BCG and *M. tuberculosis*. Although our focus was to validate *M. smegmatis* predictions, we fortuitously discovered homologues of 9 candidates in *M. bovis* BCG and 4 candidates in *M. tuberculosis* (Table 1, Figure S2). Since these probes were not specifically designed for the other two species, lack of detection could be due to either the absence of sRNA expression or to non-optimization of the probe sequence that was used for hybridization to the targeted region in the *M. bovis* BCG and *M. tuberculosis* genome. Also, differences in culture medium might contribute to the low number of expressed homologous sRNAs of *M. smegmatis* in *M. tuberculosis* as expression of these sRNAs could be specific to different conditions in *M. tuberculosis*. Given our focus in sRNA identification, specific conditions that could lead to differences in sRNA expression will be explored in future work.

### 23 Novel sRNAs Identified in Mycobacterium Bovis BCG

**Figure 3:**
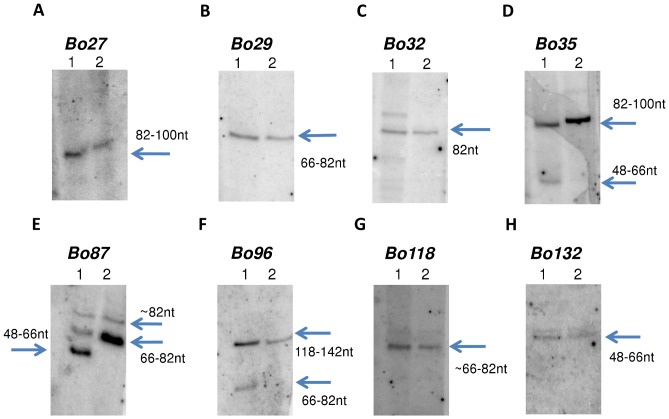
Northern blotting confirmation of sRNA candidates in *M. bovis* BCG. Selected images of Northern blotting analysis for validated *M. bovis* BCG sRNAs; the remaining images are included in Figure S2. Lane 1 and 2 indicate total RNA samples extracted from *M. bovis* BCG and *M. smegmatis*, respectively. We used Phi-X174/Hae III Marker for the size prediction. The probes we used for this analysis are listed in Table S1.

Twenty-one of the sRNA candidates for *M. bovis* BCG (Bo12, Bo15, Bo41, Bo52, Bo58, Bo67, Bo68, Bo75, Bo80, Bo85, Bo99, Bo100, Bo111, Bo113, Bo115, Bo117, Bo122, Bo125, Bo126, Bo137, and Bo139) were previously identified, under the nomenclature Mpr 1–21, respectively [13]. Forty-six other candidates were also tested previously but showed no signal; therefore, only the remaining 77 candidates were tested using Northern blotting analysis in this study, and we confirmed expression of 23 new sRNA candidates (Figure 3 and Figure S3). A homologue of Bo46 was previously identified in *M. tuberculosis* by RNA-seq [15] but not otherwise experimentally validated. All of the validated sRNAs were in the same orientation as that predicted by SIPHT. We also applied the probes to *M. smegmatis* and *M. tuberculosis* and identified 20 and 5 sRNA homologues, respectively (Table 2; Figure 3; Figure S3). All the confirmed sRNAs in *M. bovis* BCG and *M. tuberculosis* are listed in Table 2, along with the new nomenclature for sRNAs.

**Table 2:** sRNAs confirmed by Northern blotting analysis in *M. Bovis* BCG.

			Homology confirmed by Northern analysis in:			
	5′ end*	3′ end*	*M. smegmatis*	*M. tuberculosis*	New nomenclature	Length Confirm by Northern blotting analysis(nt)
**Intergenic sRNAs**						
Bo35	**576179**	**576067/576104**	✓		ncBCG10493Ac	48–66/82–100
Bo48	3028936	**3028876**	✓	✓	ncBCG12782Ac	48–66
Bo53	1044606	**1044706/1044720/1044727**	✓		ncBCG10960A	82–100/100–118
Bo60	1247638	**1247538**	✓		ncBCG11150Ac	66/100
Bo71	1588853	1588693	✓	✓	ncBCG11448Ac	66
Bo73	1647817	1647853	✓		ncBCG11504A	66–82
Bo78	**207337**	**207179**	✓	✓	ncBCG10186Ac	66/100
Bo86	2325795	2325960	✓		ncBCG12107A	66–82
Bo101	2919337	**2919277**	✓		ncBCG2654Ac	48–66
Bo118	3765977	**3765917**	✓		ncBCG13438Ac	48–66
Bo132	4260533	**4260610**	✓		ncBCG13885A	66–82
Bo105	3073445	3073541			ncBCG12831A	66–82
**Partial overlapping sense sRNAs**						
Bo27	**2157804**	2157704	✓		ncBCG11948Ac	82–100
Bo46	2603016	**2602916**	✓	✓	ncBCG12368Ac	100
Bo82	2235286	**2235196**	✓		ncBCG12024Ac	82–100
Bo87	**2351000/2351046**	**2350915/2350874**	✓	✓	ncBCG12128Ac	48–66/66–82/82
**Partial overlapping antisense sRNAs**						
Bo32	**817571**	**817483**	✓		ncBCG10734Ac	66–82/82
Bo47	**2705925/2705838**	**2705735**			ncBCG12462Ac	82–100
Bo81	218700	**2187796**	✓		ncBCG10195A	66/100
Bo96	**2686849**	2686909/2686989	✓		ncBCG12441A	66–82/118–142
Bo130	413416	413288	✓		ncBCG0352Ac	66–82/118–142
**Not categorized sRNAs**						
Bo13	3126934	3127070			ncBCG12882A	48–66/82–100
Bo29	1770012	1769806	✓		ncBCG11603Ac	66–82

* The coordinates in bold were determined by 5′ or 3′ Deep-RACE. Where only one end was determined by Deep RACE, the other end was estimated based on the size determined by Northern blot. Where neither end was determined by Deep RACE, SIPHT-predicted coordinates are listed (underlined text).

### Deep-RACE Identifies sRNA 5′ and 3′ Ends

**Figure 4:**
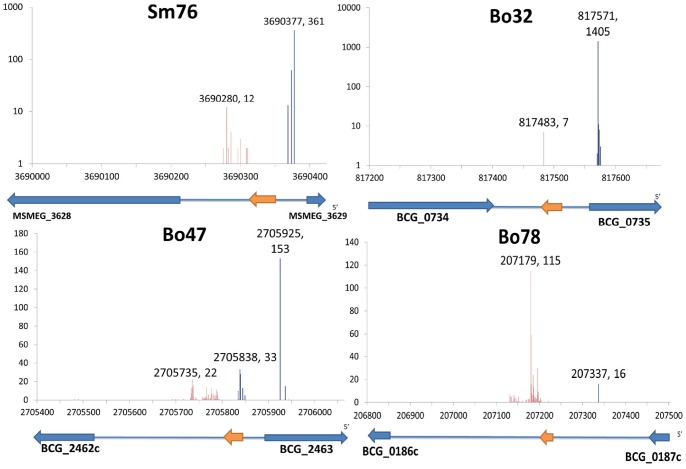
Identification of sRNA 5′ and 3′ ends by Deep RACE. Blue lines show the number of 5′ RACE reads mapped to respective genome, while red lines show the number of 3′ RACE reads. The coordinates with the highest number of mapped reads (the peak) indicate the likely 5′ and 3′ ends of sRNAs and are labeled in the figure. The orange arrow under the chart shows where the Northern probes base-paired and the blue arrows are the adjacent annotated coding regions. Results for other sRNAs can be found in Figure S4.

We used Deep-RACE, a previously described approach that combines conventional RACE and deep sequencing to identify 5′ and 3′ ends of selected RNAs [18], [28]. In total, we identified 5′ ends for 9 sRNAs and 3′ ends for 21 sRNAs. Examples are shown in Figure 4. For some sRNAs we identified multiple 5′/3′ ends. Multiple 5′ ends could be due to multiple transcription start sites or RNA processing. Multiple 3′ ends could be due to RNA processing or may indicate imprecise Rho-dependent termination of transcription.

### Size Comparisons between Experimental and Prediction Analysis

As noted in our earlier study [13], the predicted size of the candidate sRNAs correlates only weakly with experimental observations. Only about 17% of the confirmed sRNAs were within 10% of their predicted sizes. Additionally, in many cases, multiple bands were detected by Northern analysis, suggesting the presence of multiple start sites, multiple termination sites, and/or sRNA processing. This is consistent with the Deep RACE data (Figure 4; Figure S4). Deep RACE identified both 5′ and 3′ ends for seven sRNAs. In these cases, the sizes determined by Deep RACE are similar to those confirmed by Northern blotting.

### Location of sRNAs with Respect to Genes

To investigate the potential roles of the novel sRNAs, we mapped them all to the latest annotated genome (National Center for Biotechnology Information, NCBI). Although we aimed to find intergenic sRNAs, half of the candidates we identified in this study overlap partially or entirely protein-coding genes in either the sense or antisense orientation (Table 1, Table 2). We categorized sRNAs into different classes according to their position relative to adjacent coding regions. Where possible, we used 5′/3′ end information from Deep-RACE data. For sRNAs that have only one end mapped by Deep-RACE, the other end was estimated according to the length confirmed by Northern blotting analysis (Figure 4). For sRNAs that have neither end mapped by Deep-RACE, the farthest possible ends were estimated according to Northern blotting analysis and the sRNAs would be categorized as “not determined” if multiple class options exist.

Nine sRNAs in *M. smegmatis* (Sm19, Sm32/33, Sm35, Sm46, Sm49, Sm64, Sm76, Sm82) and twelve sRNAs in *M. bovis* BCG (Bo35, Bo48, Bo53, Bo60, Bo71, Bo73, Bo78, Bo86, Bo101, B0105, Bo118, Bo132) were mapped completely to intergenic regions. Four sRNAs in *M. smegmatis* (Sm38, Sm41, Sm90, Sm93) were mapped to the sense strand of annotated protein-coding genes, and four were mapped to the antisense strand (Sm42, Sm67, Sm68, Sm74). One sRNA in *M. smegmatis* (Sm11) and five in *M. bovis* BCG (Bo32, Bo47, Bo81, Bo96, Bo130) overlap partially with adjacent genes in the antisense orientation, and four sRNAs in *M. bovis* BCG (Bo27, Bo46, Bo82, Bo87) overlap partially with adjacent genes in the sense orientation. One sRNA in *M. smegmatis* (Sm47) and two in *M. bovis* BCG (Bo13, Bo29) were not classified.

The location of sRNAs relative to protein-coding genes also gives clues as to their function. Regulatory sRNAs that are completely intergenic typically function by base-pairing with distally-encoded mRNAs; however, some of the sRNAs are close to the 5′ end or 3′ end of adjacent genes, suggesting possible alternative regulatory roles. sRNAs antisense to ORFs or UTRs can regulate expression of the overlapping gene [29]. sRNAs located within UTRs or ORFs in the sense orientation may be degradation products or mRNAs or could be important *cis*-acting regulatory elements such as riboswitches.

sRNAs can be transcribed independently or generated by processing of mRNA UTRs. Several features of the sRNAs identified in this work are consistent with the sRNAs being independently transcribed from their own promoters. First, the Northern blots showed no evidence of larger bands that could correspond to pre-processed mRNAs. Second, 13 sRNAs (Sm35, Sm42, Sm67, Sm68, Sm74, Bo13, Bo32, Bo60, Bo71, Bo73, Bo81, Bo118, Bo130) are orientated away from the surrounding genes. Third, 5 sRNAs (Sm64, Sm82, Bo47, Bo105, Bo132) are located >200 bp from the nearest gene start/stop. Nineteen sRNAs are close to (<200 bp) upstream or downstream coding regions (Sm11, Sm19, Sm32/33, Sm46, Sm47, Sm49, Sm76, Bo27, Bo29, Bo35, Bo46, Bo48, Bo53, Bo78, Bo82, Bo86, Bo87, Bo96, Bo101) and four (Sm38, Sm41, Sm90, Sm93) overlap coding regions in the sense orientation. It is formally possible that these sRNAs are generated by mRNA processing or premature termination, although the Northern blot analysis argues against this. Regardless, sRNAs processed from mRNAs could still have important regulatory functions [3], [30], [31]. Indeed, a recent study identified 3′ UTRs as an abundant source of regulatory sRNAs in *Salmonella enterica* [32]. Alternatively, sRNAs generated by processing of mRNAs could indicate *cis*-acting regulatory elements such as riboswitches.

### Likely Regulators of sRNAs Identified by Analysis of ChIP-seq Datasets

**Figure 5:**
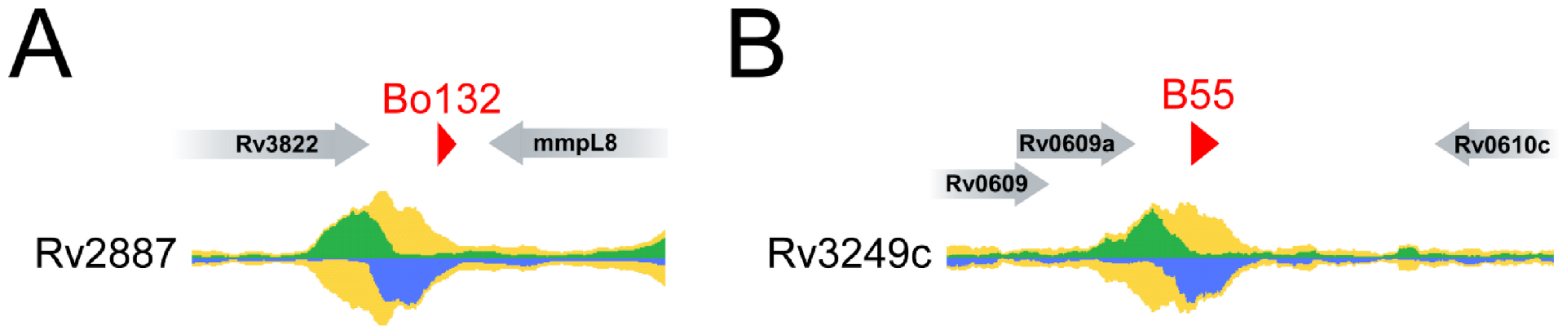
ChIP-seq peaks associated with predicted sRNA homologues in *M. tuberculosis*. ChIP-seq peaks that are unambiguously associated with sRNA 5′ ends. Raw ChIP-seq data from www.tbdb.org are shown for two transcription factors, (A) Rv2887, and (B) Rv3249c. Data are shown for genomic regions surrounding (A) sRNA Bo132 (this work), and (B) sRNA B55 [12]. The green and blue graphs indicate the relative number of sequence reads mapping to the plus and minus strands, respectively. The yellow graphs indicate the sum of plus and minus strand reads. Annotated genes are shown as gray arrows. sRNAs are shown as red triangles.

The regulation of sRNAs can provide important clues as to their biological functions. However, very little is currently known about regulation of mycobacterial sRNAs. The genome-wide binding profiles of many *M. tuberculosis* transcription factors have recently been determined using ChIP-seq and these data are publicly available [25]. Although we identified sRNAs in *M. bovis* BCG, it is highly likely that these sRNAs are conserved in *M. tuberculosis* given the extremely high similarity of the *M. bovis* BCG and *M. tuberculosis* genomes [33]. Hence, we searched existing ChIP-seq datasets of *M. tuberculosis* for transcription factors that bind close to sRNA 5′ ends, including sRNAs identified in earlier studies [12]. We identified 10 ChIP-seq peaks (indicative of a transcription factor binding site) located between 100 bp upstream and 20 bp downstream of sRNA 5′ ends (Table S5). Thus, we have identified likely examples of sRNA regulation. In some cases, the ChIP-seq peak is also close to the start of an annotated protein-coding gene. Hence, the transcription factor may regulate the protein-coding gene rather than the sRNA. Nevertheless, in four cases, the ChIP-seq peak is unambiguously associated with an sRNA 5′ end. The two examples with highest ChIP-seq signal are shown in Figure 5. For each of these examples, the transcription factor is otherwise uncharacterized.

## Conclusion

In summary, we have identified 17 novel sRNAs in *M. smegmatis* and 23 novel sRNAs in *M. bovis* BCG, verified 5′ and 3′ ends, and list these sRNAs according to a recently-proposed annotation nomenclature. Our analysis of sRNA position relative to protein-coding genes suggests various potential roles for these sRNAs in gene regulation. Although the specific biological function of these, and all other known mycobacterial sRNAs, is not understood, we speculate that some of these sRNAs contribute to the biology of pathogenic mycobacterial species. Future studies will focus on the functional characterization of these novel sRNAs.

## Supporting Information

Figure S1 Northern blotting analysis for *M. smegmatis* sRNAs.
(PDF)

Figure S2 Northern blotting analysis of sRNA candidates in *M. tuberculosis* with *M. smegmatis* and *M. bovis* BCG probes.
(PDF)

Figure S3 Northern blotting analysis for *M. bovis* BCG sRNAs.
(PDF)

Figure S4 Deep-RACE mapped reads of all sRNAs and adjacent gene annotations.
(PDF)

Table S1 The oligonucleotide sequence of all probes used for Northern Blotting analysis in this study.
(PDF)

Table S2 The oligonucleotide sequence of all primers used for Deep-RACE PCR.
(PDF)

Table S3 All 93 sRNA sequences predicted by SIPHT in *M. smegmatis*.
(PDF)

Table S4 All 144 sRNA sequences predicted by SIPHT in *M. bovis* BCG. This list excludes predicted tRNAs.
(PDF)

Table S5 All transcription factor ChIP-seq peaks located within 100 bp upstream and 20 bp downstream of sRNA 5′ ends.
(PDF)

## References

[1] WHO (2012) Global tuberculosis report 2012. WHO. p1.

[2] Gottesman S, Storz G (2011) Bacterial small RNA regulators: versatile roles and rapidly evolving variations. Cold Spring Harb Perspect Biol 3: a003798. 20980440 10.1101/cshperspect.a003798 PMC3225950

[3] Vogel J, Bartels V, Tang T, Churakov G, Slagter-Jäger J (2003) RNomics in *Escherichia coli* detects new sRNA species and indicates parallel transcriptional output in bacteria. Nucleic Acids Res 31: 6435–6443. 14602901 10.1093/nar/gkg867 PMC275561

[4] Gottesman S (2004) The small RNA regulators of Escherichia coli: roles and mechanisms. Annu Rev Microbiol 58: 303–328. 15487940 10.1146/annurev.micro.58.030603.123841

[5] Papenfort K, Vogel J (2010) Regulatory RNA in bacterial pathogens. Cell host & Microbe 8: 116–127. 20638647 10.1016/j.chom.2010.06.008

[6] Shinhara A, Matsui M, Hiraoka K, Nomura W, Hirano R, et al (2011) Deep sequencing reveals as-yet-undiscovered small RNAs in *Escherichia coli* . BMC Genomics 12: 428. 21864382 10.1186/1471-2164-12-428 PMC3175480

[7] Raghavan R, Groisman EA, Ochman H (2011) Genome-wide detection of novel regulatory RNAs in E. coli. Genome Res 21: 1487–1497. 21665928 10.1101/gr.119370.110 PMC3166833

[8] Albrecht M, Sharma CM, Reinhardt R, Vogel J, Rudel T (2010) Deep sequencing-based discovery of the Chlamydia trachomatis transcriptome. Nucleic Acids Res 38: 868–877. 19923228 10.1093/nar/gkp1032 PMC2817459

[9] Irnov I, Sharma CM, Vogel J, Winkler WC (2010) Identification of regulatory RNAs in Bacillus subtilis. Nucleic Acids Res 38: 6637–6651. 20525796 10.1093/nar/gkq454 PMC2965217

[10] Mitschke J, Georg J, Scholz I, Sharma CM, Dienst D, et al (2011) An experimentally anchored map of transcriptional start sites in the model cyanobacterium Synechocystis sp. PCC6803. Proc Natl Acad Sci U S A 108: 2124–2129. 21245330 10.1073/pnas.1015154108 PMC3033270

[11] Arnvig KB, Comas I, Thomson NR, Houghton J, Boshoff HI, et al (2011) Sequence-Based Analysis Uncovers an Abundance of Non-Coding RNA in the Total Transcriptome of *Mycobacterium tuberculosis* . PLoS Pathogens 7: e1002342. 22072964 10.1371/journal.ppat.1002342 PMC3207917

[12] Arnvig KB, Young DB (2009) Identification of small RNAs in *Mycobacterium tuberculosis* . Mol Microbiol 73: 397–408. 19555452 10.1111/j.1365-2958.2009.06777.x PMC2764107

[13] DiChiara JM, Contreras-Martinez LM, Livny J, Smith D, McDonough K a, et al (2010) Multiple small RNAs identified in *Mycobacterium bovis* BCG are also expressed in *Mycobacterium tuberculosis* and *Mycobacterium smegmatis* . Nucleic Acids Res 38: 4067–4078. 20181675 10.1093/nar/gkq101 PMC2896511

[14] Miotto P, Forti F, Ambrosi A, Pellin D, Veiga DF, et al (2012) Genome-wide discovery of small RNAs in *Mycobacterium tuberculosis* . PLoS one 7: e51950. 23284830 10.1371/journal.pone.0051950 PMC3526491

[15] Pellin D, Miotto P, Ambrosi A, Cirillo DM, Di Serio C (2012) A Genome-Wide Identification Analysis of Small Regulatory RNAs in *Mycobacterium tuberculosis* by RNA-Seq and Conservation Analysis. PLoS ONE 7: e32723. 22470422 10.1371/journal.pone.0032723 PMC3314655

[16] Pelly S, Bishai WR, Lamichhane G (2012) A screen for non-coding RNA in *Mycobacterium tuberculosis* reveals a cAMP-responsive RNA that is expressed during infection. Gene 500: 85–92. 22446041 10.1016/j.gene.2012.03.044 PMC3340464

[17] Livny J, Teonadi H, Livny M, Waldor MK (2008) High-throughput, kingdom-wide prediction and annotation of bacterial non-coding RNAs. PLoS ONE 3: e3197. 18787707 10.1371/journal.pone.0003197 PMC2527527

[18] Beauregard A, Smith E, Petrone B, Singh N, Karch C, et al (2013) Identification and characterization of small RNAs in *Yersinia pestis* . RNA Biol 10: 397–405. 23324607 10.4161/rna.23590 PMC3672283

[19] Lamichhane G, Arnvig KB, McDonough KA (2013) Definition and annotation of (myco)bacterial non-coding RNA. Tuberculosis (Edinburgh, Scotland) 93: 26–29. 10.1016/j.tube.2012.11.010 23291152

[20] Livny J (2012) Bioinformatic Discovery of Bacterial Regulatory RNAs Using SIPHT. In: Keiler KC, editor. Bacterial Regulatory RNA: Methods and Protocols. Totowa, NJ: Humana Press, Vol. 905: 3–14. 10.1007/978-1-61779-949-5_1 22735994

[21] Postic G, Frapy E, Dupuis M, Dubail I, Livny J, et al (2010) Identification of small RNAs in *Francisella tularensis* . BMC Genomics 11: 625. 21067590 10.1186/1471-2164-11-625 PMC3091763

[22] Khoo JS, Chai SF, Mohamed R, Nathan S, Firdaus-Raih M (2012) Computational discovery and RT-PCR validation of novel Burkholderia conserved and *Burkholderia pseudomallei* unique sRNAs. BMC Genomics 13 Suppl 7S13. 10.1186/1471-2164-13-S7-S13 PMC3521395 23282220

[23] Xia L, Xia W, Li S, Li W, Liu J, et al (2012) Identification and expression of small non-coding RNA, L10-Leader, in different growth phases of *Streptococcus mutans* . Nucleic Acid Ther 22: 177–186. 22468692 10.1089/nat.2011.0339

[24] Lu X, Goodrich-blair H, Tjaden B (2011) Assessing computational tools for the discovery of small RNA genes in bacteria. RNA 17: 1635–1647. 21768221 10.1261/rna.2689811 PMC3162329

[25] Galagan J, Lyubetskaya A, Gomes A (2013) ChIP-Seq and the complexity of bacterial transcriptional regulation. Curr Top Microbiol Immunol 363: 43–68. 22983621 10.1007/82_2012_257

[26] Li H, Durbin R (2009) Fast and accurate short read alignment with Burrows-Wheeler transform. Bioinformatics (Oxford, England) 25: 1754–1760. 10.1093/bioinformatics/btp324 PMC2705234 19451168

[27] Li SK, Ng PK, Qin H, Lau JK, Lau JP, et al (2012) Identification of small RNAs in *Mycobacterium smegmatis* using heterologous Hfq. RNA 19 74–84. 23169799 10.1261/rna.034116.112 PMC3527728

[28] Olivarius S, Plessy C, Carninci P (2009) High-throughput verification of transcriptional starting sites by Deep-RACE. Biotechniques 46: 130–132. 19317658 10.2144/000113066

[29] Georg J, Hess WR (2011) cis-antisense RNA, another level of gene regulation in bacteria. Microbiology and molecular biology reviews?: MMBR 75: 286–300. 21646430 10.1128/MMBR.00032-10 PMC3122628

[30] Kawano M, Reynolds AA, Miranda-Rios J, Storz G (2005) Detection of 5′- and 3′-UTR-derived small RNAs and cis-encoded antisense RNAs in Escherichia coli. Nucleic Acids Res. 33: 1040–1050. 10.1093/nar/gki256 PMC549416 15718303

[31] Loh E, Dussurget O, Gripenland J, Vaitkevicius K, Tiensuu T, et al (2009) A trans-acting riboswitch controls expression of the virulence regulator PrfA in Listeria monocytogenes. Cell 139: 770–779. 19914169 10.1016/j.cell.2009.08.046

[32] Chao Y, Papenfort K, Reinhardt R, Sharma CM, Vogel J (2012) An atlas of Hfq-bound transcripts reveals 3′ UTRs as a genomic reservoir of regulatory small RNAs. The EMBO journal 31: 4005–4019. 22922465 10.1038/emboj.2012.229 PMC3474919

[33] Garnier T, Eiglmeier K, Camus JC, Medina N, Mansoor H, et al (2003) The complete genome sequence of *Mycobacterium bovis* . PNAS 100: 7877–7882. 12788972 10.1073/pnas.1130426100 PMC164681

