# Supplementary material for: Identification of Novel sRNAs in Mycobacterial Species
Source: PLoS One. 2013 Nov 14;8(11):e79411. doi: 10.1371/journal.pone.0079411 (PMC3828370; doi:10.1371/journal.pone.0079411)

Supplementary Figure 1. Northern blotting analysis confirmation of sRNA candidates in *M. smegmatis*

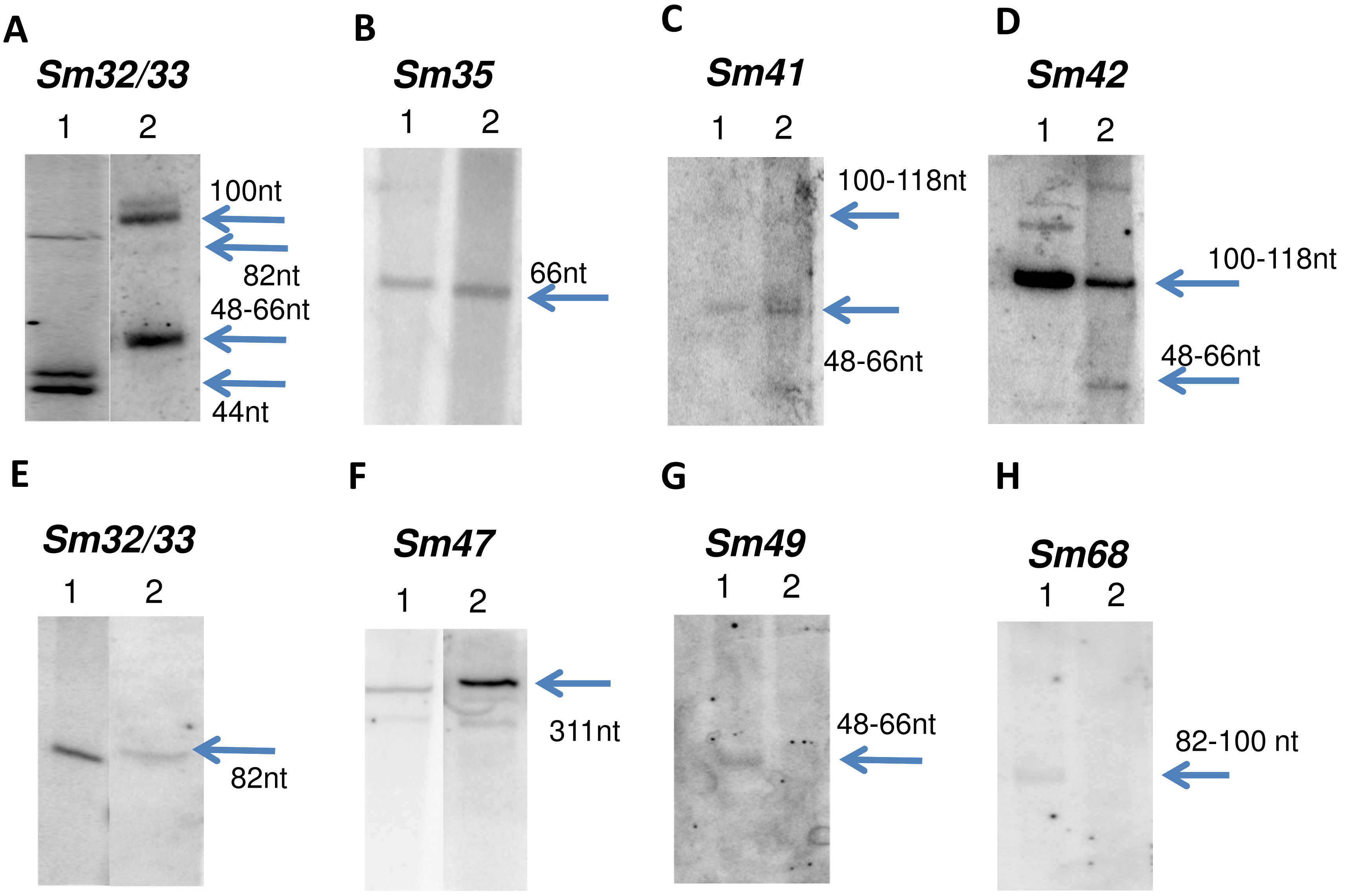

I

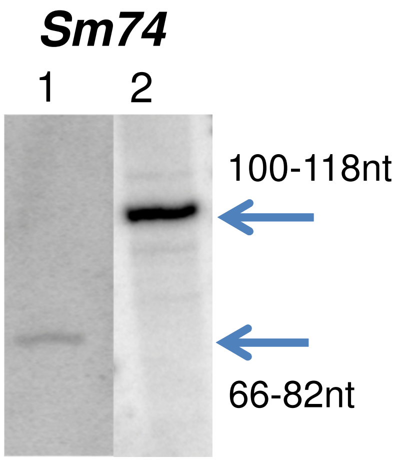

J

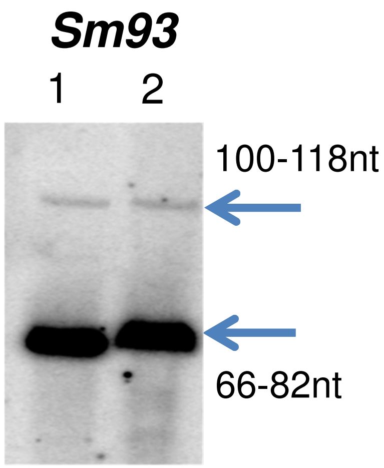

Supplement: Figure S1 — Northern blotting analysis for M. smegmatis sRNAs. (PDF) [file pone.0079411.s001.pdf]
