# Supplementary material for: Identification of Novel sRNAs in Mycobacterial Species
Source: PLoS One. 2013 Nov 14;8(11):e79411. doi: 10.1371/journal.pone.0079411 (PMC3828370; doi:10.1371/journal.pone.0079411)

Supplementary Figure 2. Northern blotting analysis confirmation of sRNA candidates in *M. tuberculosis* with *M. smegmatis* and *M. bovis* probes.

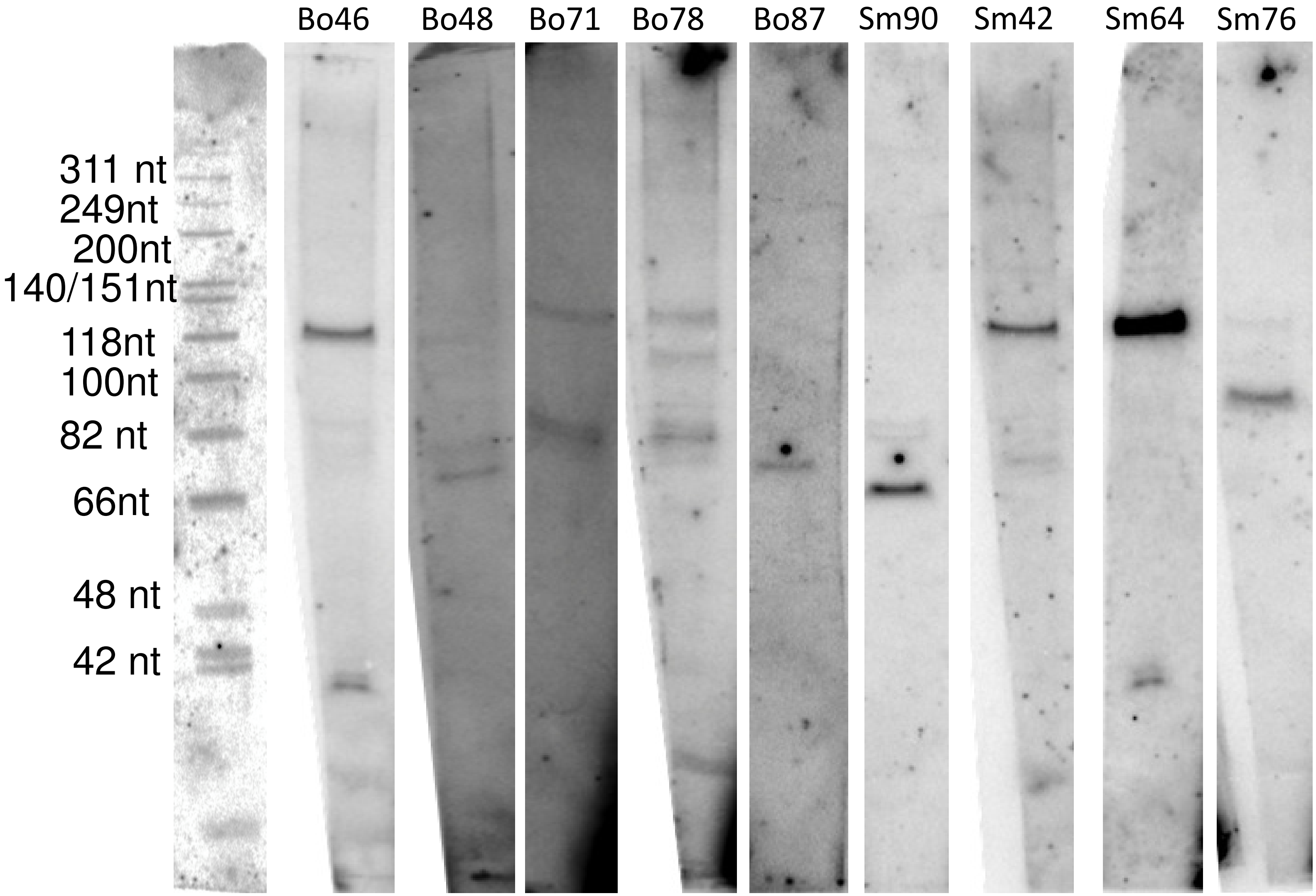

Supplement: Figure S2 — Northern blotting analysis of sRNA candidates in M. tuberculosis with M. smegmatis and M. bovis BCG probes. (PDF) [file pone.0079411.s002.pdf]
