# Supplementary material for: Identification of Novel sRNAs in Mycobacterial Species
Source: PLoS One. 2013 Nov 14;8(11):e79411. doi: 10.1371/journal.pone.0079411 (PMC3828370; doi:10.1371/journal.pone.0079411)

Supplementary Figure 3a. Northern blotting analysis confirmation of sRNA candidates in *M. bovis* BCG

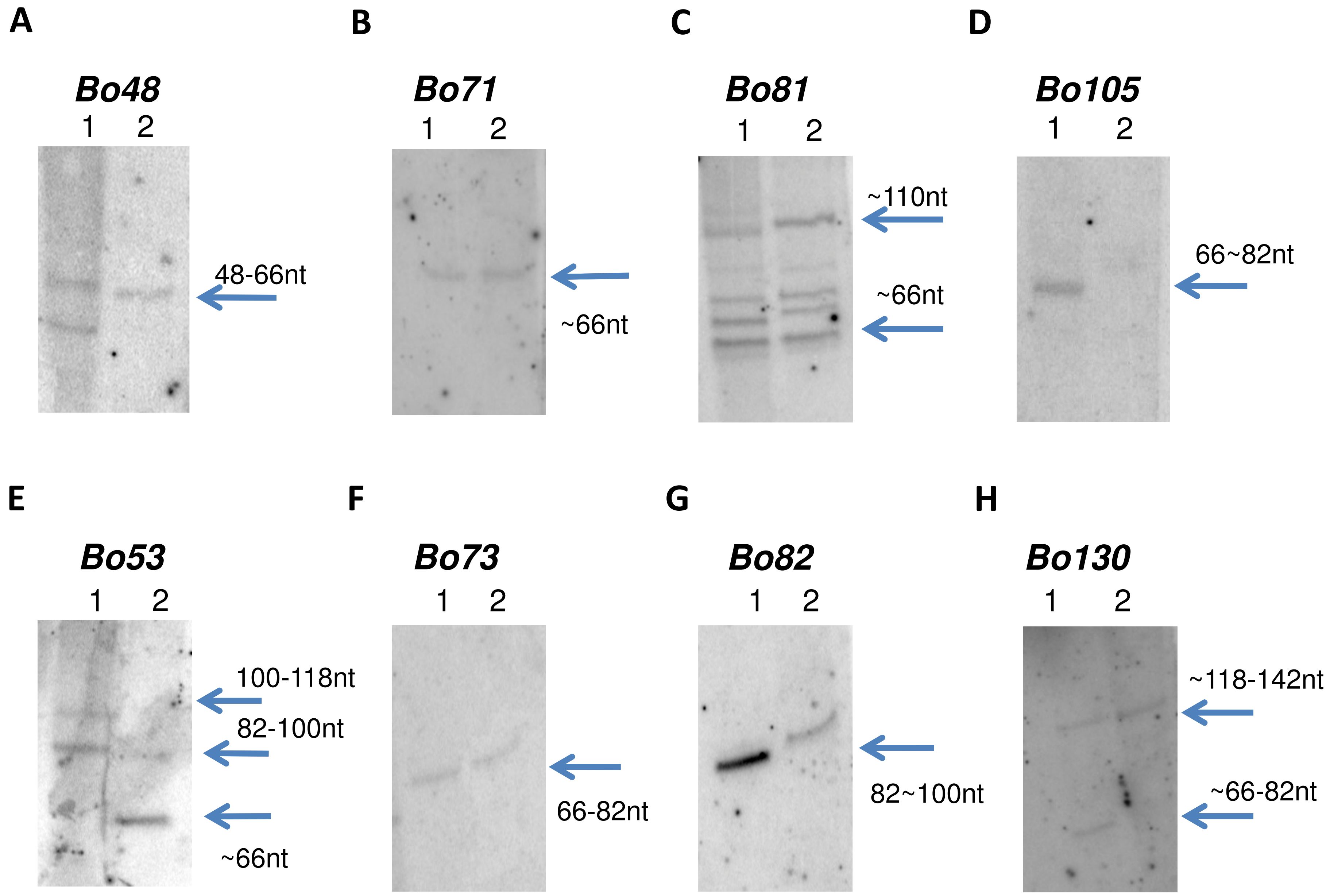

Supplementary Figure 3b. Northern blotting analysis confirmation of sRNA candidates in *M. bovis* BCG

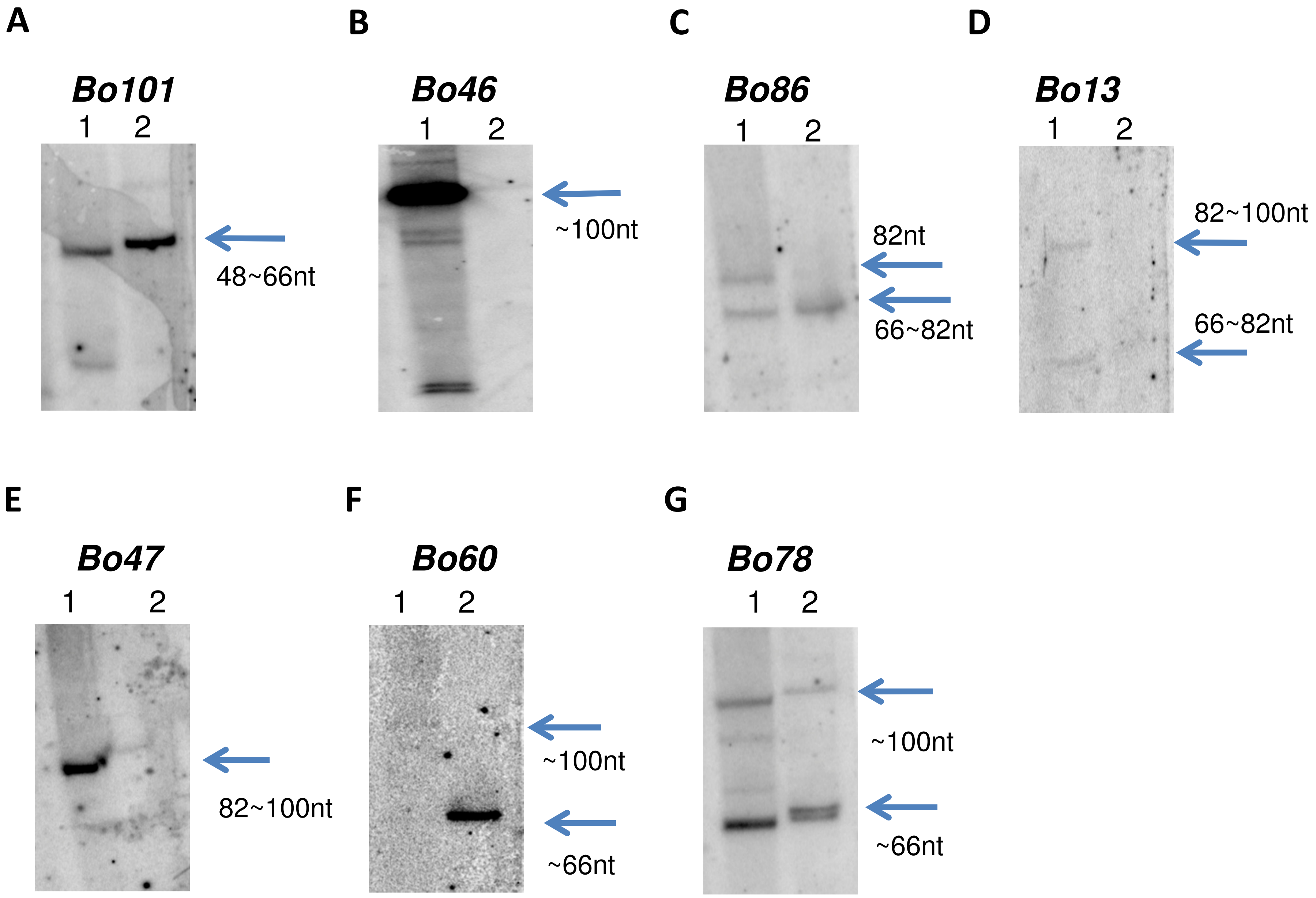

Supplement: Figure S3 — Northern blotting analysis for M. bovis BCG sRNAs. (PDF) [file pone.0079411.s003.pdf]
