# Supplementary material for: Identification of Novel sRNAs in Mycobacterial Species
Source: PLoS One. 2013 Nov 14;8(11):e79411. doi: 10.1371/journal.pone.0079411 (PMC3828370; doi:10.1371/journal.pone.0079411)
Supplement: Figure S4 — Deep-RACE mapped reads of all sRNAs and adjacent gene annotations. (PDF) [file pone.0079411.s004.pdf]

Sm11

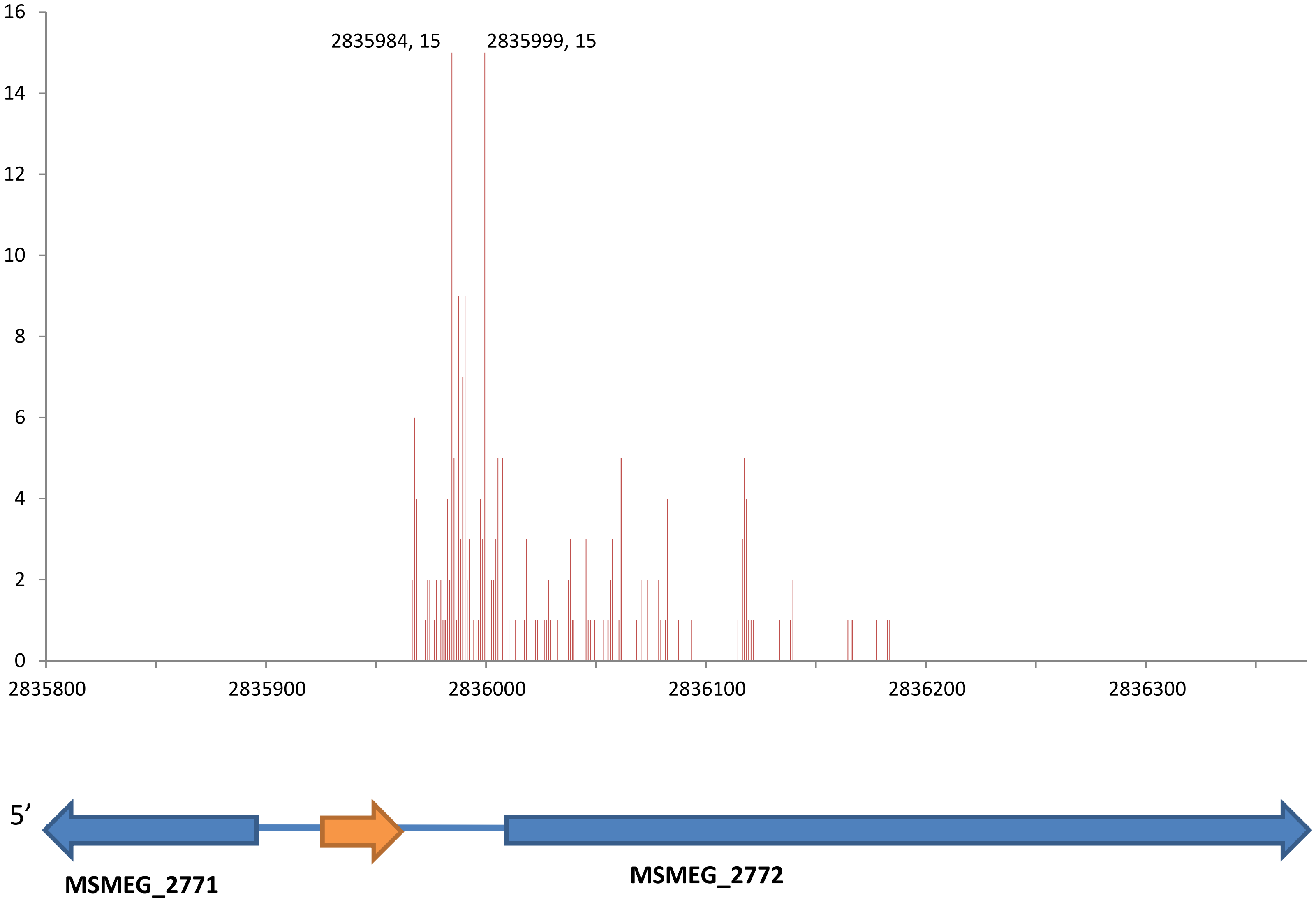

2835984, 15
2835999, 15
16
14
12
10
8
6
4
2
0
2835800
2835900
2836000
2836100
2836200
2836300
5’
MSMEG_2771
MSMEG_2772

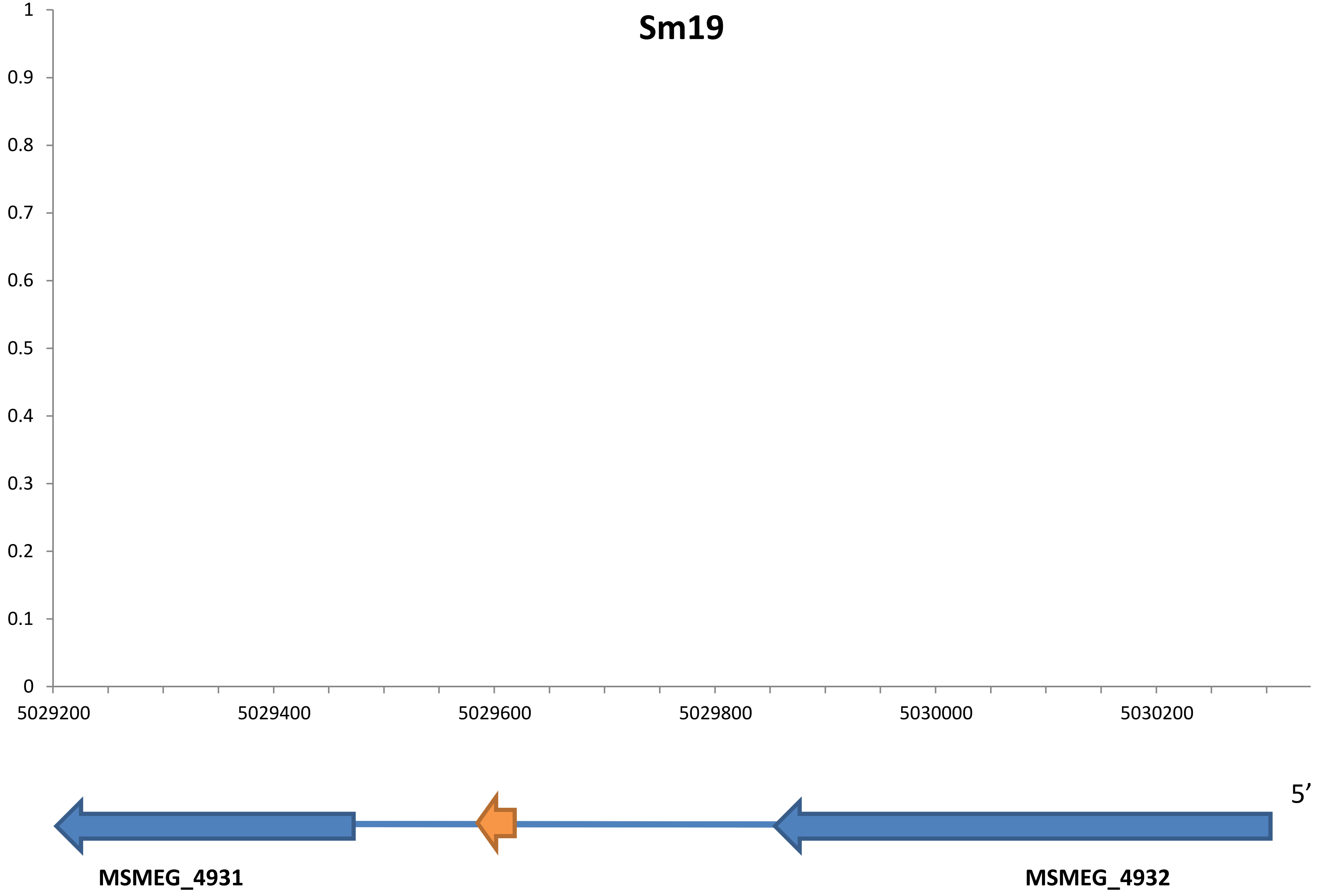
Sm19
1
0.9
0.8
0.7
0.6
0.5
0.4
0.3
0.2
0.1
0
5029200
5029400
5029600
5029800
5030000
5030200
5’
MSMEG_4931
MSMEG_4932

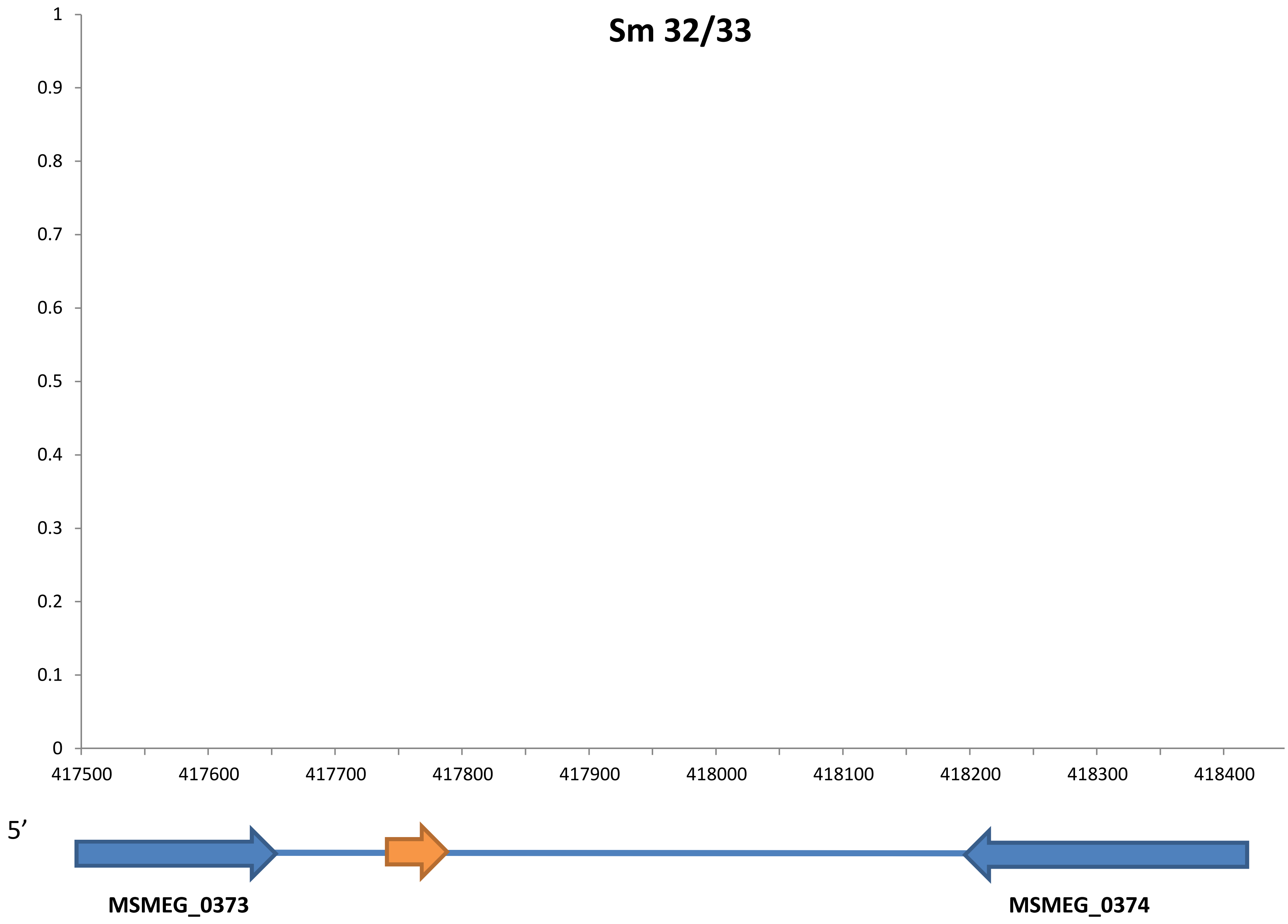
Sm 32/33
1
0.9
0.8
0.7
0.6
0.5
0.4
0.3
0.2
0.1
0
417500
417600
417700
417800
417900
418000
418100
418200
418300
418400
5’
MSMEG_0373
MSMEG_0374

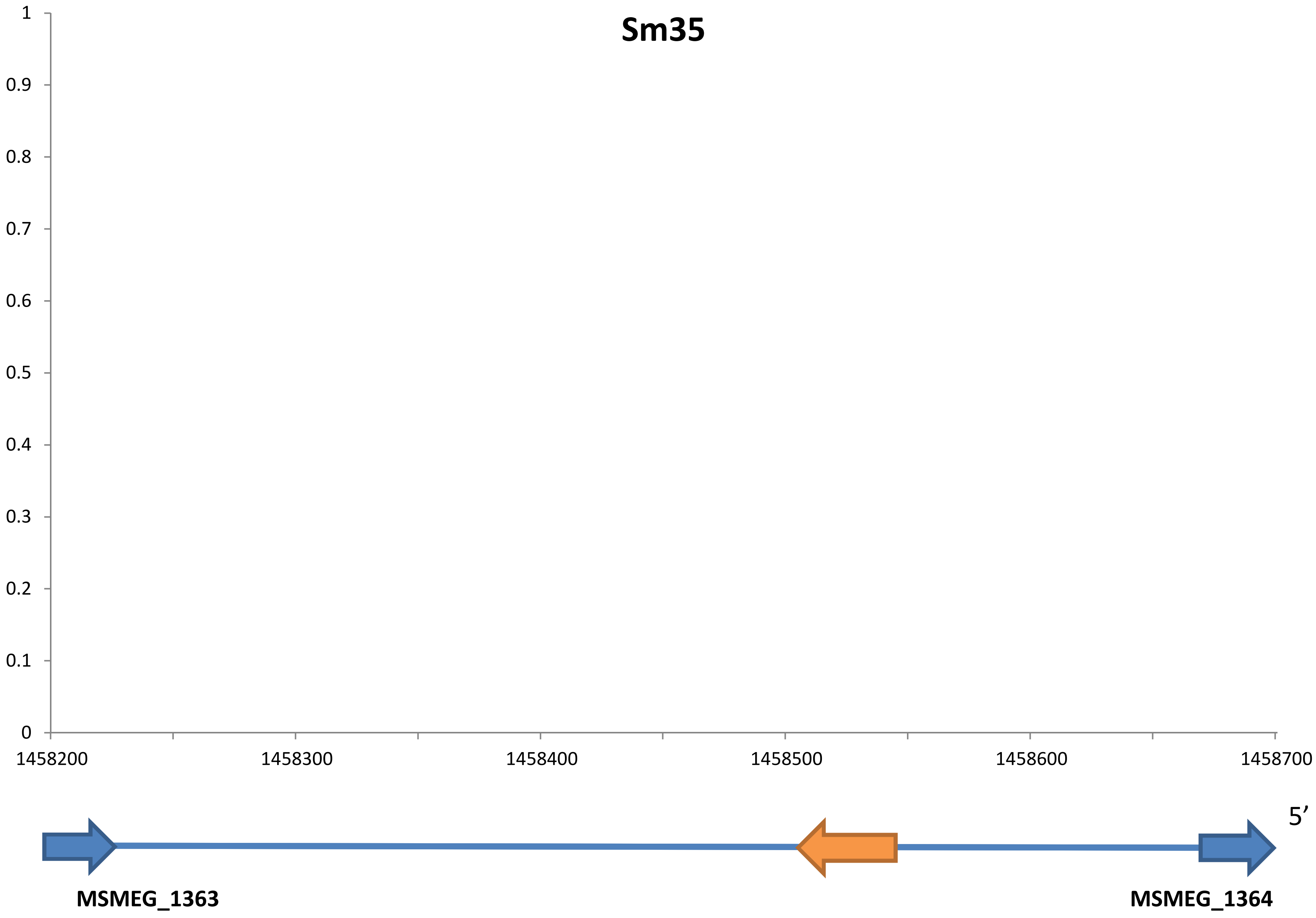
Sm35
1
0.9
0.8
0.7
0.6
0.5
0.4
0.3
0.2
0.1
0
1458200
1458300
1458400
1458500
1458600
1458700
5’
MSMEG_1363
MSMEG_1364

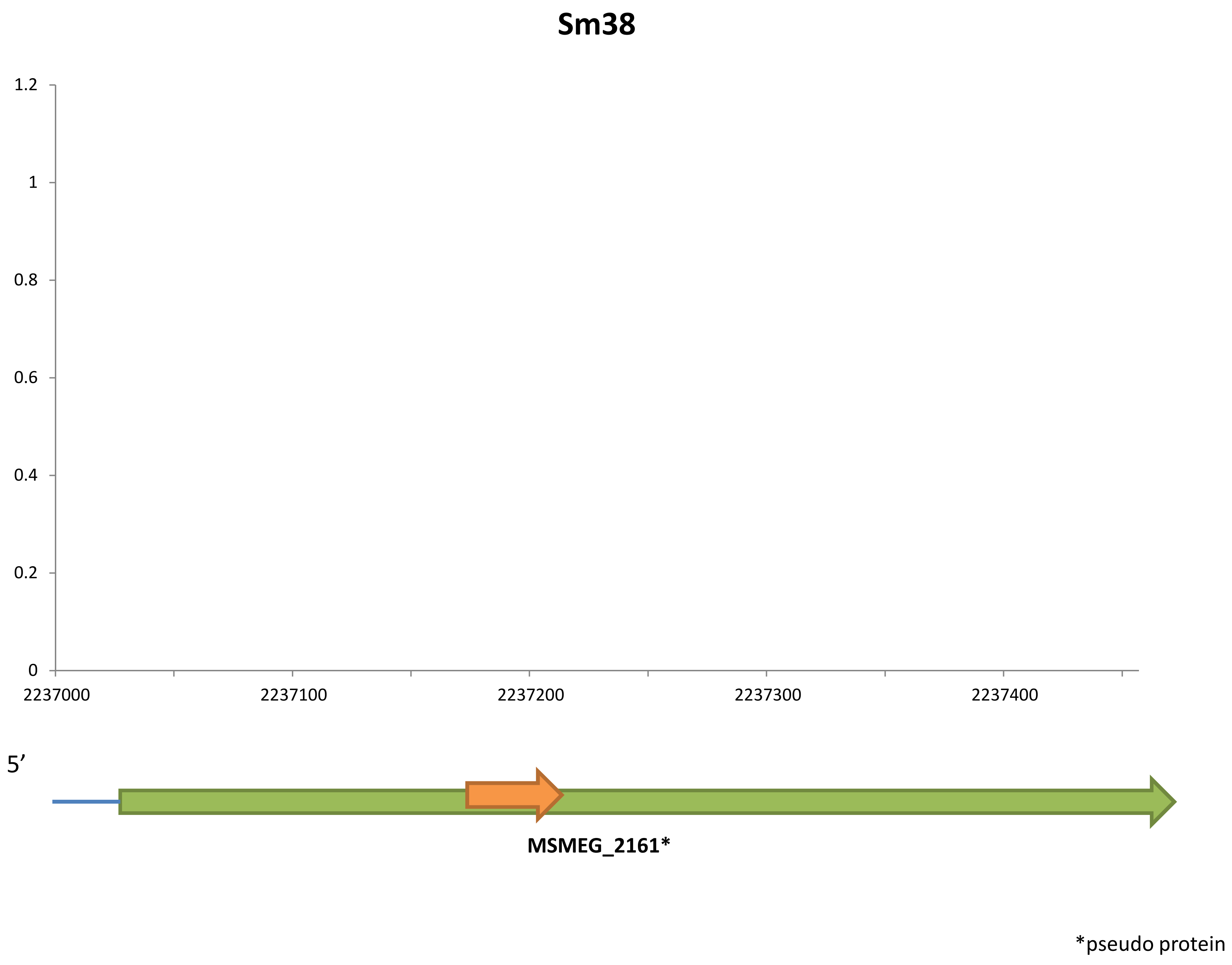
Sm38
1.2
1
0.8
0.6
0.4
0.2
0
2237000
2237100
2237200
2237300
2237400
5’
MSMEG_2161*
*pseudo protein

Sm41

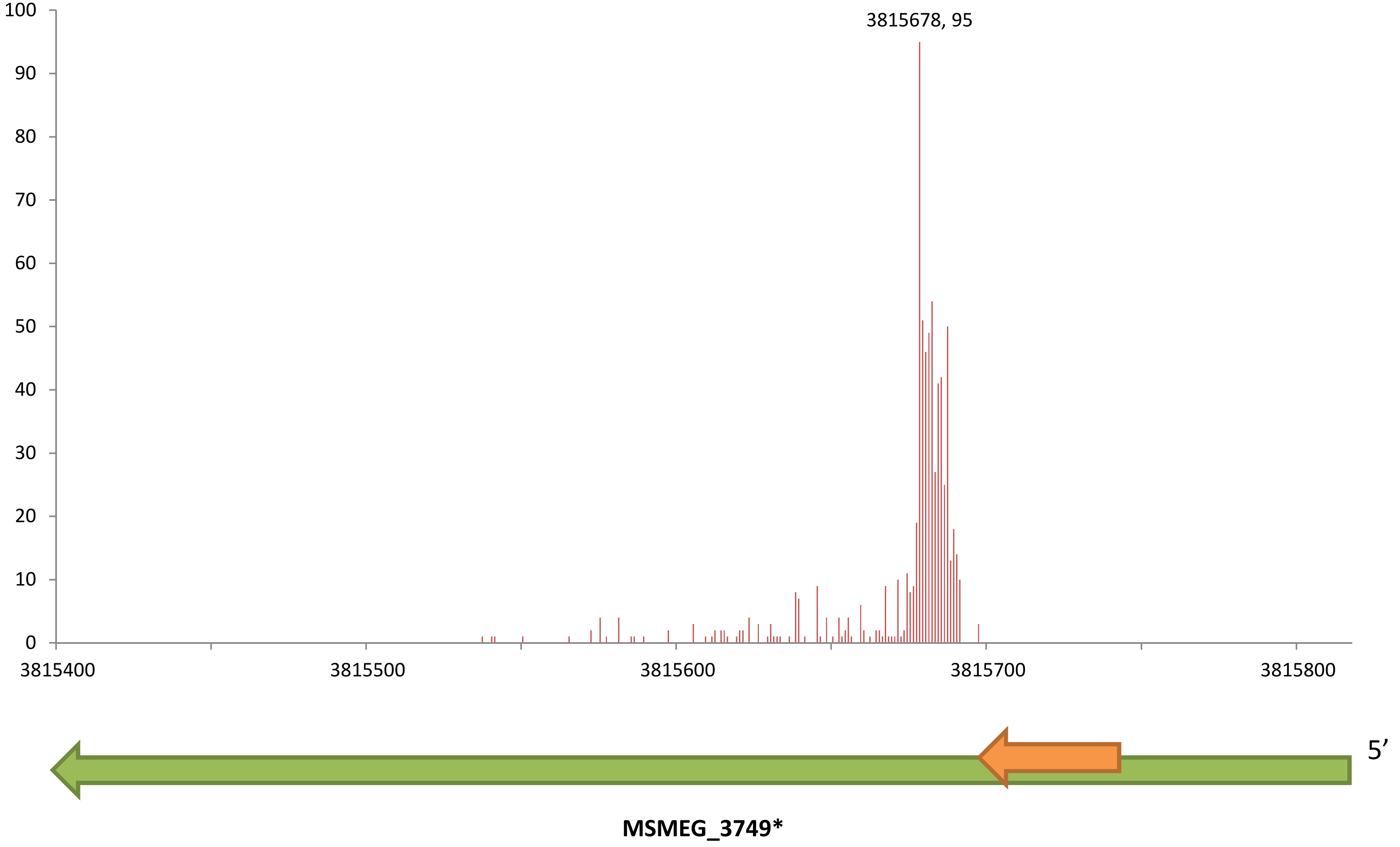

*pseudo protein

Sm42

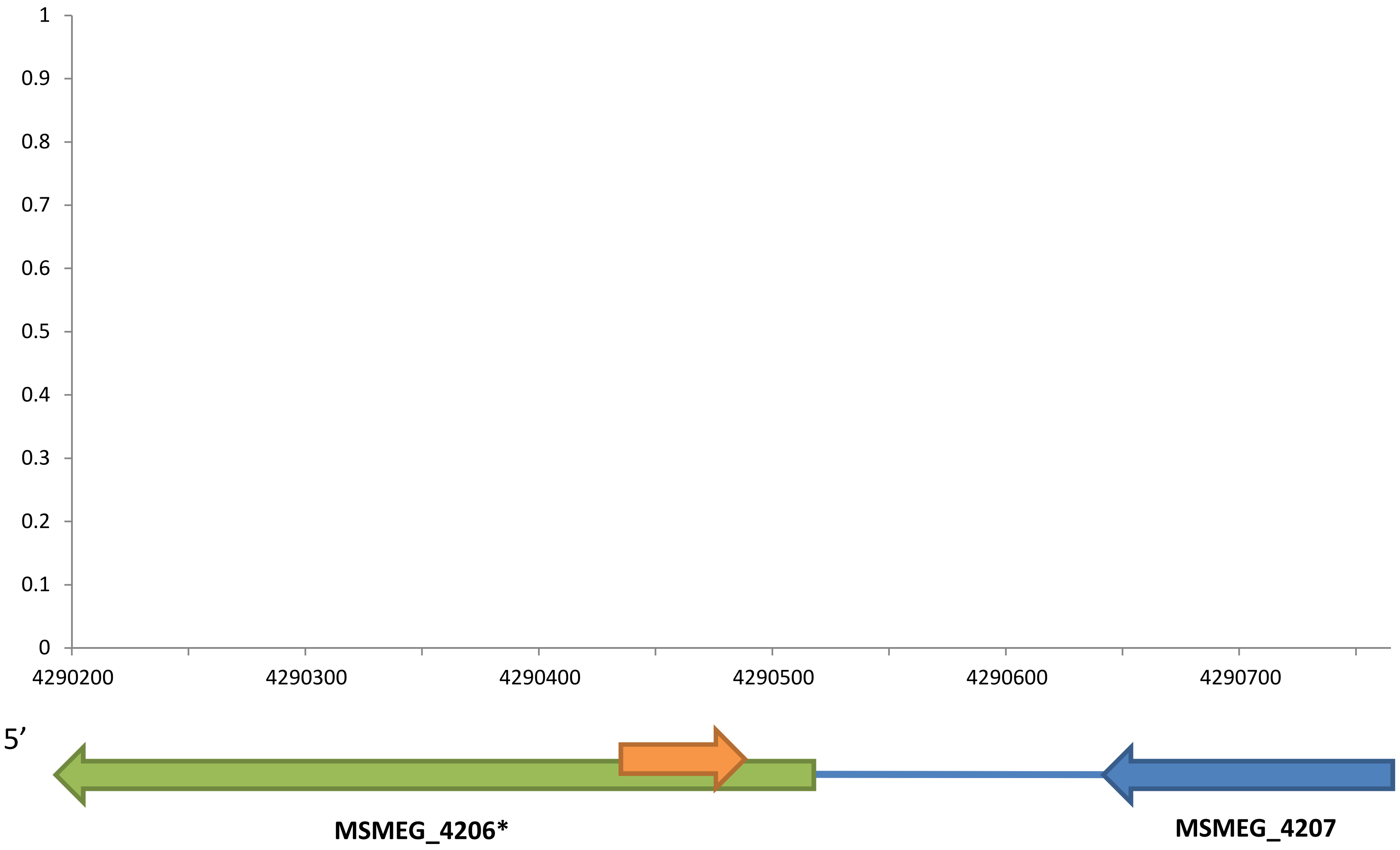

*pseudo protein

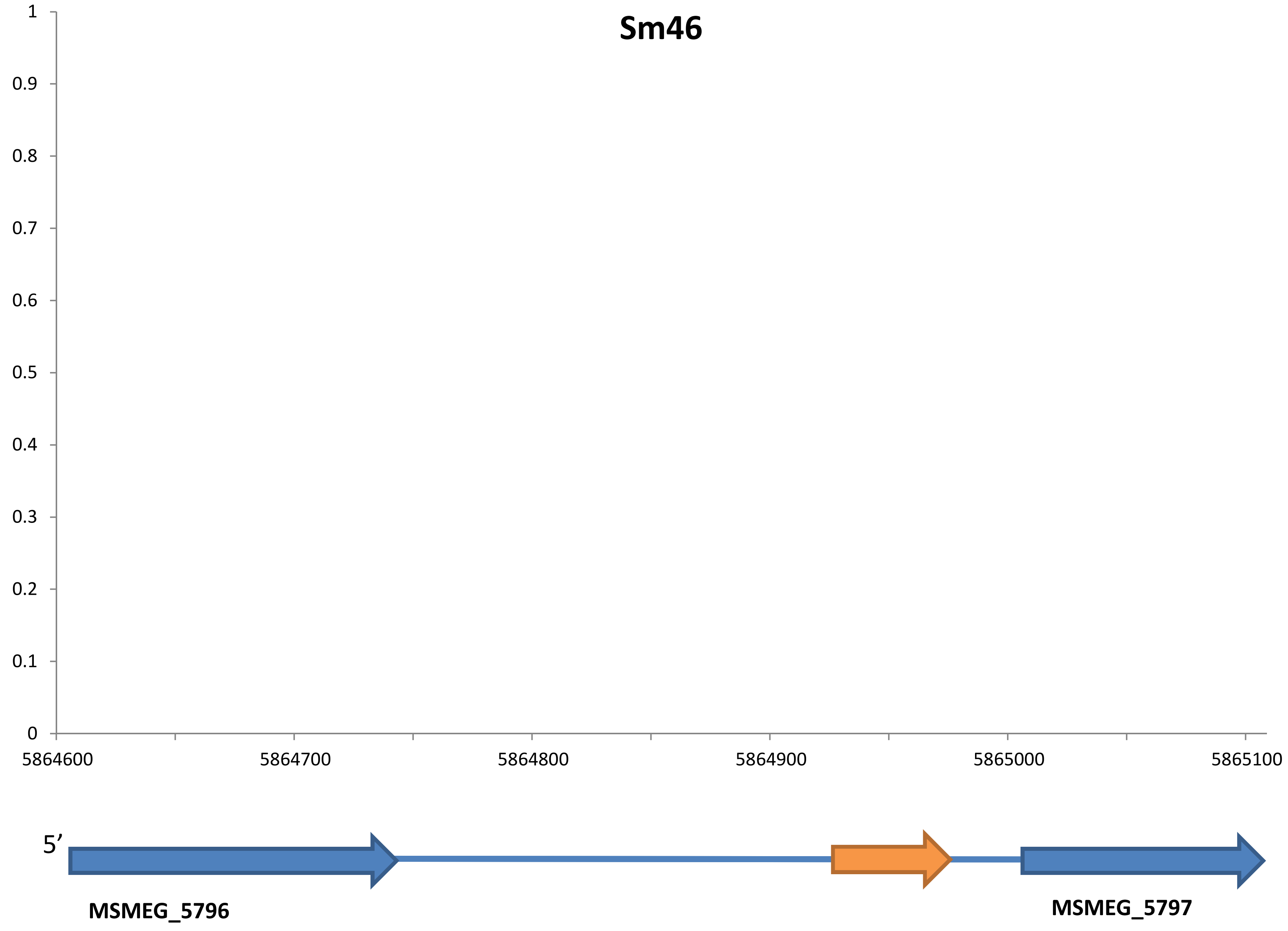
Sm46
1
0.9
0.8
0.7
0.6
0.5
0.4
0.3
0.2
0.1
0
5864600
5864700
5864800
5864900
5865000
5865100
5'
MSMEG_5796
MSMEG_5797

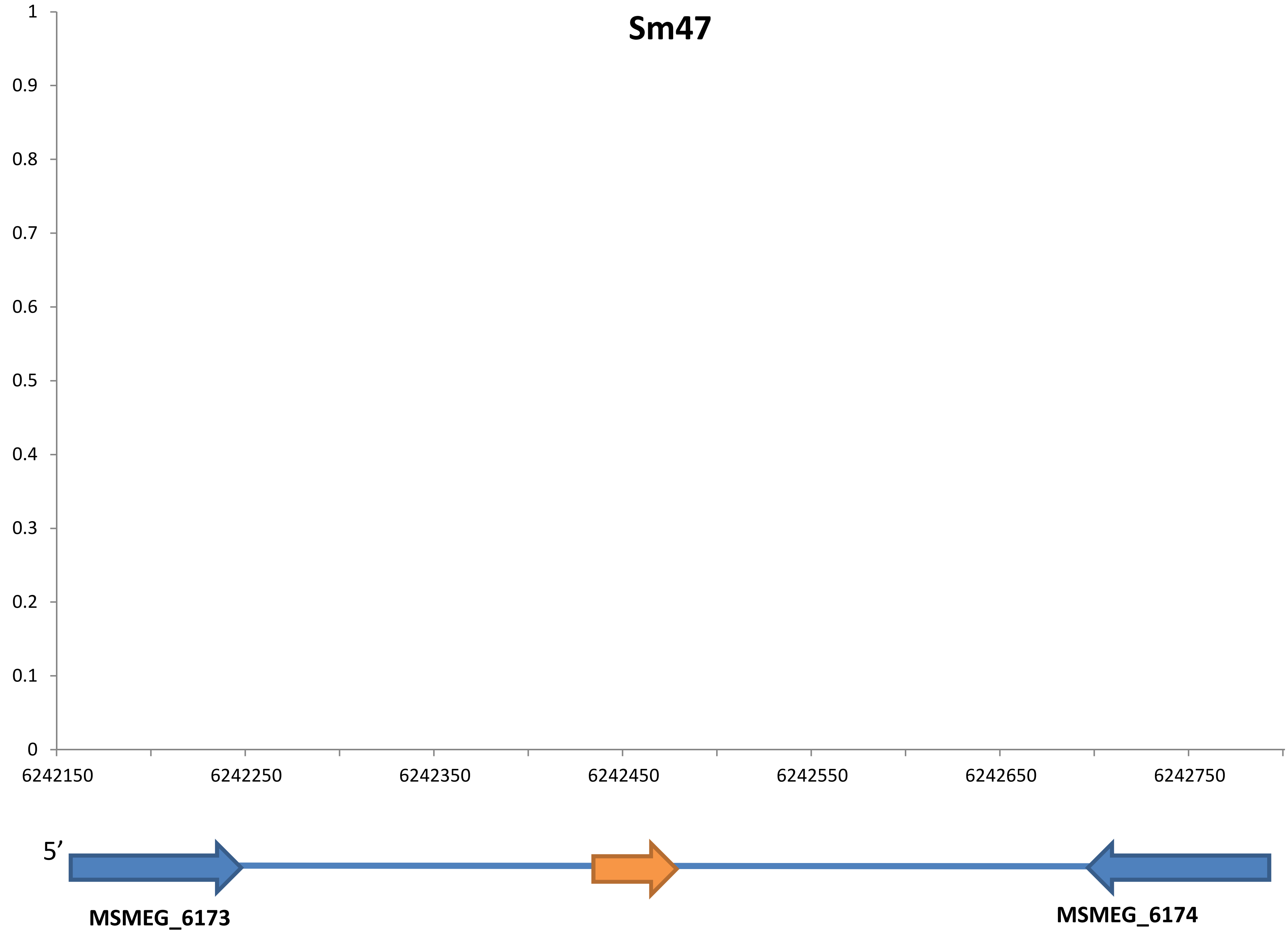
Sm47
1
0.9
0.8
0.7
0.6
0.5
0.4
0.3
0.2
0.1
0
6242150
6242250
6242350
6242450
6242550
6242650
6242750
5’
MSMEG_6173
MSMEG_6174

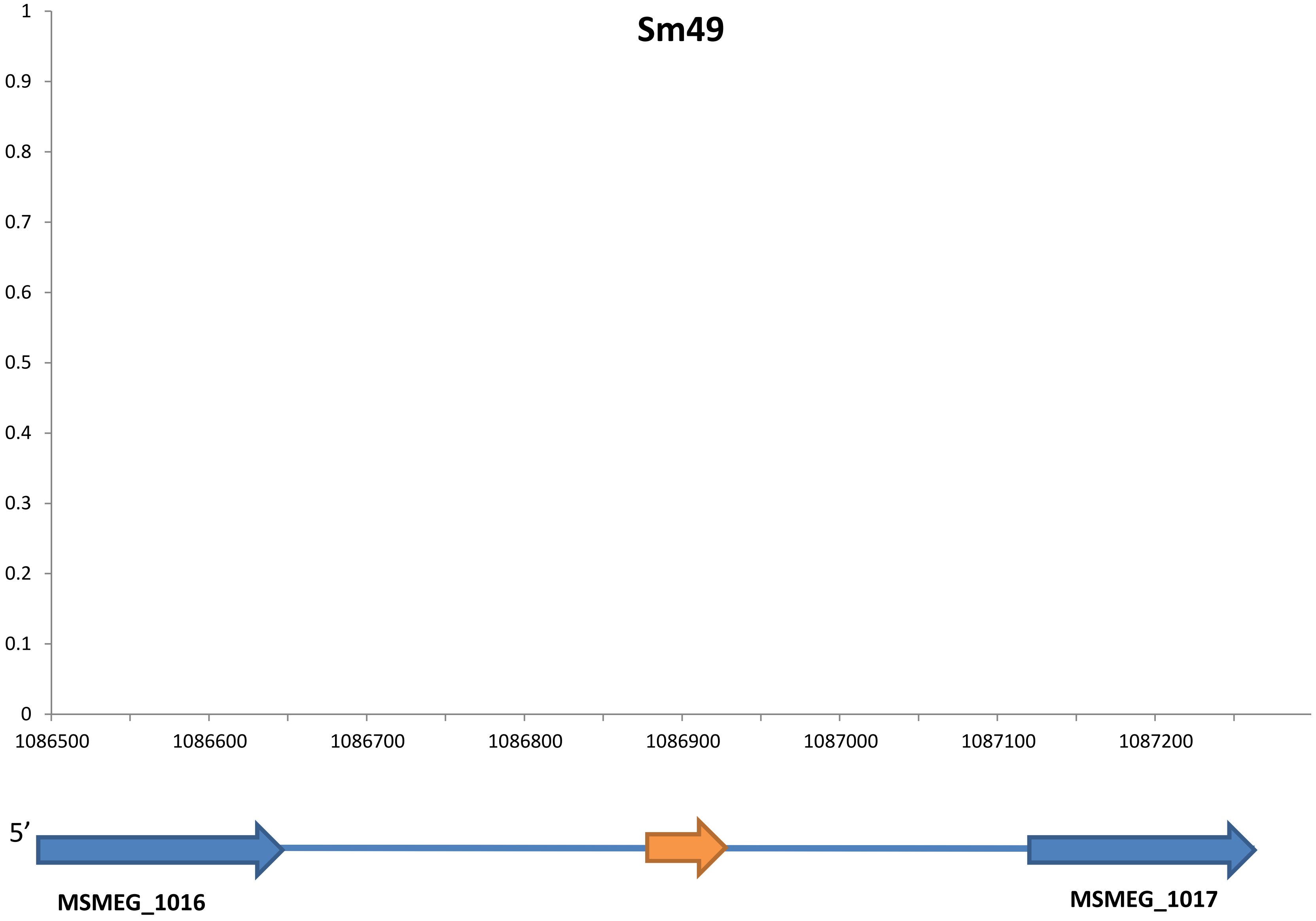
Sm49
1
0.9
0.8
0.7
0.6
0.5
0.4
0.3
0.2
0.1
0
1086500
1086600
1086700
1086800
1086900
1087000
1087100
1087200
5’
MSMEG_1016
MSMEG_1017

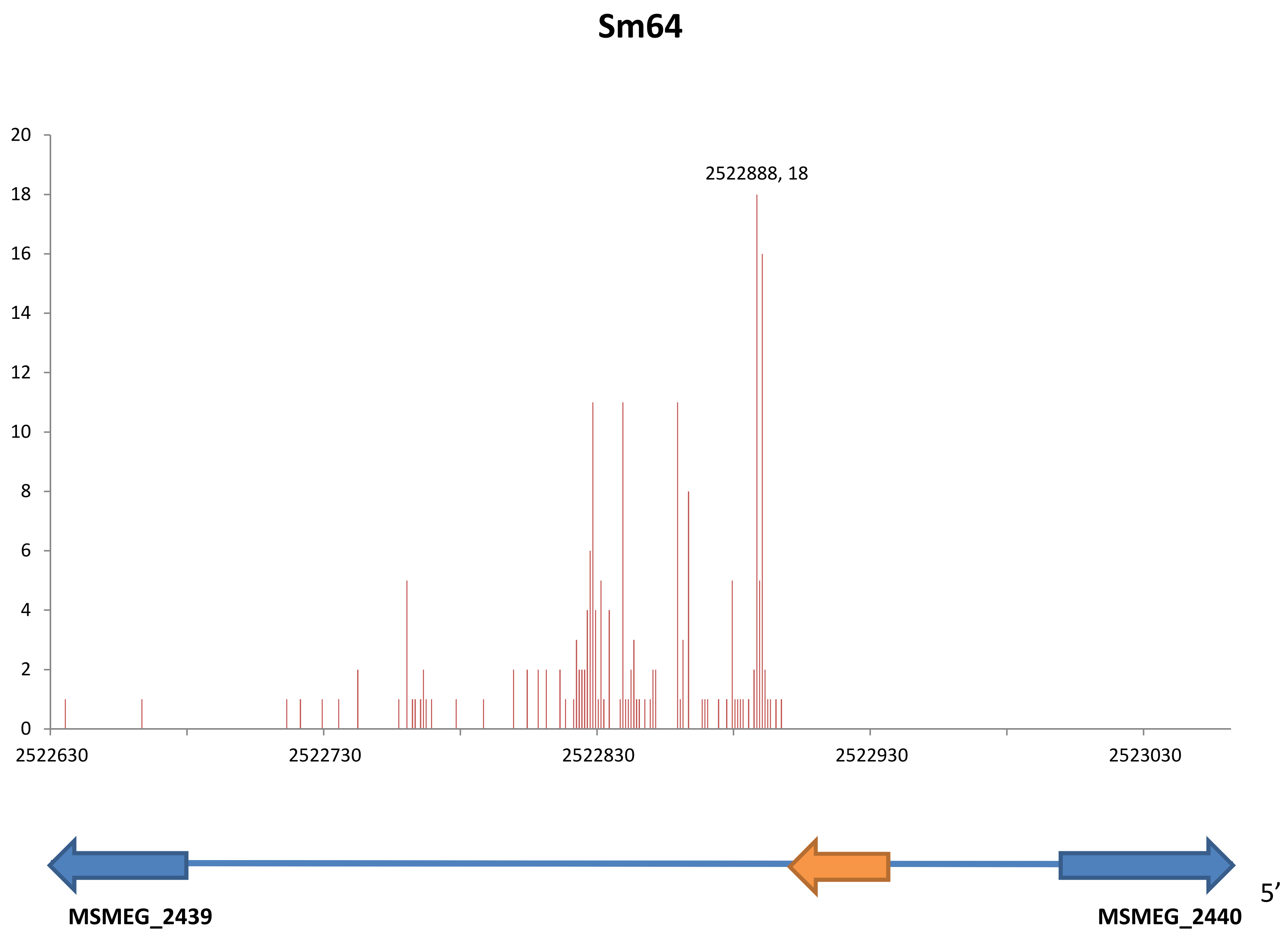
Sm64
2522888, 18
20
18
16
14
12
10
8
6
4
2
0
2522630
2522730
2522830
2522930
2523030
5’
MSMEG_2439
MSMEG_2440

Sm67/Sm68

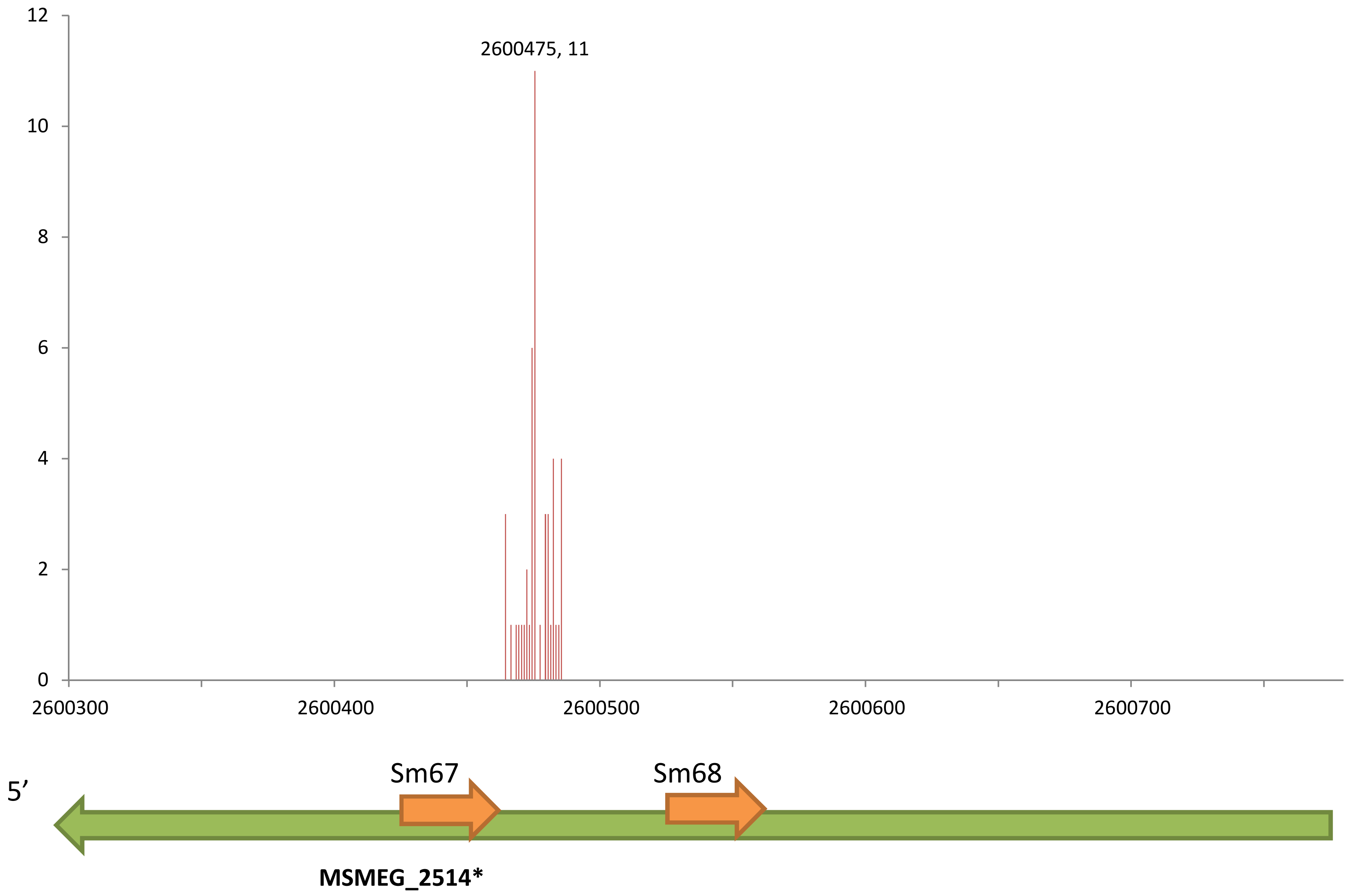

*pseudo protein

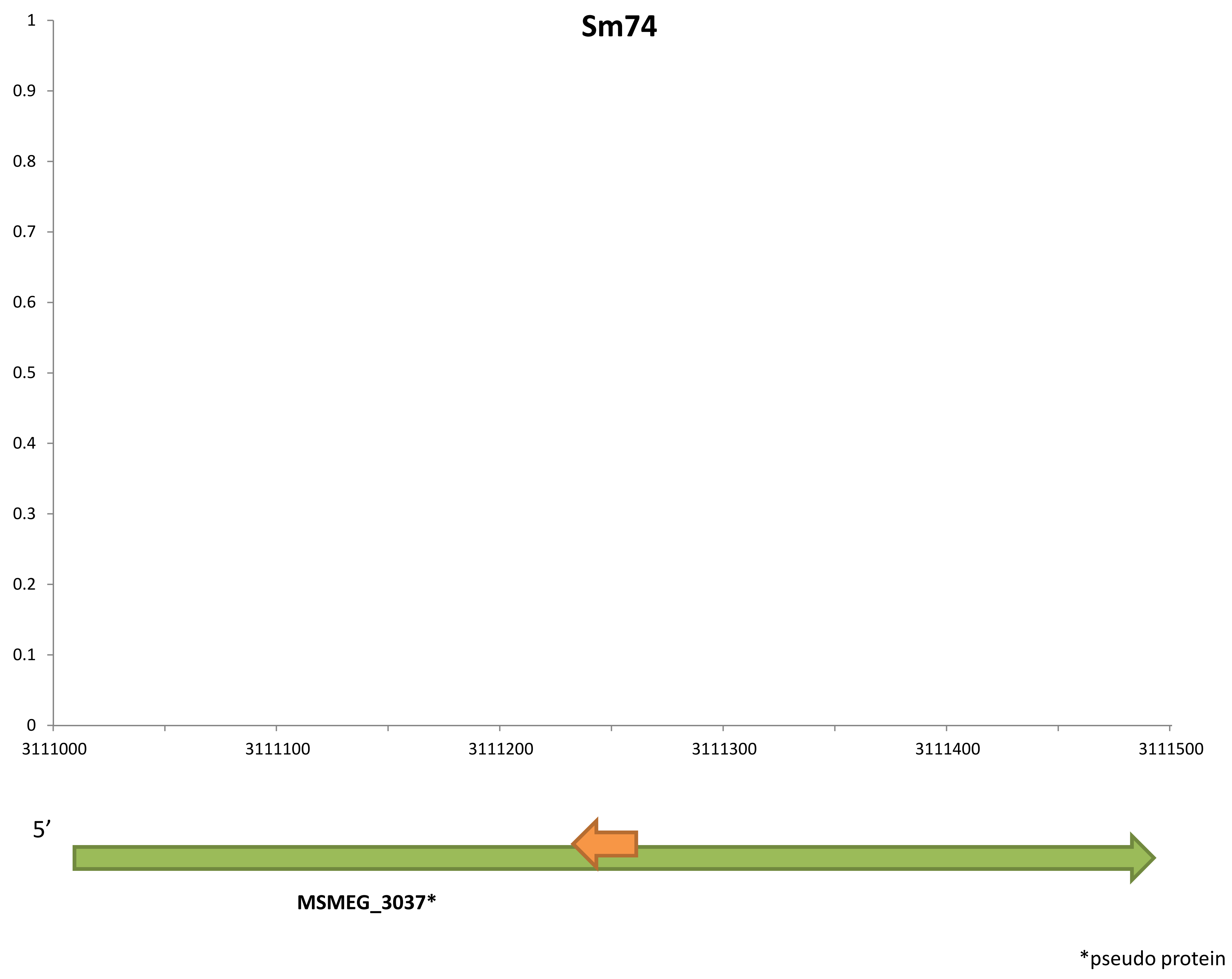
Sm74
1
0.9
0.8
0.7
0.6
0.5
0.4
0.3
0.2
0.1
0
3111000
3111100
3111200
3111300
3111400
3111500
5’
MSMEG_3037*
*pseudo protein

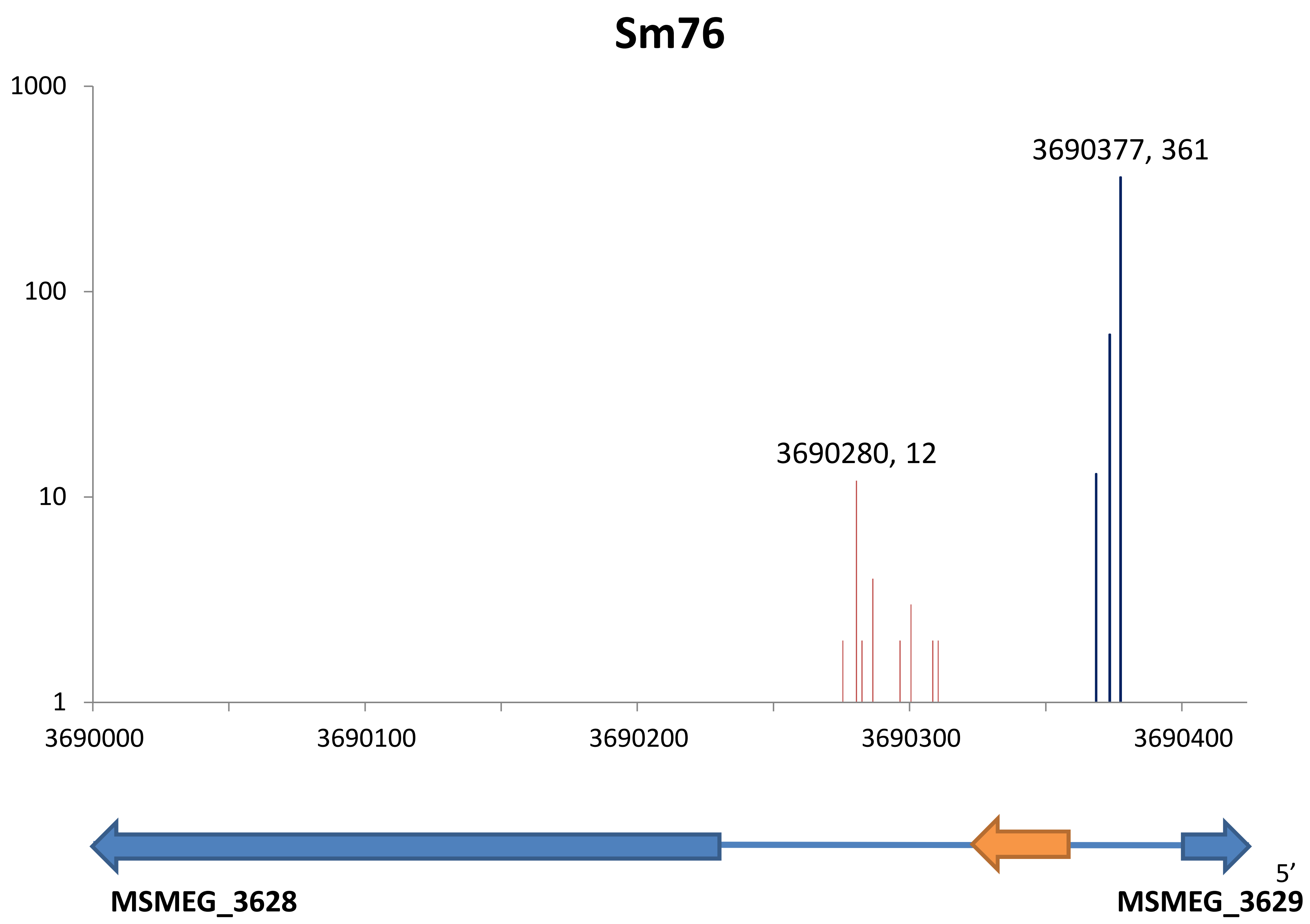

Sm76
1000
100
10
1
3690000
3690100
3690200
3690300
3690400
3690280, 12
3690377, 361
5’
MSMEG_3628
MSMEG_3629

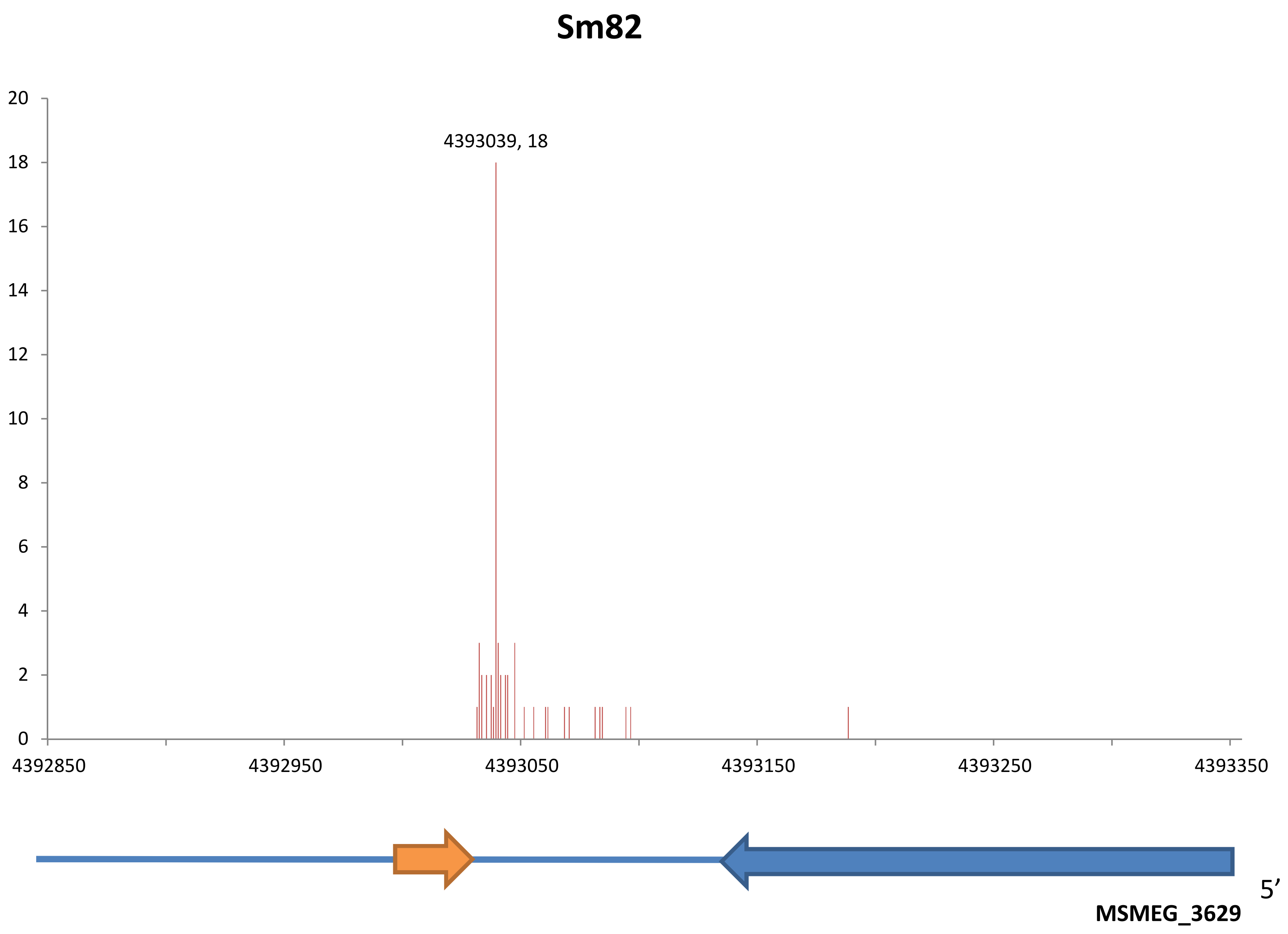

Sm82
20
18
16
14
12
10
8
6
4
2
0
4393039, 18
4392850
4392950
4393050
4393150
4393250
4393350
5'
MSMEG_3629

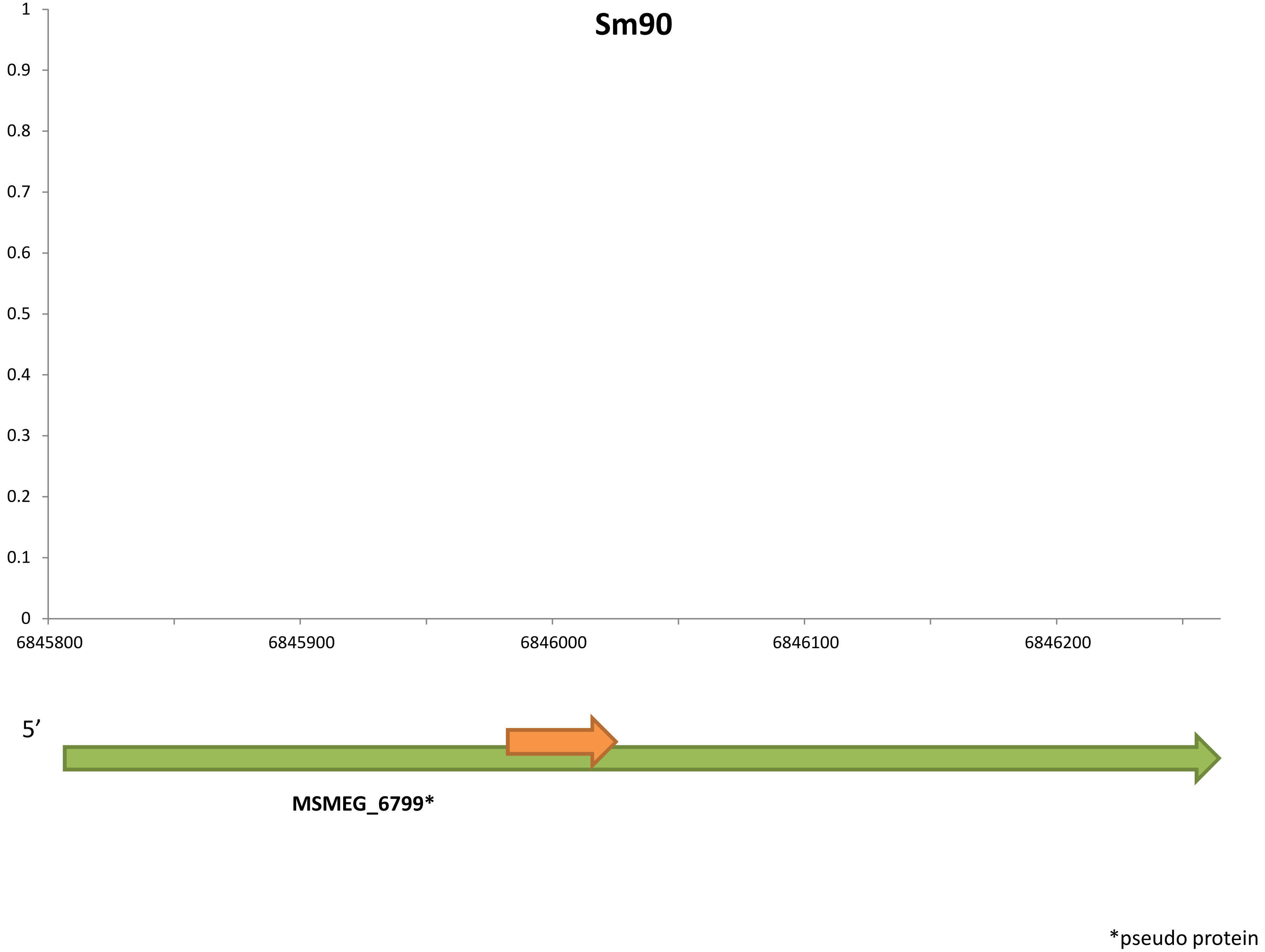
Sm90
1
0.9
0.8
0.7
0.6
0.5
0.4
0.3
0.2
0.1
0
6845800
6845900
6846000
6846100
6846200
5’
MSMEG_6799*
*pseudo protein

Sm93

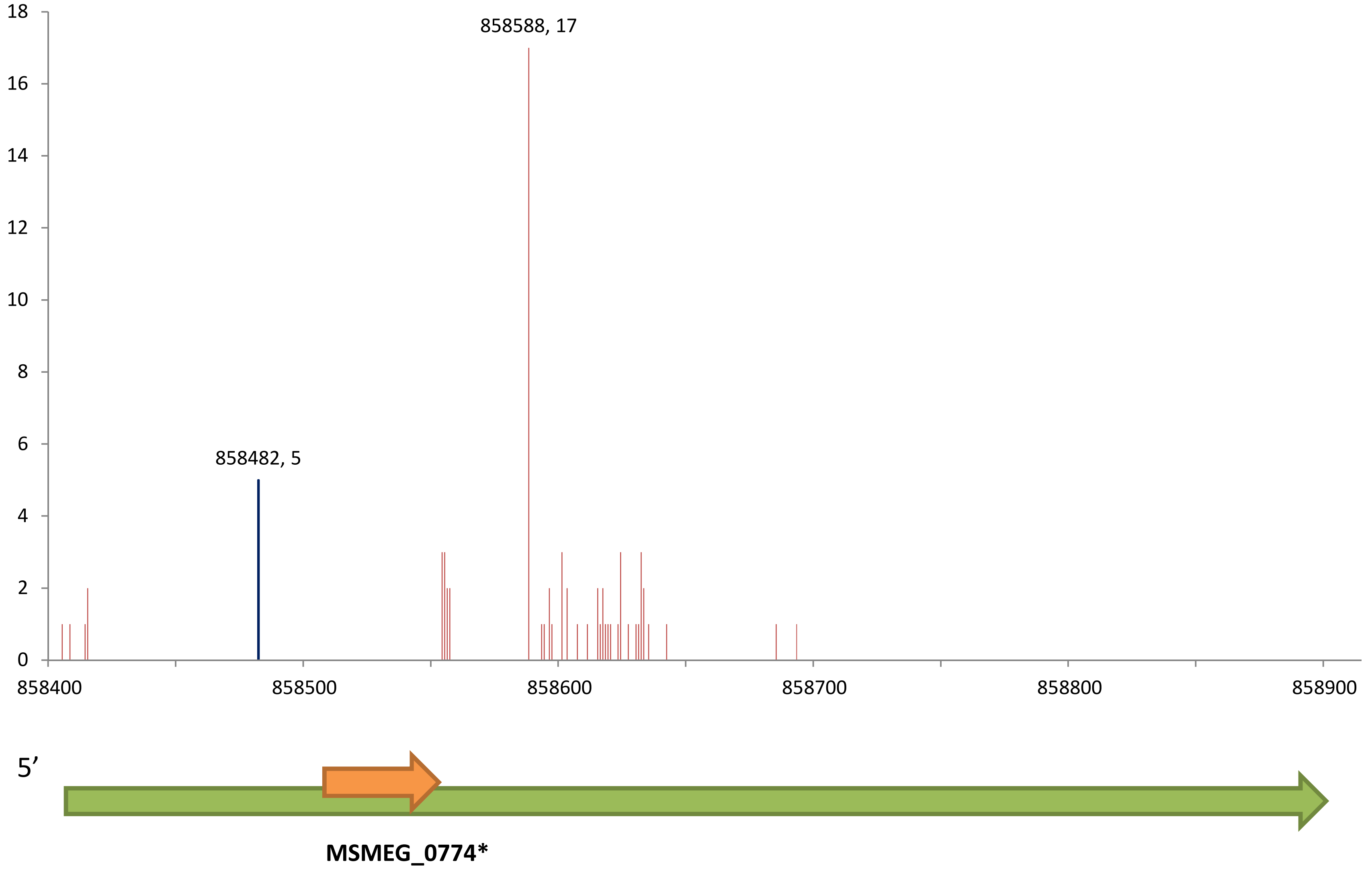

*pseudo protein

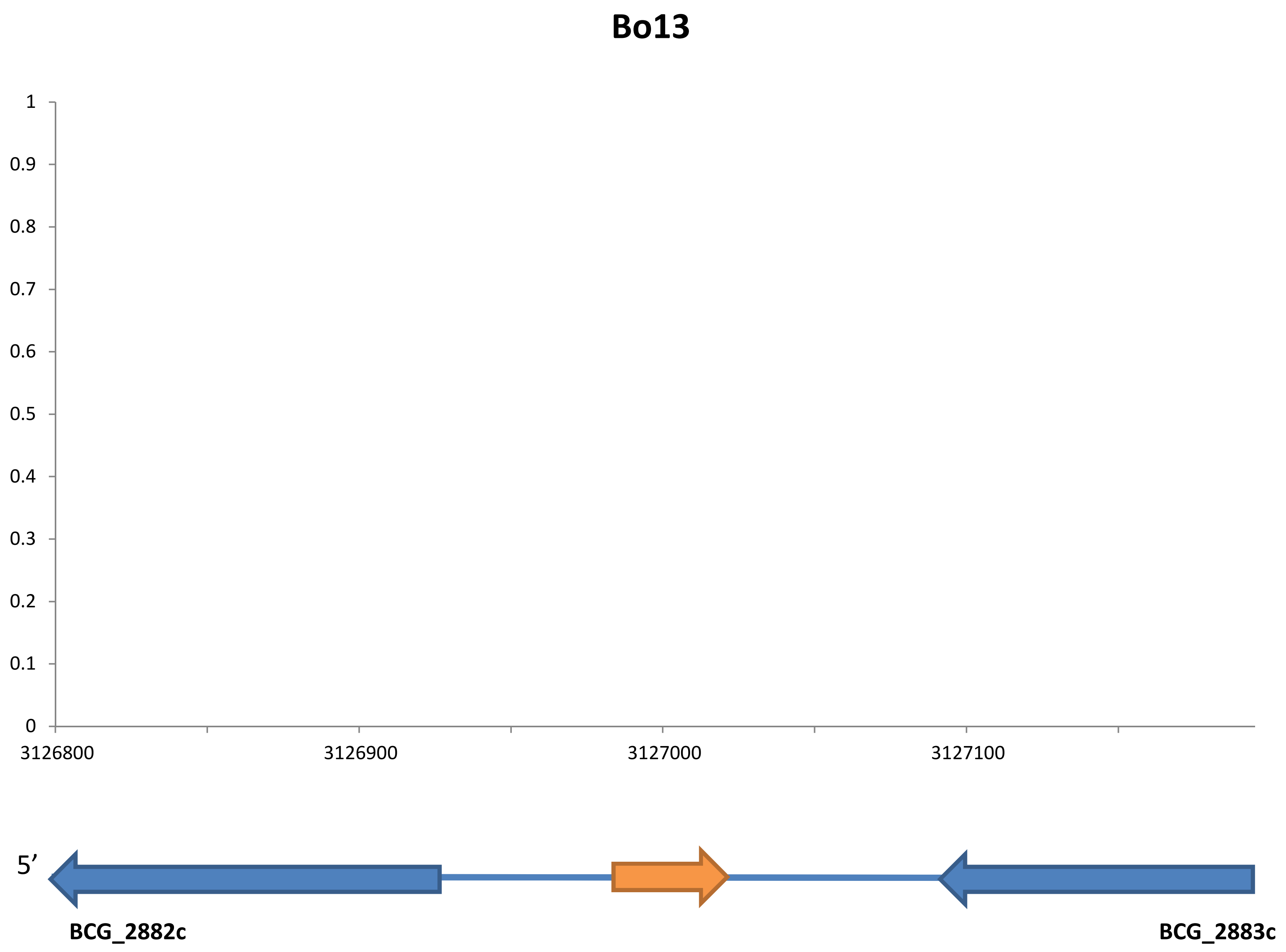

Bo13
1
0.9
0.8
0.7
0.6
0.5
0.4
0.3
0.2
0.1
0
3126800
3126900
3127000
3127100
5’
BCG_2882c
BCG_2883c

Bo27

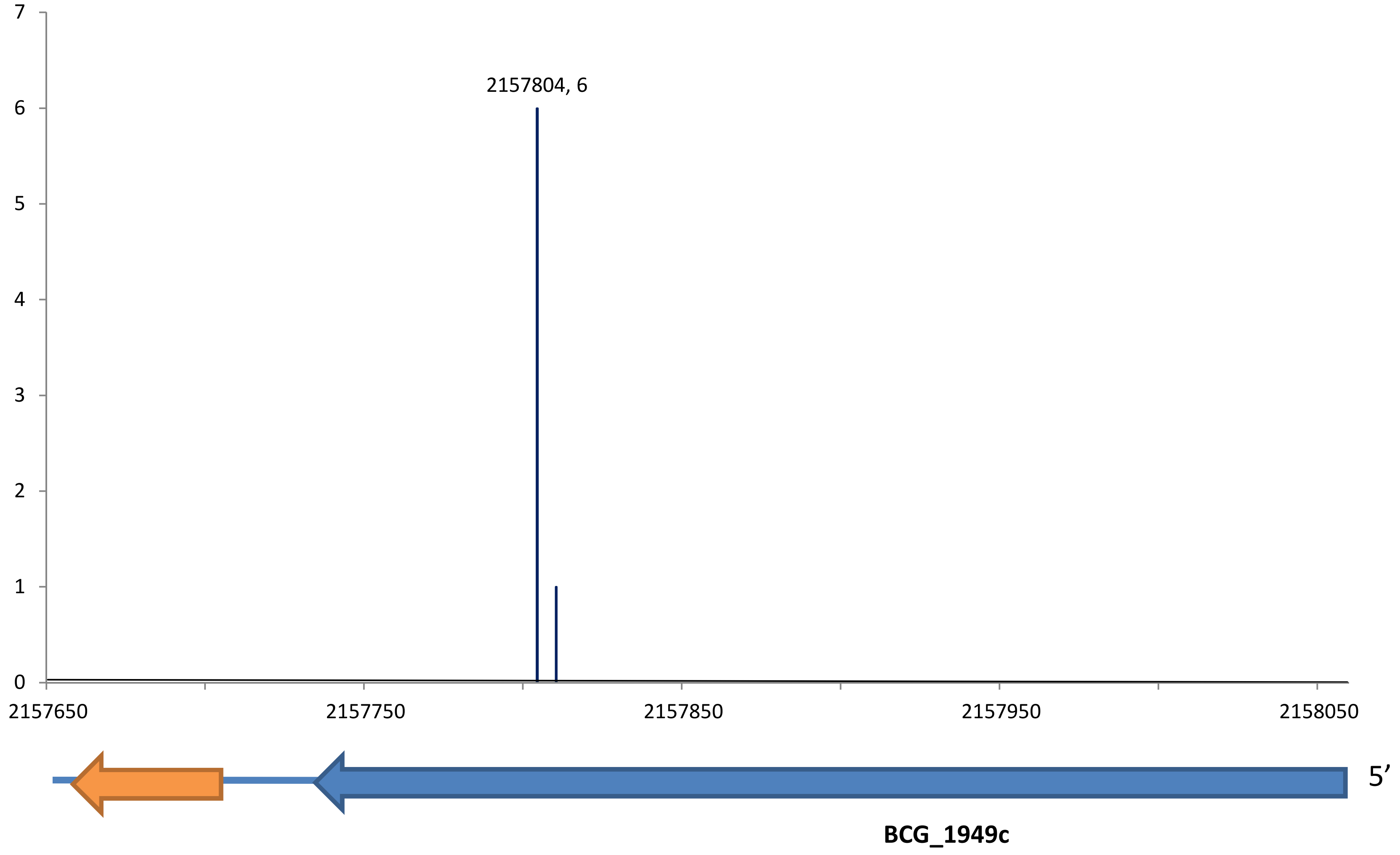

7
6
5
4
3
2
1
0
2157804, 6
2157650
2157750
2157850
2157950
2158050
5’
BCG_1949c

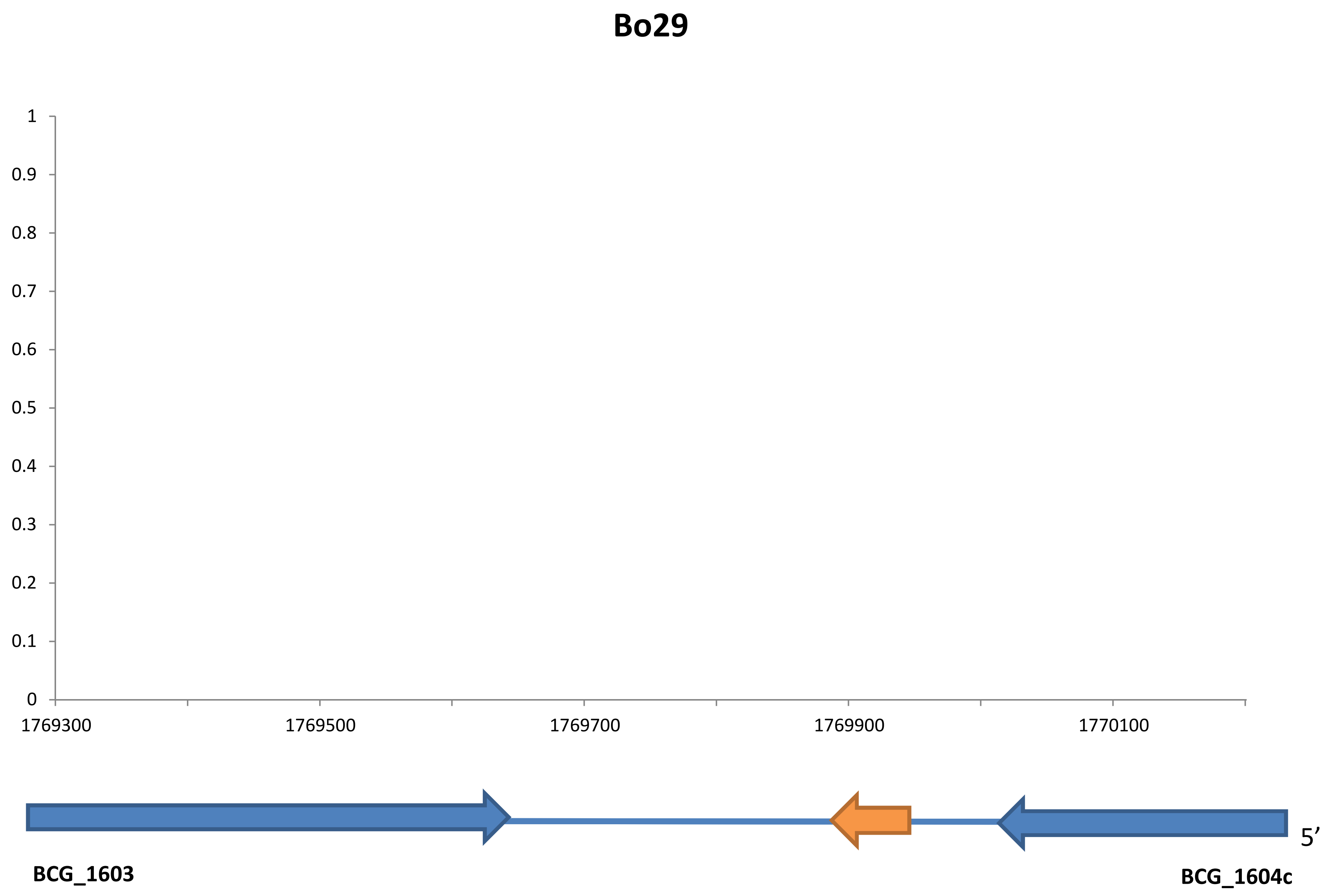

Bo29
1
0.9
0.8
0.7
0.6
0.5
0.4
0.3
0.2
0.1
0
1769300
1769500
1769700
1769900
1770100
5'
BCG_1603
BCG_1604c

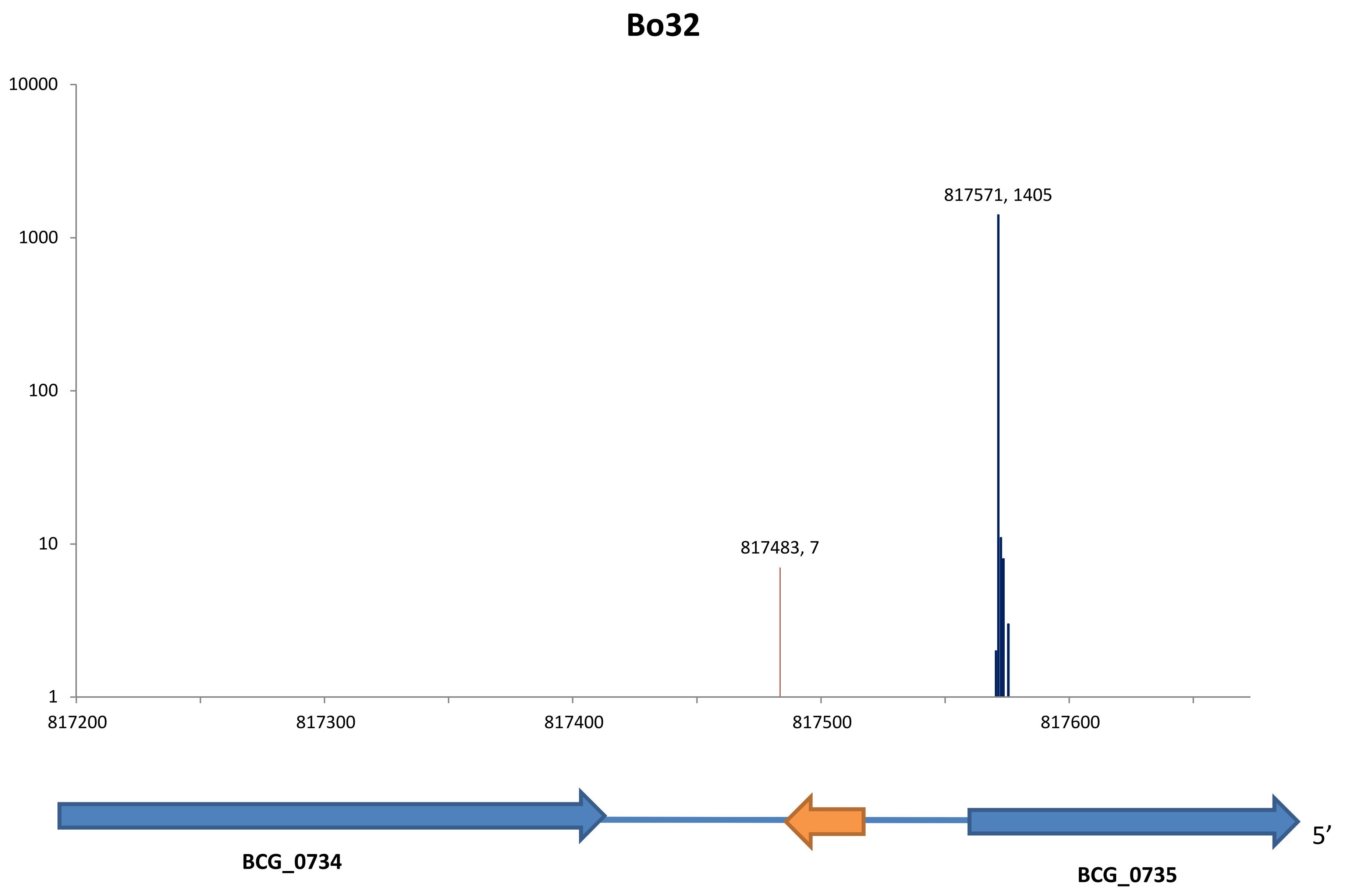
Bo32
10000
1000
100
10
1
817571, 1405
817483, 7
817200
817300
817400
817500
817600
BCG_0734
BCG_0735
5’

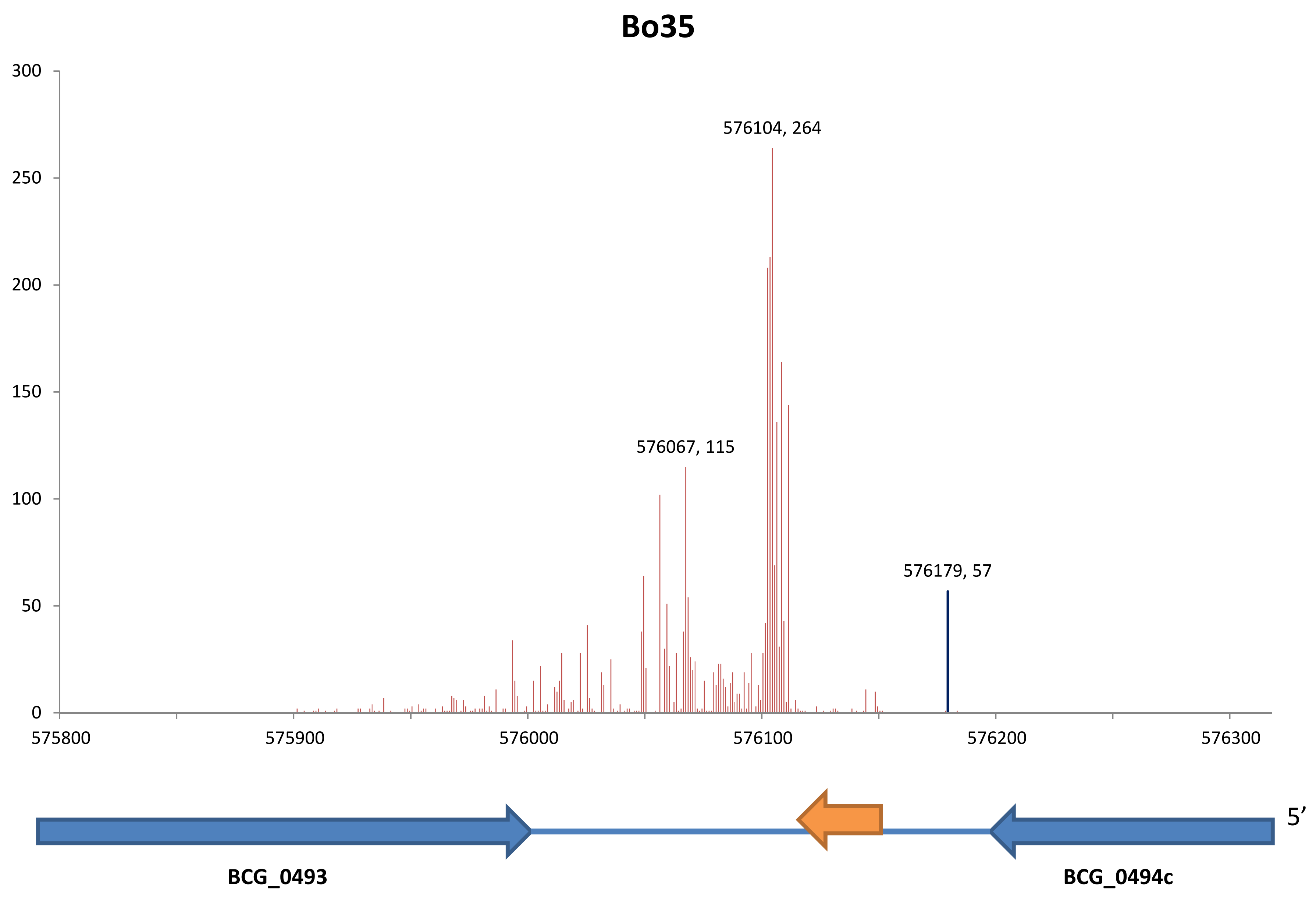
Bo35
300
250
200
150
100
50
0
576104, 264
576067, 115
576179, 57
575800
575900
576000
576100
576200
576300
5'
BCG_0493
BCG_0494c

Bo46

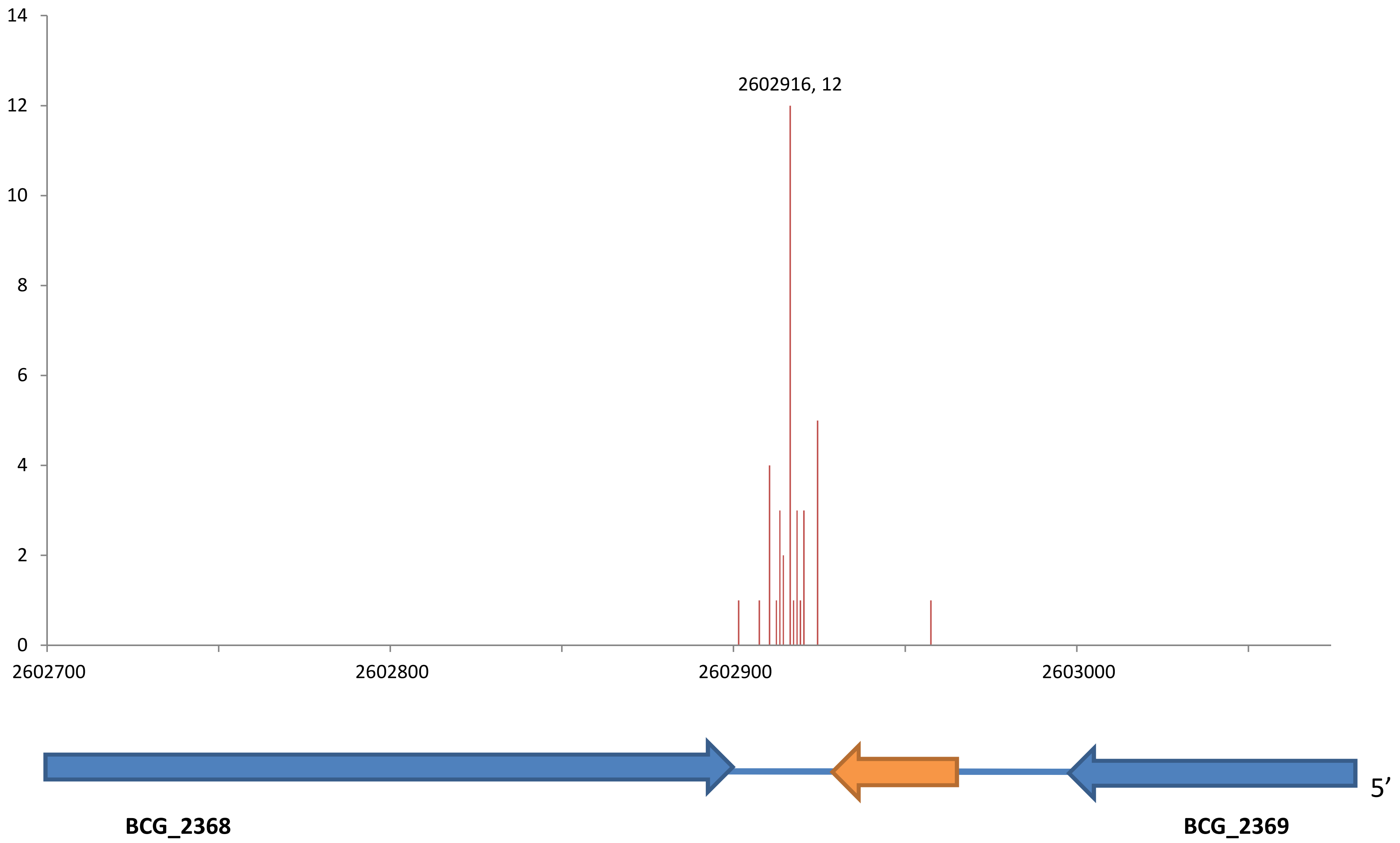

14
12
10
8
6
4
2
0
2602916, 12
2602700
2602800
2602900
2603000
5’
BCG_2368
BCG_2369

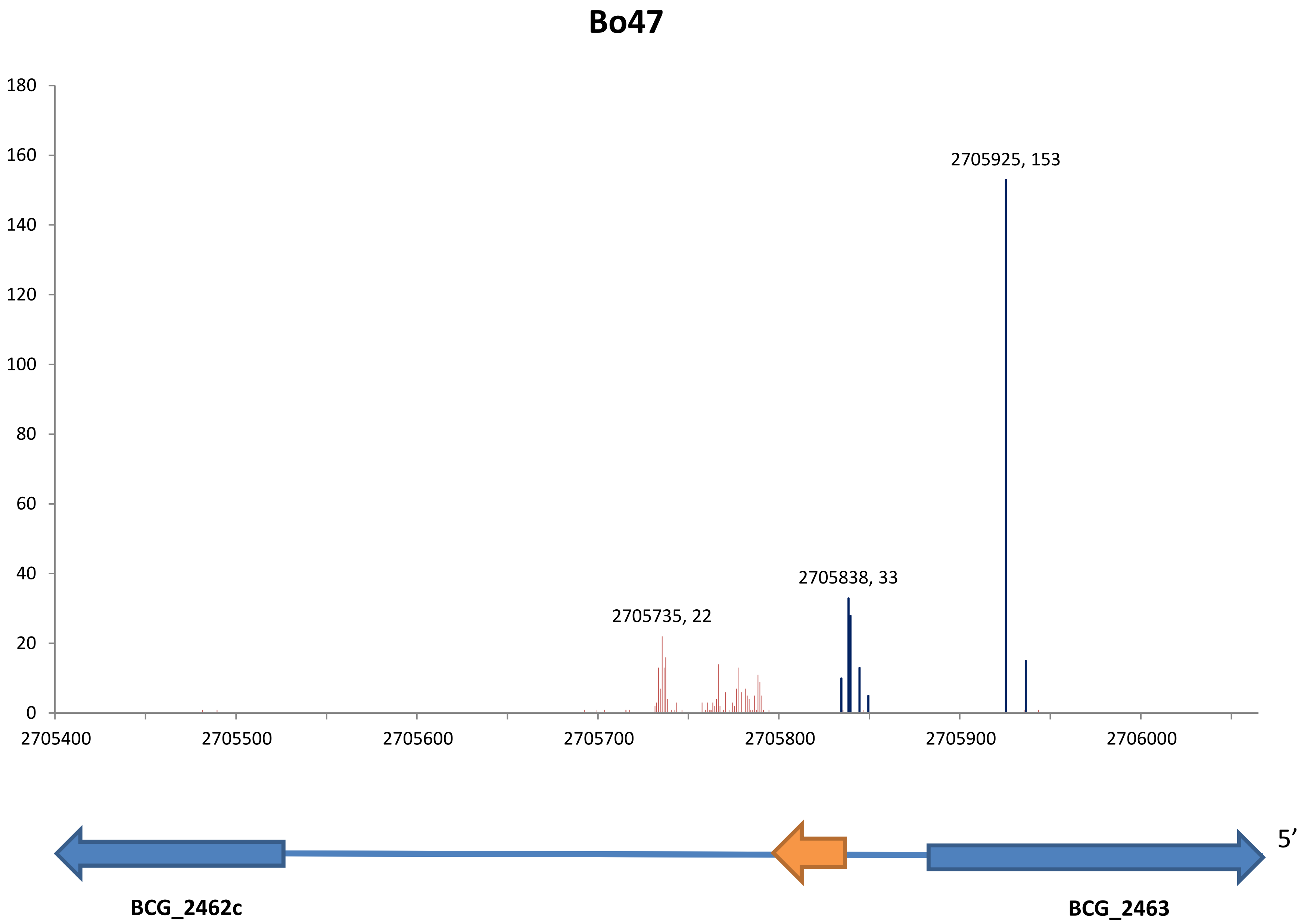

Bo47
180
160
140
120
100
80
60
40
20
0
2705925, 153
2705838, 33
2705735, 22
2705400
2705500
2705600
2705700
2705800
2705900
2706000
5'
BCG_2462c
BCG_2463

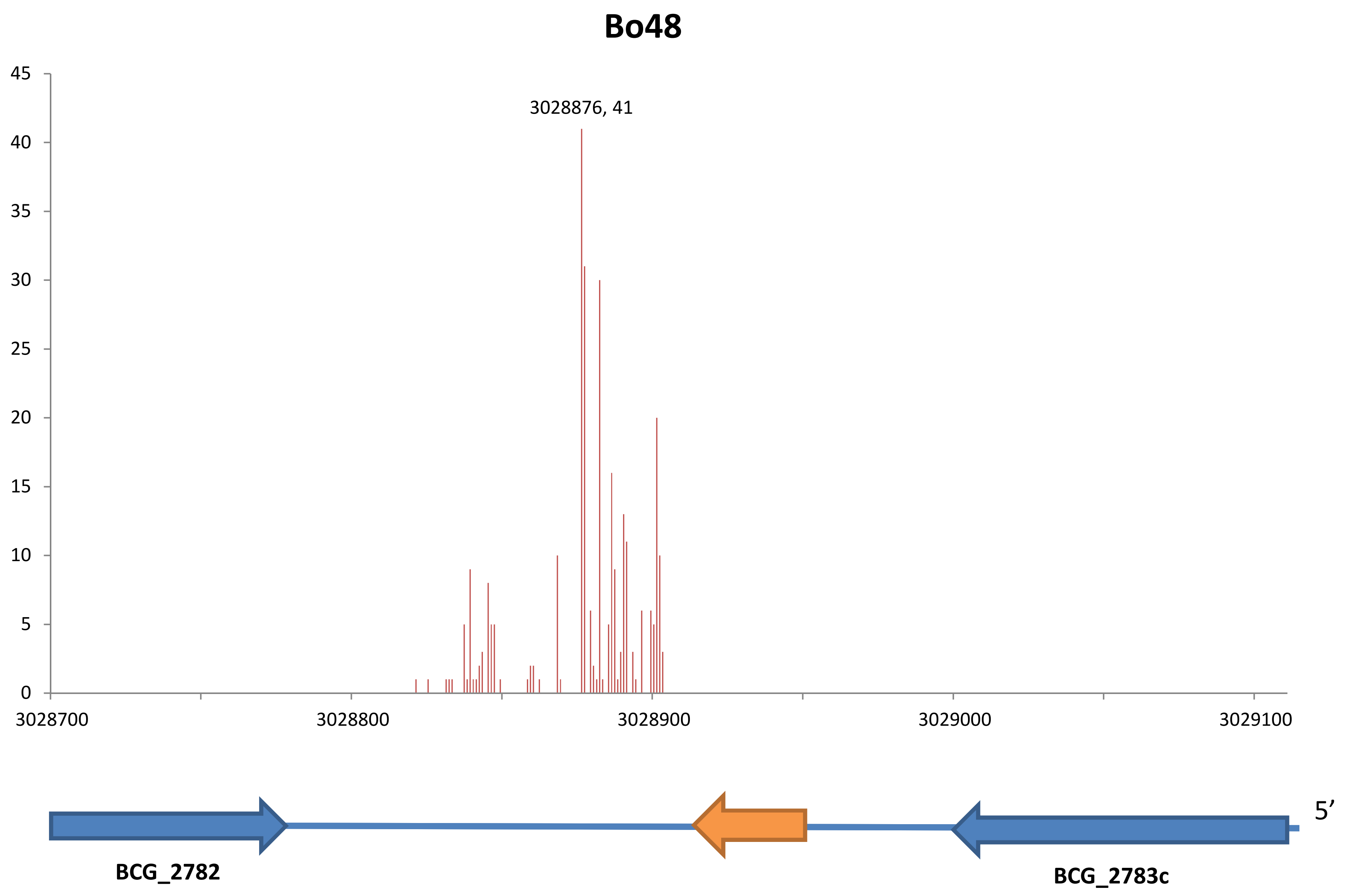
Bo48
3028876, 41
45
40
35
30
25
20
15
10
5
0
3028700
3028800
3028900
3029000
3029100
5’
BCG_2782
BCG_2783c

Bo53

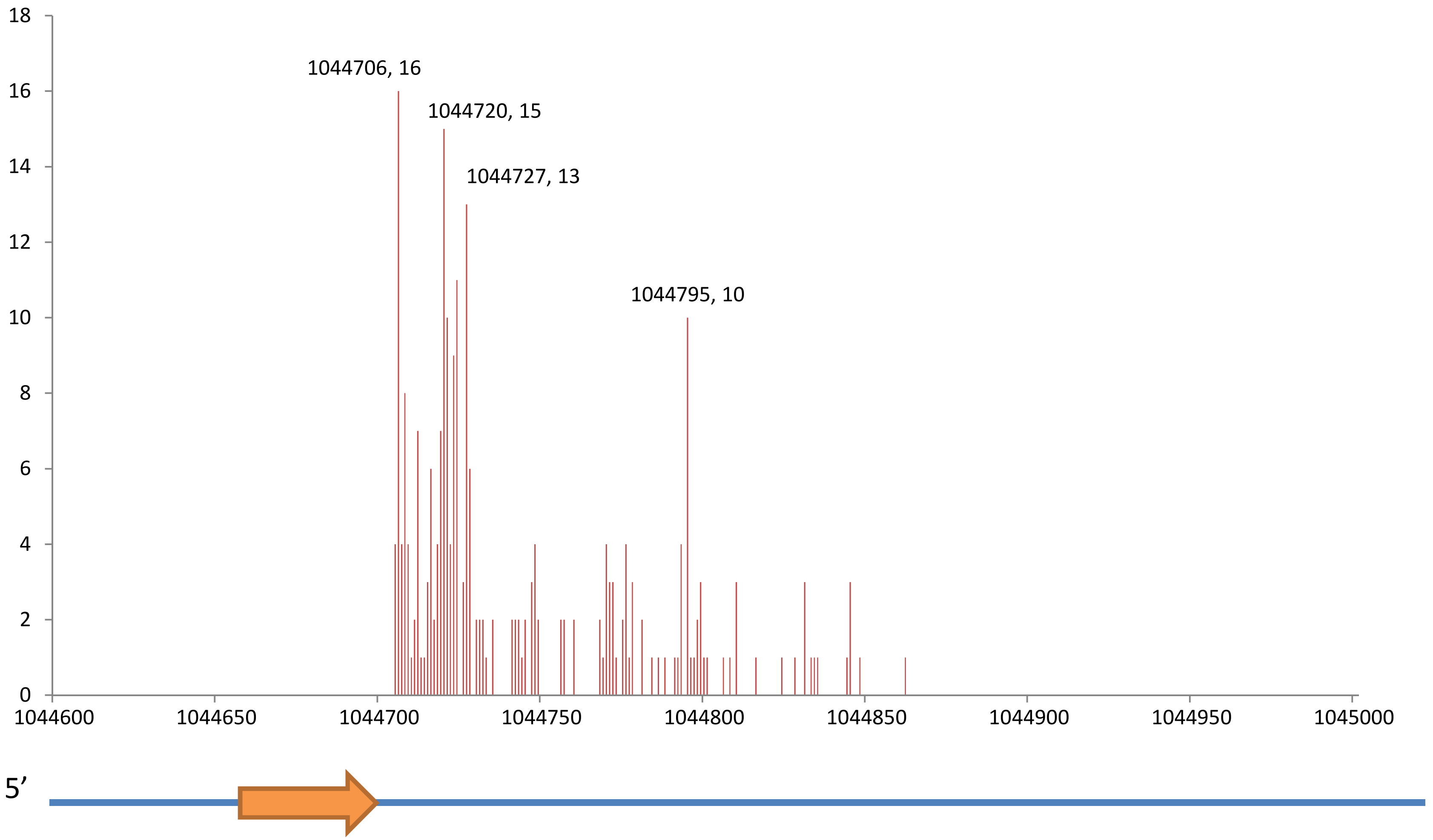

18
16
14
12
10
8
6
4
2
0
1044706, 16
1044720, 15
1044727, 13
1044795, 10
1044600
1044650
1044700
1044750
1044800
1044850
1044900
1044950
1045000
5’

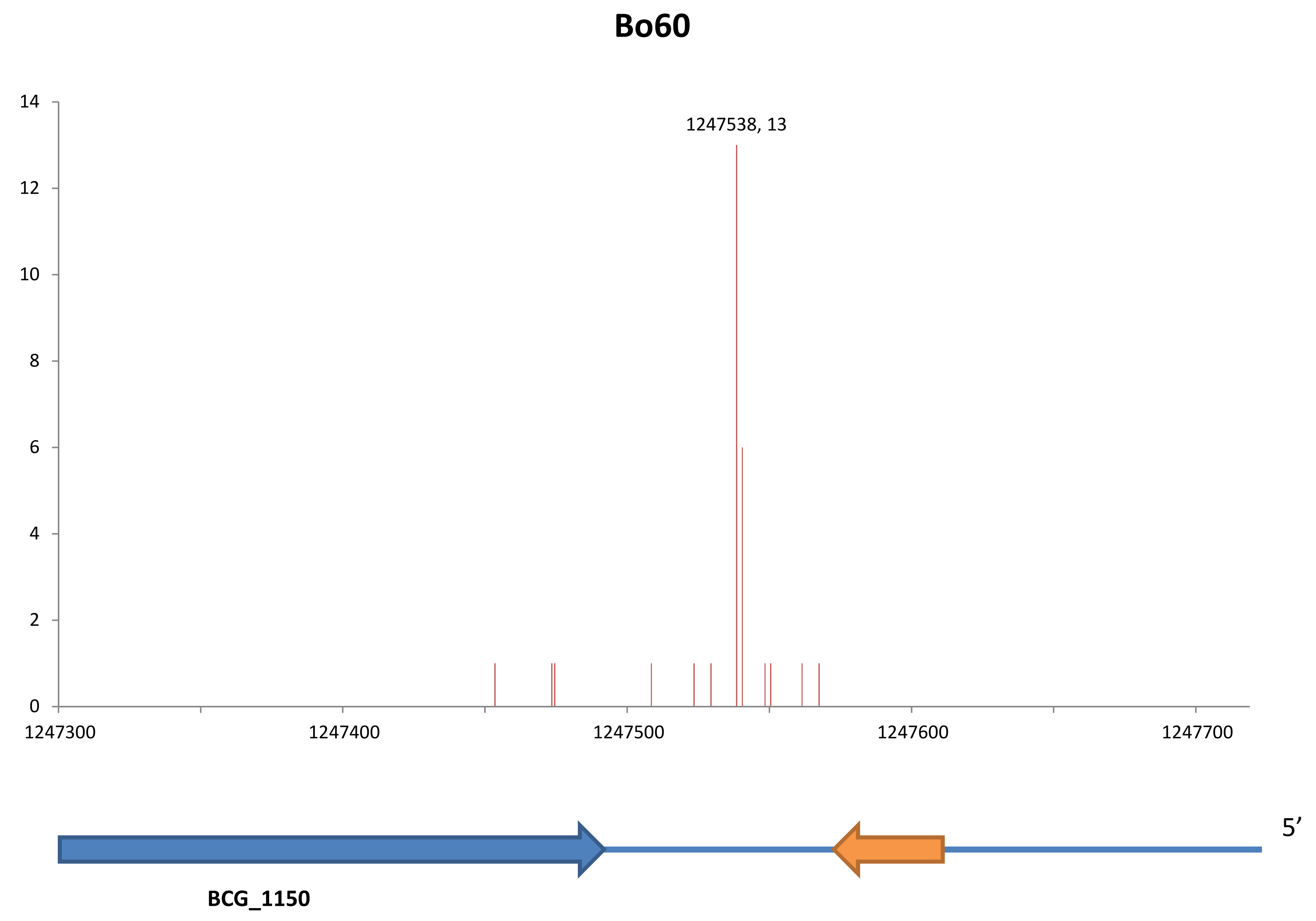

Bo60
1247538, 13
14
12
10
8
6
4
2
0
1247300
1247400
1247500
1247600
1247700
5’
BCG_1150

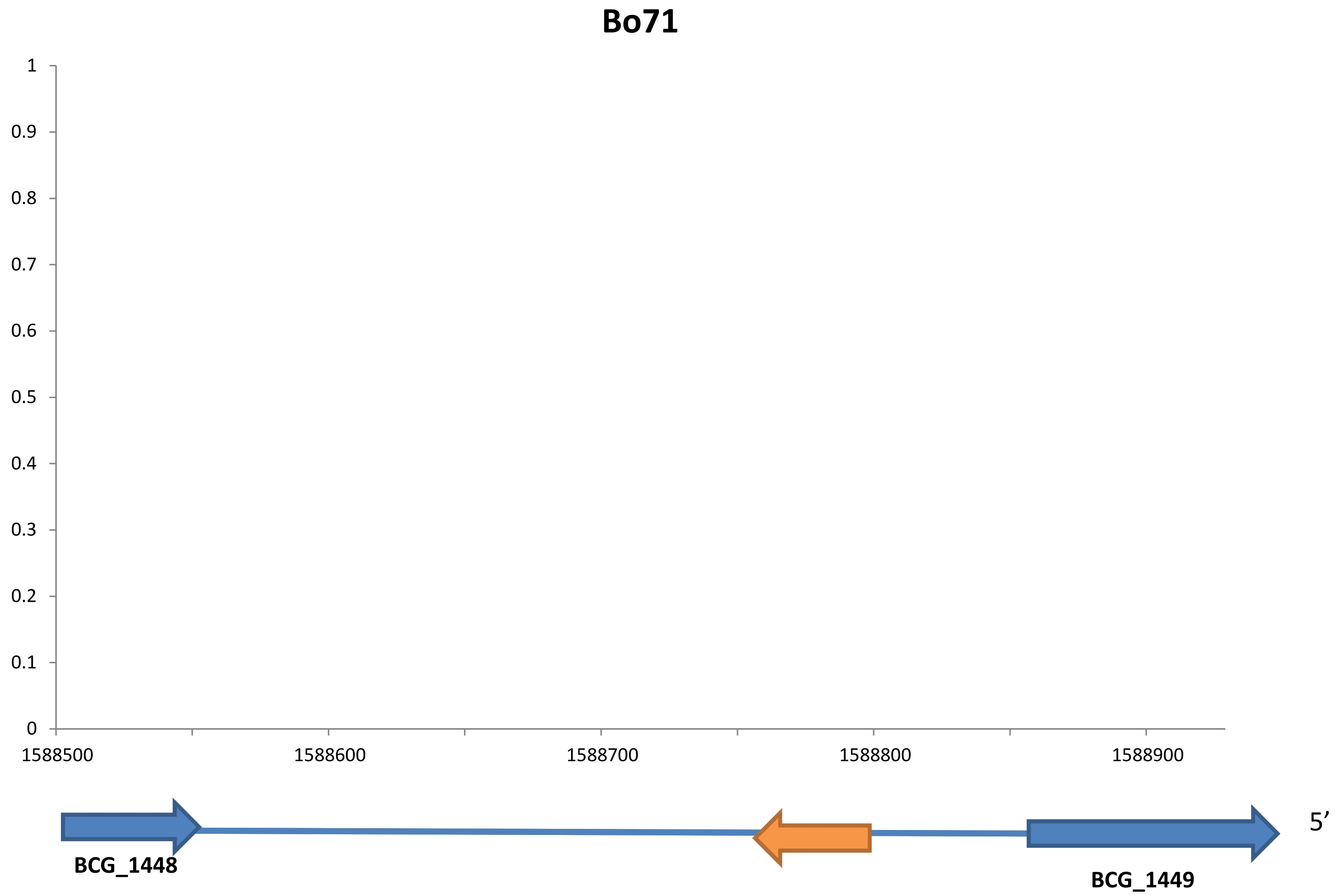
Bo71
1
0.9
0.8
0.7
0.6
0.5
0.4
0.3
0.2
0.1
0
1588500
1588600
1588700
1588800
1588900
5’
BCG_1448
BCG_1449

Bo73

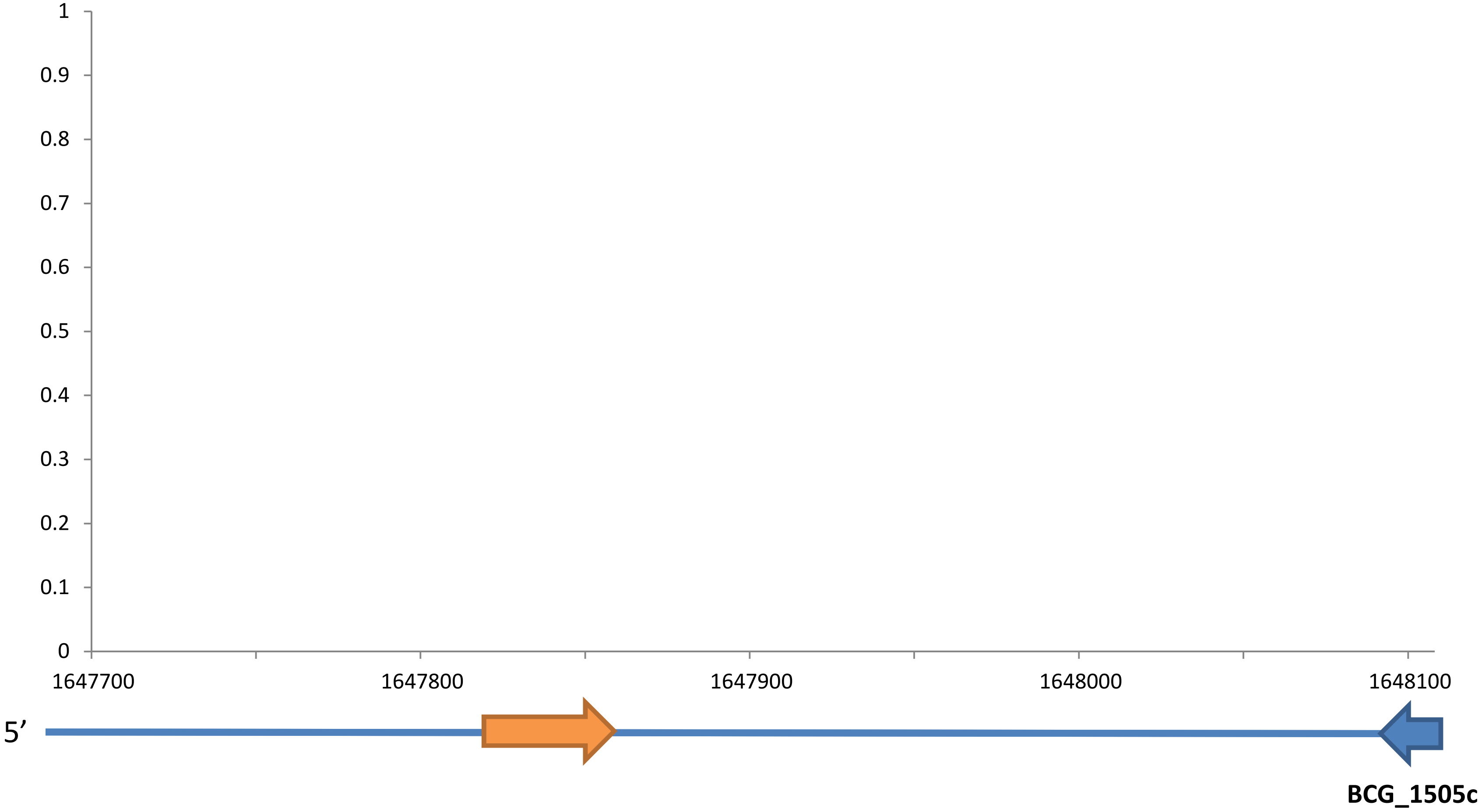

1
0.9
0.8
0.7
0.6
0.5
0.4
0.3
0.2
0.1
0
1647700
1647800
1647900
1648000
1648100
5’
BCG_1505c

Bo78

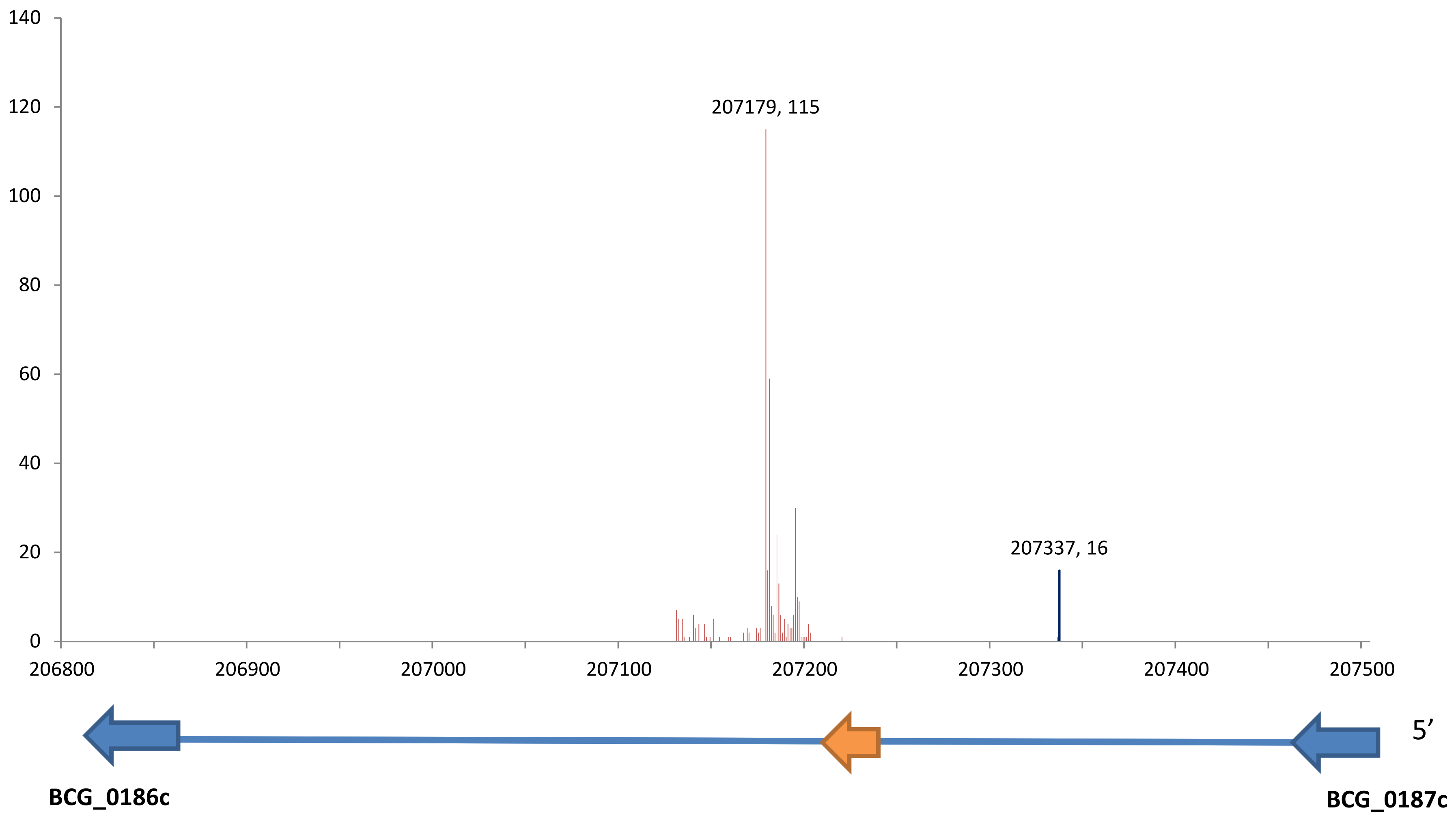
140
120
100
80
60
40
20
0
207179, 115
207337, 16
206800
206900
207000
207100
207200
207300
207400
207500
5’
BCG_0186c
BCG_0187c

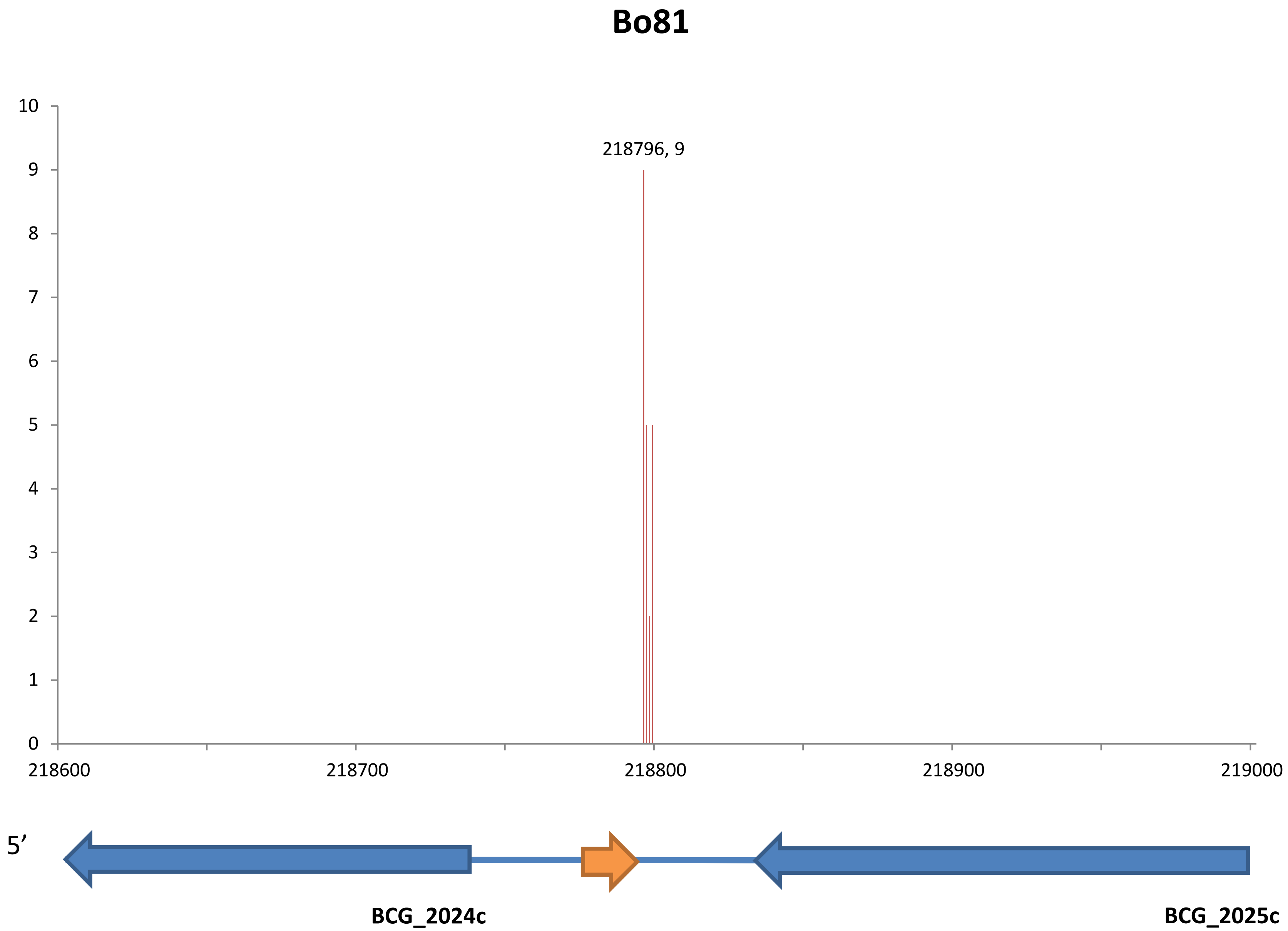

Bo81
10
9
8
7
6
5
4
3
2
1
0
218796, 9
218600
218700
218800
218900
219000
5’
BCG_2024c
BCG_2025c

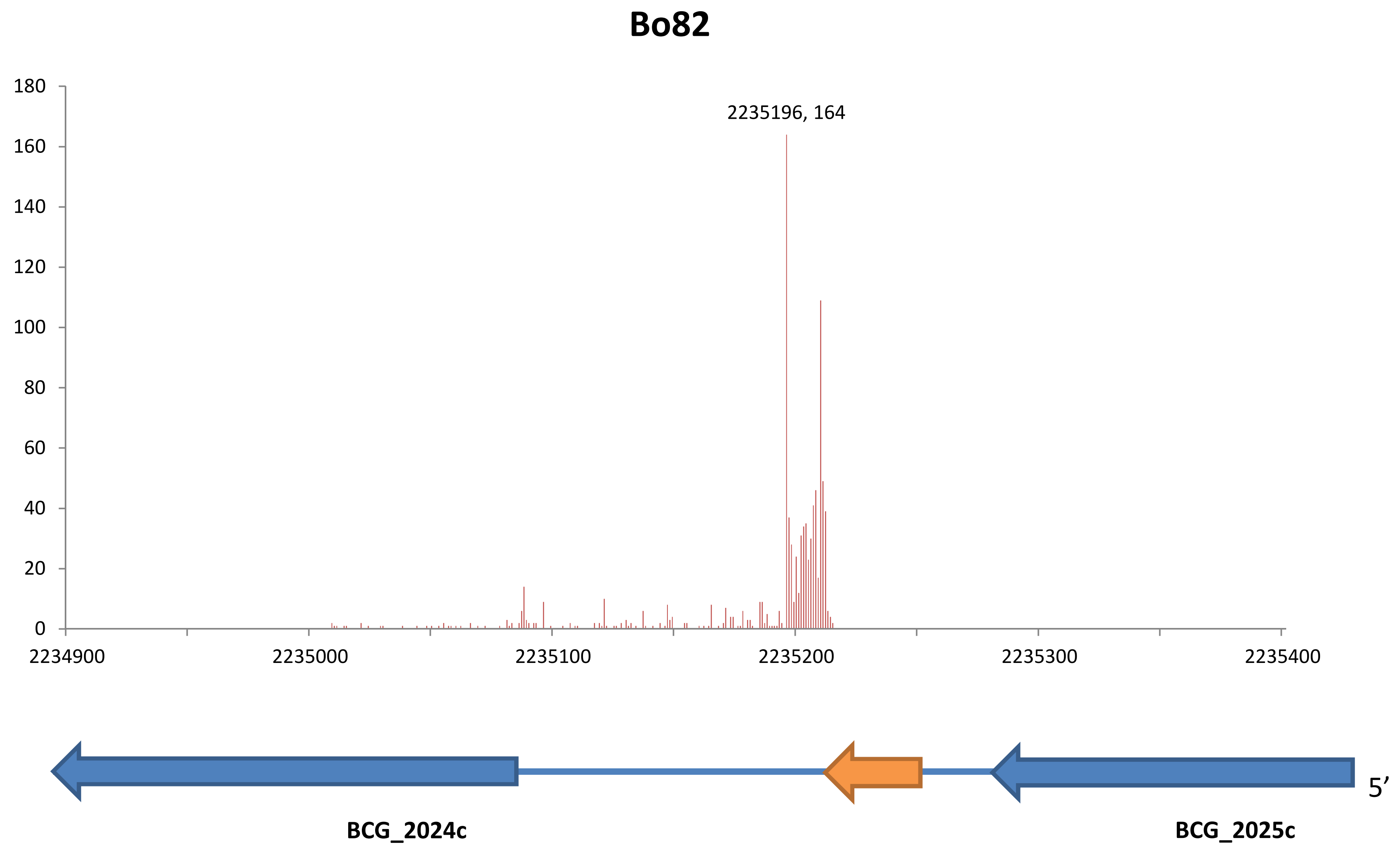

Bo82
180
160
140
120
100
80
60
40
20
0
2235196, 164
2234900
2235000
2235100
2235200
2235300
2235400
5'
BCG_2024c
BCG_2025c

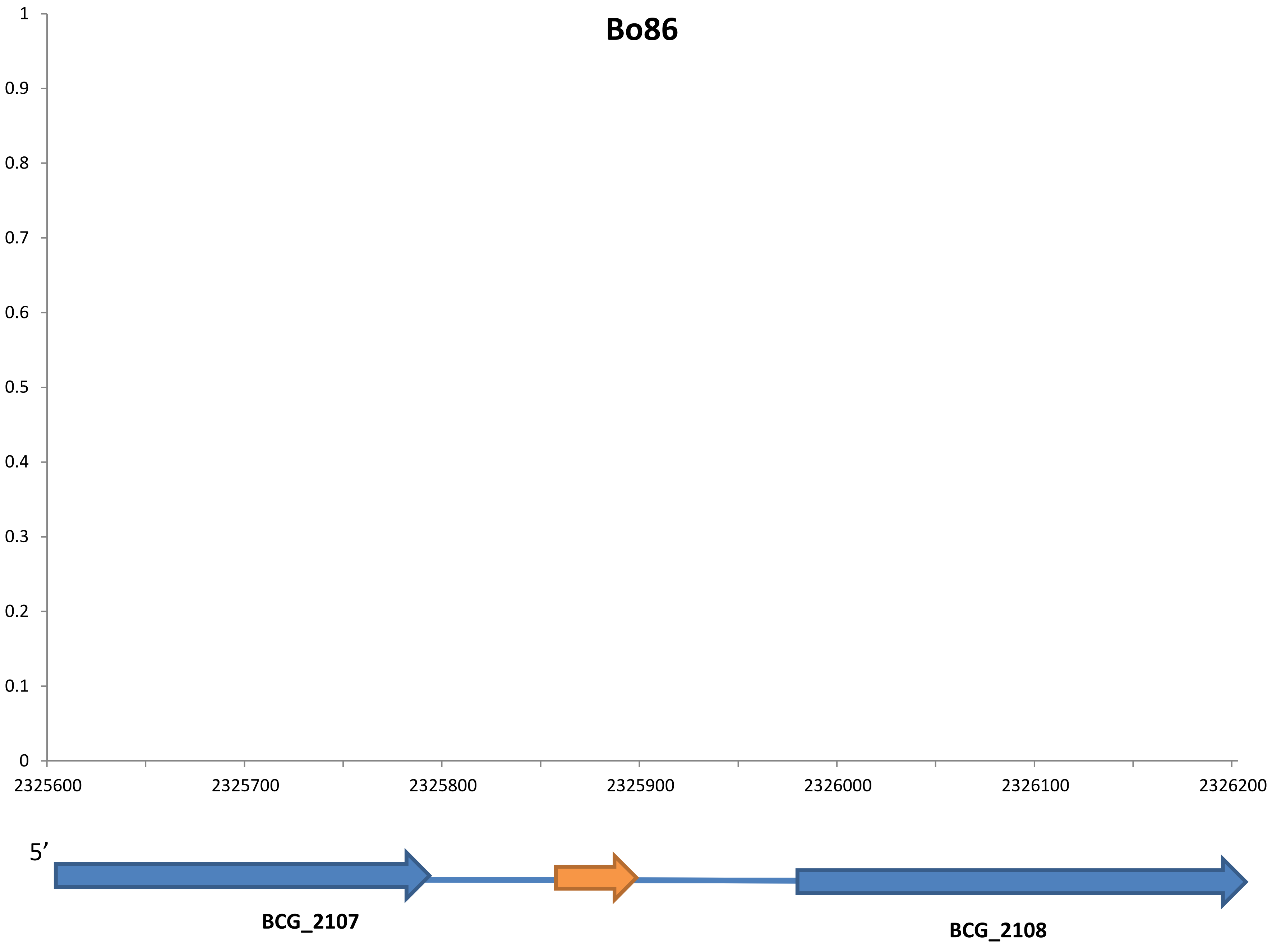

Bo86
1
0.9
0.8
0.7
0.6
0.5
0.4
0.3
0.2
0.1
0
2325600
2325700
2325800
2325900
2326000
2326100
2326200
5’
BCG_2107
BCG_2108

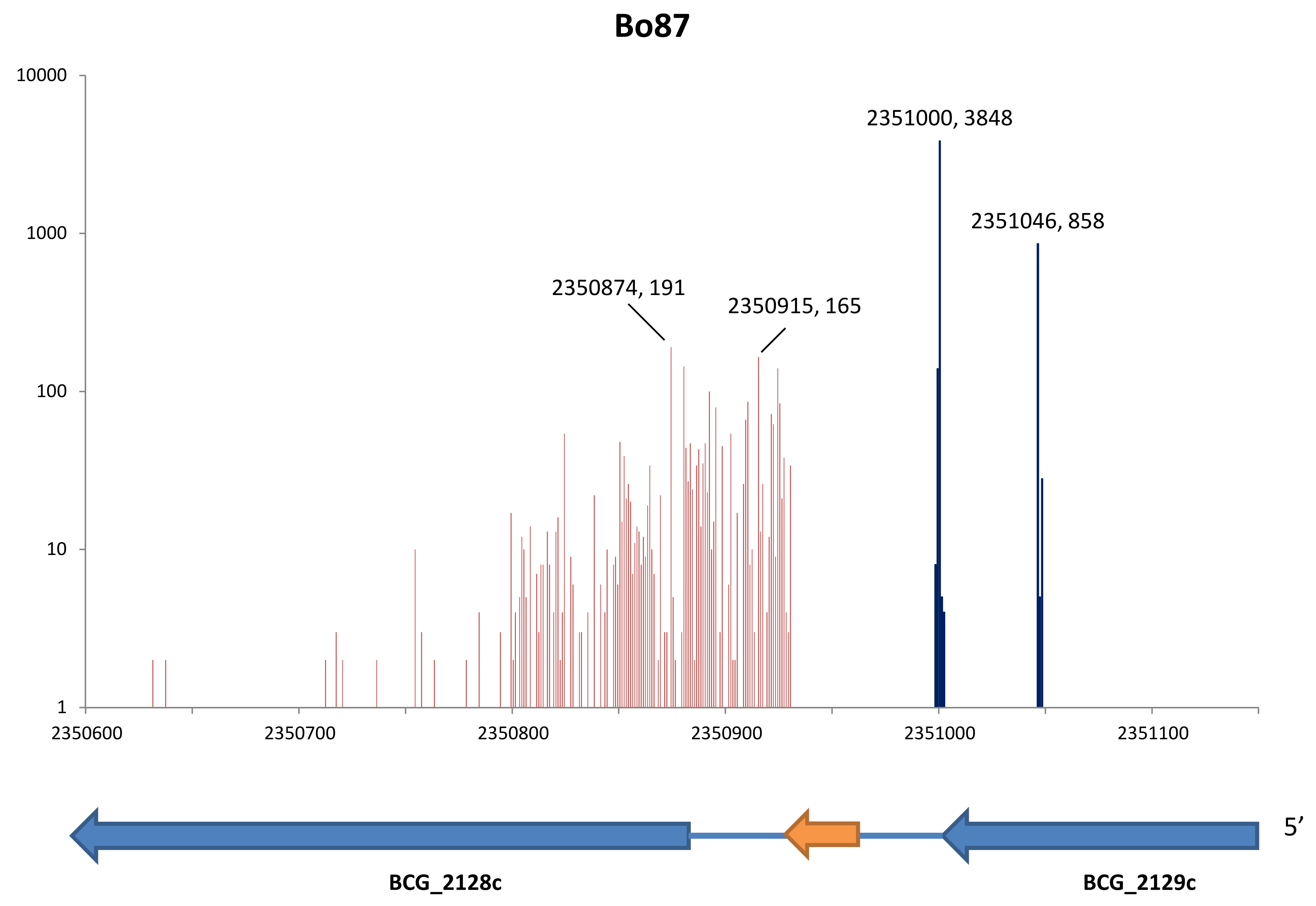

Bo87
10000
1000
100
10
1
2351000, 3848
2351046, 858
2350874, 191
2350915, 165
2350600
2350700
2350800
2350900
2351000
2351100
5’
BCG_2128c
BCG_2129c

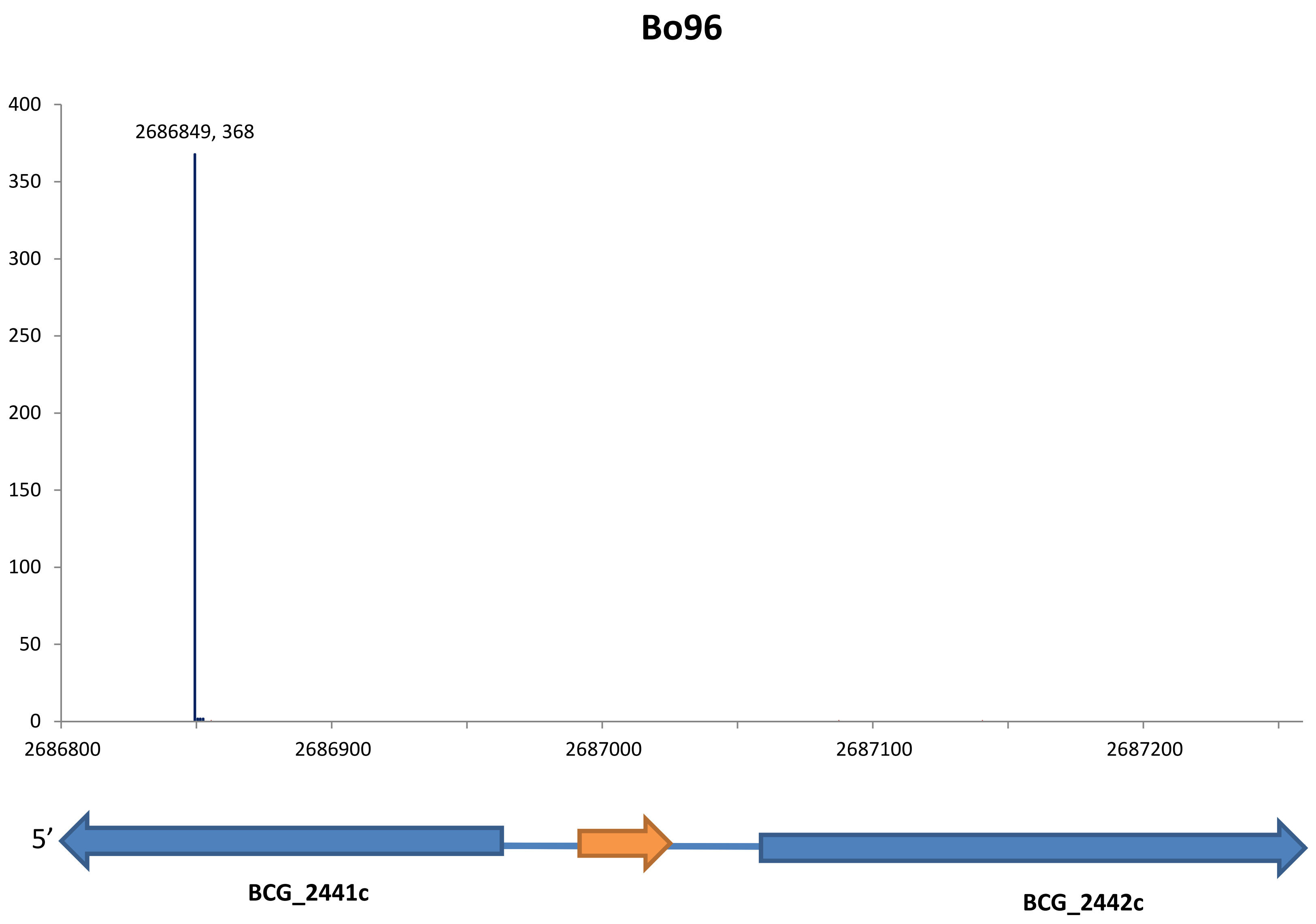
Bo96
400
350
300
250
200
150
100
50
0
2686849, 368
2686800
2686900
2687000
2687100
2687200
5’
BCG_2441c
BCG_2442c

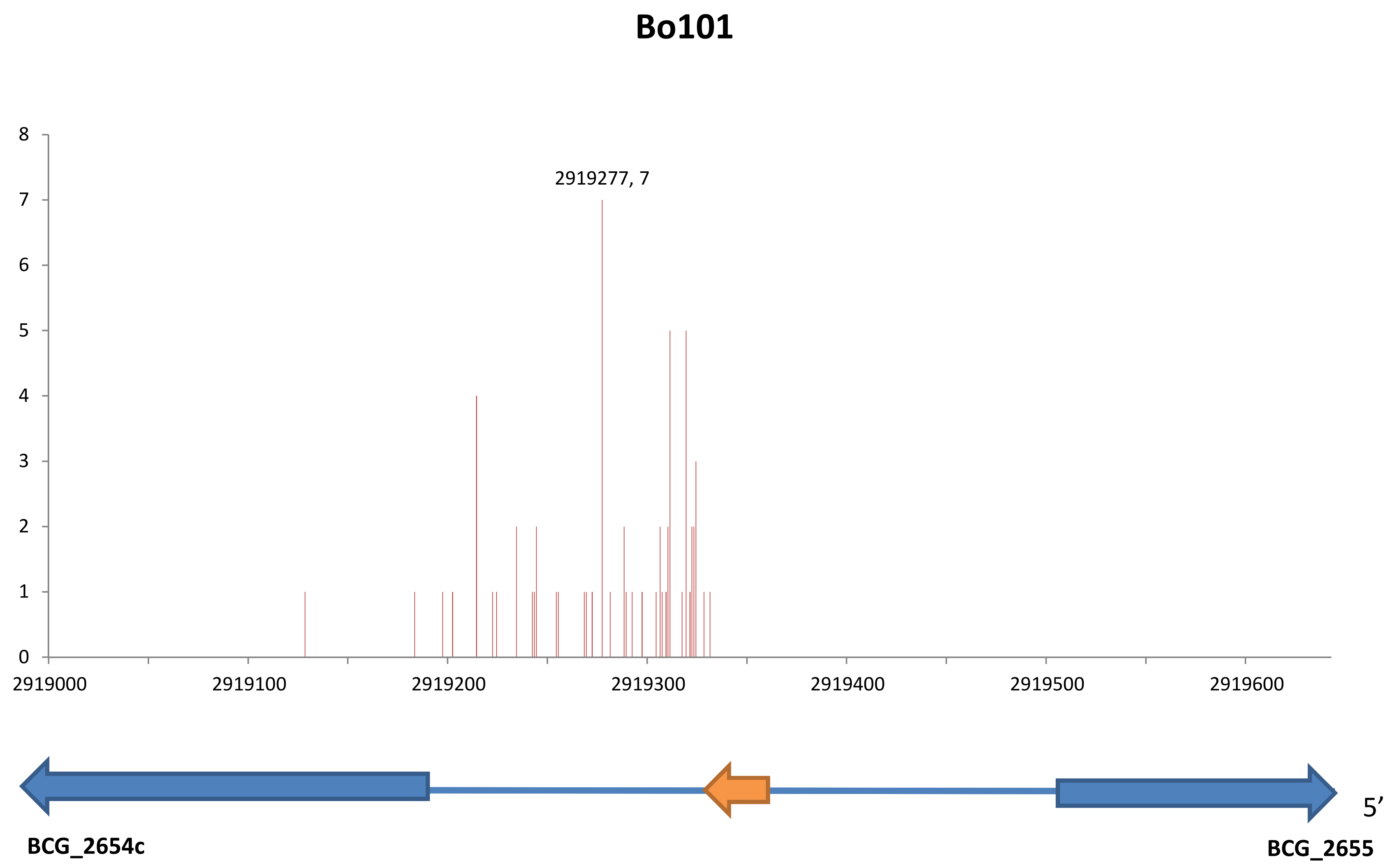

Bo101
8
7
6
5
4
3
2
1
0
2919277, 7
2919000
2919100
2919200
2919300
2919400
2919500
2919600
5’
BCG_2654c
BCG_2655

Bo105

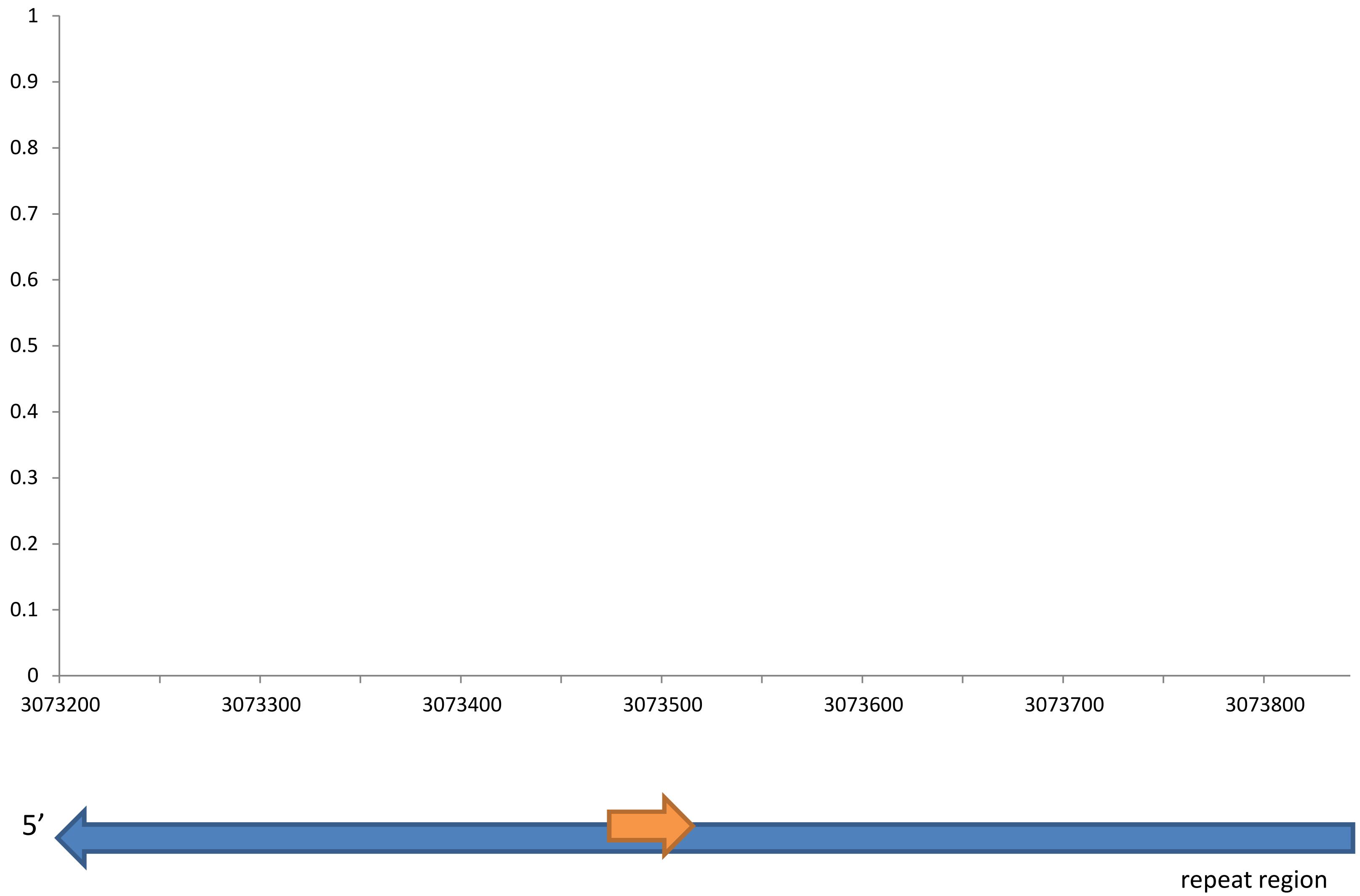
1
0.9
0.8
0.7
0.6
0.5
0.4
0.3
0.2
0.1
0
3073200
3073300
3073400
3073500
3073600
3073700
3073800
5’
repeat region

Bo118

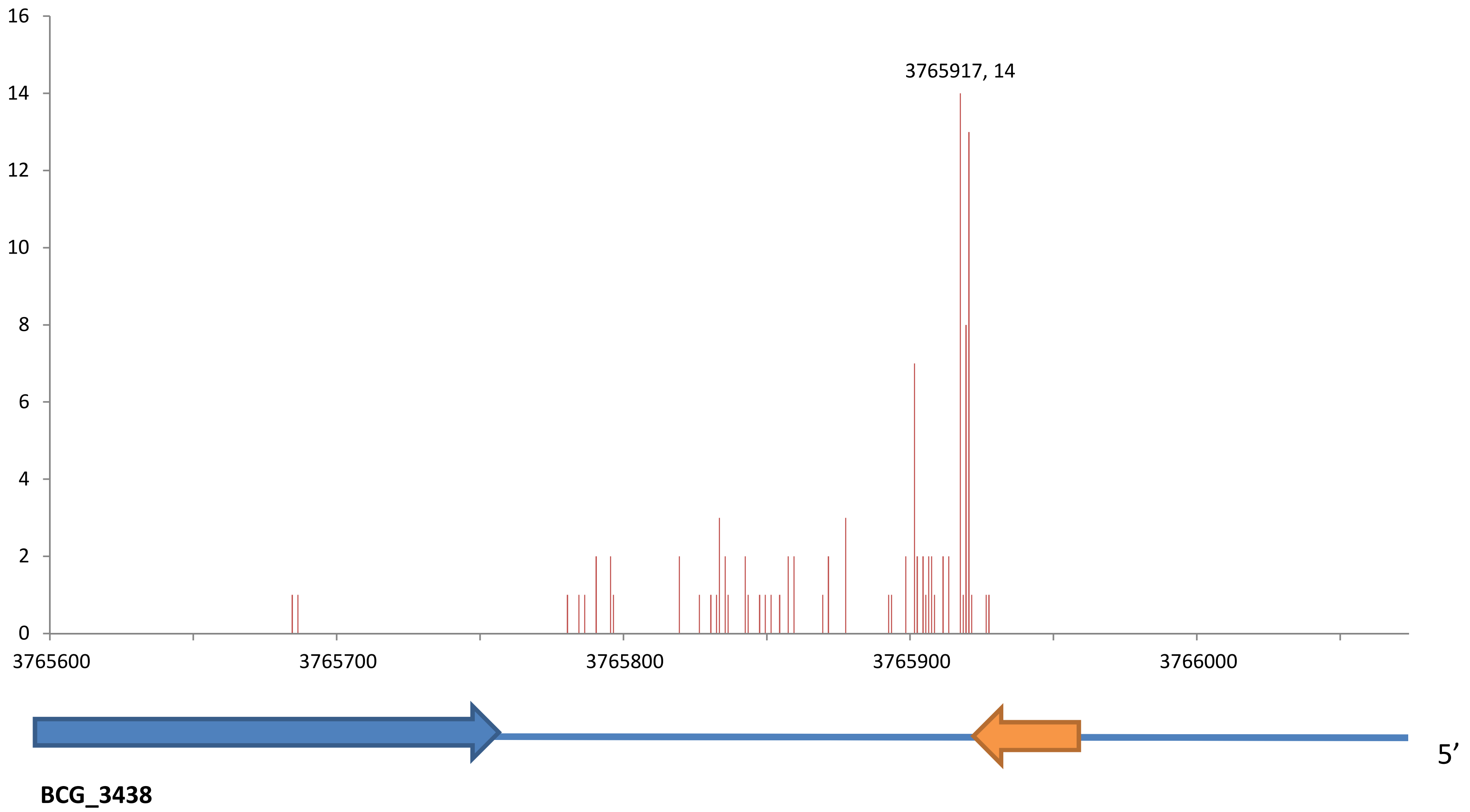
16
14
12
10
8
6
4
2
0
3765917, 14
3765600
3765700
3765800
3765900
3766000
5’
BCG_3438

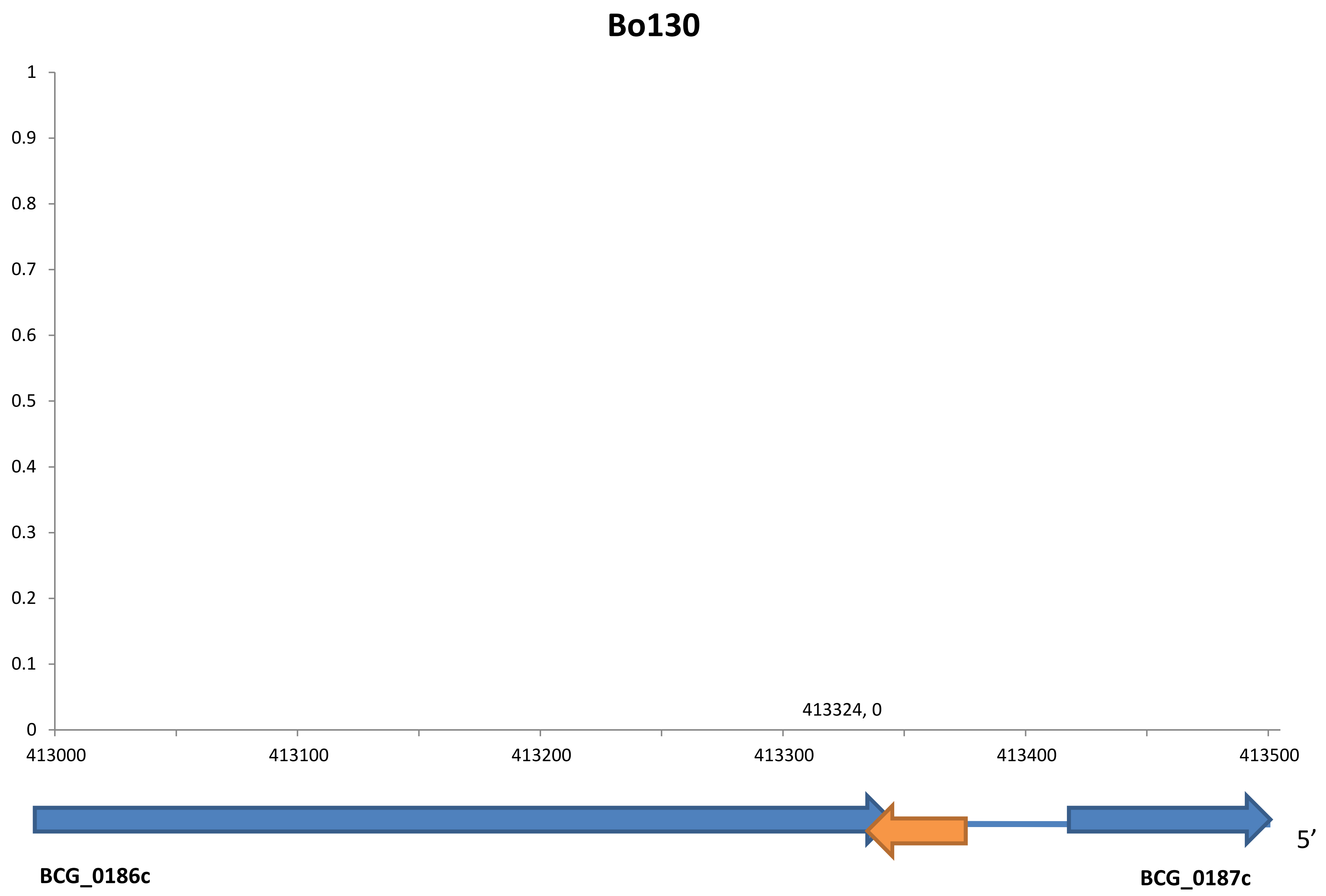

Bo130
1
0.9
0.8
0.7
0.6
0.5
0.4
0.3
0.2
0.1
0
413324, 0
413000
413100
413200
413300
413400
413500
5’
BCG_0186c
BCG_0187c

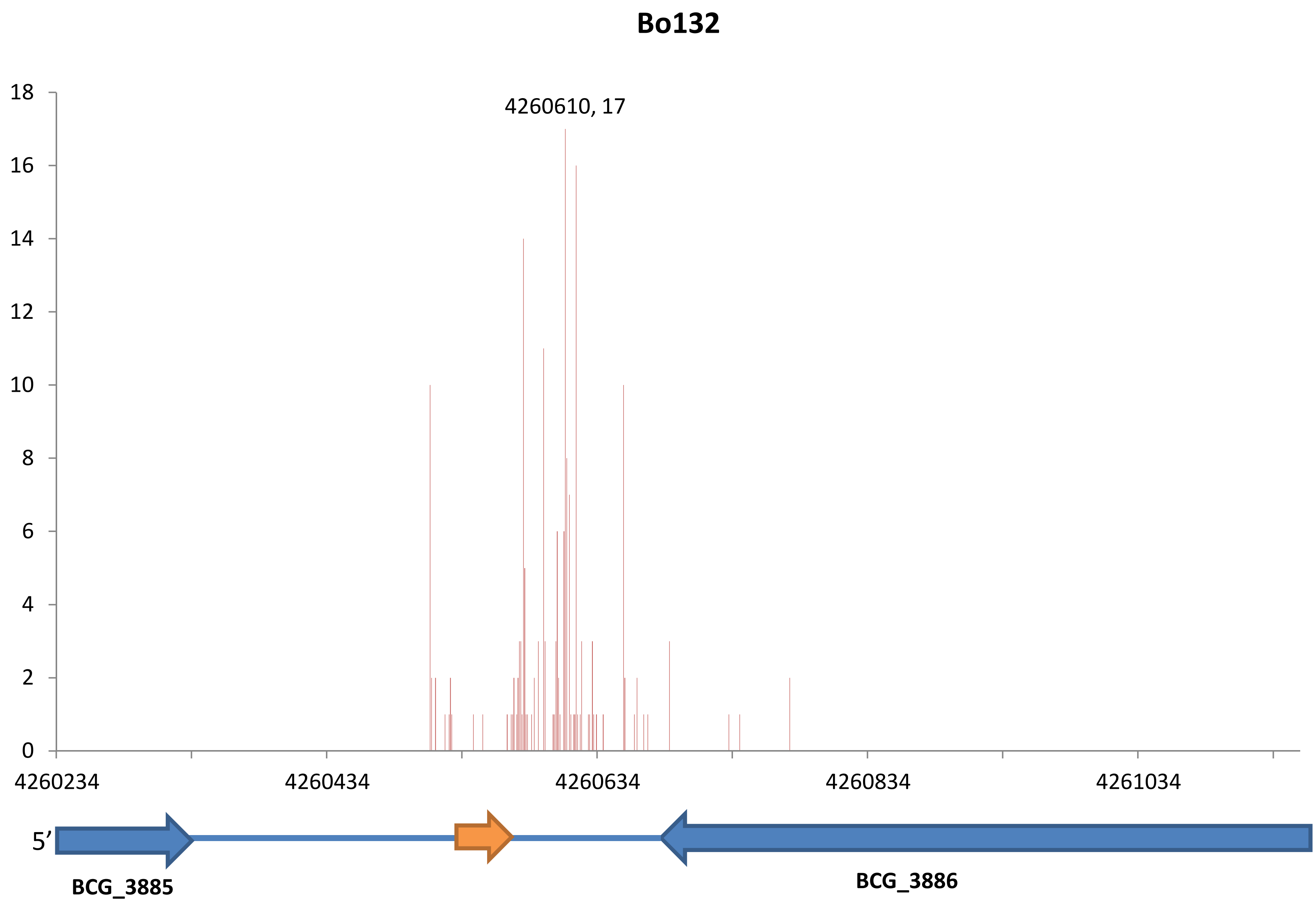
Bo132
18
16
14
12
10
8
6
4
2
0
4260610, 17
4260234
4260434
4260634
4260834
4261034
5’
BCG_3885
BCG_3886
