# Supplementary material for: Identification of Novel sRNAs in Mycobacterial Species
Source: PLoS One. 2013 Nov 14;8(11):e79411. doi: 10.1371/journal.pone.0079411 (PMC3828370; doi:10.1371/journal.pone.0079411)
Supplement: Table S1 — The oligonucleotide sequence of all probes used for Northern Blotting analysis in this study. (PDF) [file pone.0079411.s005.pdf]

Supplementary Table 1. Probes sequences used in Northern blotting analysis

sRNA candidate	Probe Sequence
Sm11	2442<br>GCTGTAGCGTTCCGGGTGCACGGGTTGCGATAGCGTCG
Sm46	SEE REF. [13]
Sm76	2547<br>GGACCGGGGGTCTCTGCAGCCCTCAGGACTCCGGCTC
Sm64	2536<br>GCGCAGGACCGGGCTGAGTAGTGCCTGCCTGCTGCG
Sm49	2474<br>GGTGGCCTGTCGGTCTCTCAGACACTACACCTAGTGGCCCC
Sm82	2553<br>GGCCGGGCGGCCGCGGCATCAGCCTGATGTCGAGG
Sm32-33	SEE REF. [13]
Sm90	2561 CGCGACGTTCGCGTCTGCCGGTTGCGGGGTGTCCCCGGG
Sm38	2465<br>CCGTCGGCCGCAGCGGCTCCCAGGGTCGCGATCGCCTCGGCG
Sm42	2469<br>CGCATGCTCGTTCTGCGGTGTCGGGTGCGGGATCGAGGTGG
Sm47	SEE REF. [13]
Sm35	SEE REF. [13]
Sm74	SEE REF. [13]
Sm19	2450<br>GGAAAAAGAGGCGGACAAAAAACAACAAACAAAAACCACC
Sm41	2468<br>GTGGCGTCGGCCCTGGCCCCGCCCAGCAGGTGCAGGCCCG
Sm93	2564<br>GGTGTGTGTCCGAGCTGCACTGCGGCAGTGCCGACGCG
Sm67	2539<br>GCAACCCCGCCGACGAGGCCCCGTGTGTCTCCGGC
Sm68	2540<br>CGGCCCCCAACAACCCGACGGTTCCTACCACCTGCGCGGC
Sm75	2546<br>GCACCCCGGCGCGCGCCCCCGATCGGTCGCCGACGG
Bo52	SEE REF. [13]
Bo80	SEE REF. [13]
Bo99	SEE REF. [13]
Bo100	SEE REF. [13]
Bo125	SEE REF. [13]
Bo15	SEE REF. [13]
Bo137	SEE REF. [13]

Bo58	SEE REF. [13]
Bo75	SEE REF. [13]
Bo41	SEE REF. [13]
Bo67	SEE REF. [13]
Bo12	SEE REF. [13]
Bo85	SEE REF. [13]
Bo117	SEE REF. [13]
Bo139	SEE REF. [13]
Bo13	2702<br>GCTCCGGCGGTTCGCGGTGCCCGCGACAGCCAGCATGTGGG
Bo32	2715<br>GCGTCTGCTCGCGAAAATGCCCAGCGTGCGGCGCTACGC
Bo35	2716<br>GGGCTGTCCCCCGAATGGTGGACAACATTTCCGGGTTCGTTG
Bo46	2720<br>GGGGATACCCGTACGCTGGCGCGTGTGGCCGTCGACCTAGGC
Bo47	2721<br>CGGGTGGTGACGTCATCCGGGTTGGACCGCTGATGGCTGCGGC
Bo48	2722<br>CGATGATGATTCAGCCGACGCCGGTCCGCGGTGCGCCCCG
Bo53	2725<br>GCAAGACCAGCCCTACCGAAGCCATCAATGGCCGCCTGG
Bo60	2728<br>CGCACACGCTTGCTTGAACATCGGGTGGAGCCGGTGG
Bo71	2732<br>CCGCGAGTGATCCCCGGCACTGCGAGTTGCGACGCCACC
Bo73	2734<br>GAAAAGTCAGCGGCCCTGACAGAGCAGCTGCGCGG
Bo78	2738<br>CGGGCTGCCCCTGGCCGGTCGCACCAAGACGCCGCATACG
Bo81	2739<br>GCTTCCCGGCGGGCGCGCTCTAGGCTCTAAGGGCCC
Bo82	2740<br>GGTTCACCCGACCGCCAGCGGGATTCACGCTCCCCCAGGC
Bo86	2742<br>GCCAACTCACCAGTTCCAGGTGATCGCGTGCACCCG
Bo87	2743<br>GCGCGTCACACGCCCGCTGTCTTTCTCTACCCTACCGG
Bo94	2756<br>GGACCGCCGTAATGGAGTTCGCGCCCGGCGCCGTCG
Bo96	2758<br>GGCGCTGGTGCGCCCGCTTATCACGCGTTGTTGGCCCACGGC
Bo101	2760<br>GGTGCCGCAGCCCGGCCAGCACGCCGTCAGAGTTTCACGGGG
Bo105	2761

	CGGGGAGCCGATCAGCGACCACCGCACCCTGTCAGTCGTC
Bo118	2768<br>CGCGTCCAGCTACCACCACCGTCAGCGGTGACACCTTCACCGG
Bo130	2771<br>GGCTCTGGGTGAGCCGCGTTCCCCGGAGCTGGCCCCGTCGGTG
Bo132	2773<br>CCAAAAGGAAGACCTCGGCGTGTCTGCCCGAGGTCC
Bo135	2775<br>CGGCGACACGTATCGCCGAGTGTGAATCCCGCGACGCCGCA<br>CCGG
Bo27	2711<br>GGCTAGCGTGACAGGCGTCTGCTAGGACCCGATC<br>GCCCCG
Bo29	2713<br>CGGCTGCCGCGAAATCCGGCTTTCT<br>ACCACGACGGATCC
Bo56	2726<br>CCACAGCGATCGCGGCCAGTGGCAATGCGAACCTCACCG
