# Supplementary material for: Identification of Novel sRNAs in Mycobacterial Species
Source: PLoS One. 2013 Nov 14;8(11):e79411. doi: 10.1371/journal.pone.0079411 (PMC3828370; doi:10.1371/journal.pone.0079411)
Supplement: Table S2 — The oligonucleotide sequence of all primers used for Deep-RACE PCR. (PDF) [file pone.0079411.s006.pdf]

Supplementary Table 2

Primers for 5' Deep-RACE PCR

Sm11	CCTCTCTATGGGCAGTCGGTGATTGTAGCGTTCCGGGTGC
Sm76	CCTCTCTATGGGCAGTCGGTGATGGGTCTCTGCAGCCCTC
Sm64	CCTCTCTATGGGCAGTCGGTGATGCAGGACCGGGCTGAGTA
Sm49	CCTCTCTATGGGCAGTCGGTGATGGCCTGTCGGTCTCTCAGAC
Sm82	CCTCTCTATGGGCAGTCGGTGATGCGGCATCAGCCTGATGTC
Sm90	CCTCTCTATGGGCAGTCGGTGATCGACGTTCGCGTCTGCC
Sm38	CCTCTCTATGGGCAGTCGGTGATTCGCGATCGCCTCGGC
Sm42	CCTCTCTATGGGCAGTCGGTGATATGCTCGTTCTGCGGTGTC
Sm19	CCTCTCTATGGGCAGTCGGTGATGGAAAAAGAGGCGGACAAAAAACAAC
Sm41	CCTCTCTATGGGCAGTCGGTGATCCAGCAGGTGCAGGCC
Sm93	CCTCTCTATGGGCAGTCGGTGATTGTGTGTCCGAGCTGCAC
Sm67	CCTCTCTATGGGCAGTCGGTGATCCGCCGACGAGGC
Sm68	CCTCTCTATGGGCAGTCGGTGATCCAACAACCCGACGGTTC
Bo13	CCTCTCTATGGGCAGTCGGTGATCGGTTCGCGGTGCCC
Bo32	CCTCTCTATGGGCAGTCGGTGATGCGTCTGCTCGCGAAAATGC
Bo35	CCTCTCTATGGGCAGTCGGTGATTGTCCCCCGAATGGTGGAC
Bo46	CCTCTCTATGGGCAGTCGGTGATATACCCGTACGCTGGCGC
Bo47	CCTCTCTATGGGCAGTCGGTGATCGGGTGGTGACGTCATCC
Bo48	CCTCTCTATGGGCAGTCGGTGATCGATGATGATTCAGCCGACGC
Bo53	CCTCTCTATGGGCAGTCGGTGATAAGACCAGCCCTACCGAAGC
Bo60	CCTCTCTATGGGCAGTCGGTGATCGCACACGCTTGCTTGAACATC
Bo71	CCTCTCTATGGGCAGTCGGTGATCGAGTGATCCCCGGCAC
Bo73	CCTCTCTATGGGCAGTCGGTGATGAAAAGTCAGCGGCCCTGAC
Bo78	CCTCTCTATGGGCAGTCGGTGATGCACCAAGACGCCGCATAC
Bo81	CCTCTCTATGGGCAGTCGGTGATGGGCGCGCTCTAGGC
Bo82	CCTCTCTATGGGCAGTCGGTGATGGTTCACCCGACCGCC
Bo86	CCTCTCTATGGGCAGTCGGTGATCCAACTCACCAGTTCCAGGTGATC
Bo87	CCTCTCTATGGGCAGTCGGTGATTCACACGCCCGCTGTCTTTC
Bo96	CCTCTCTATGGGCAGTCGGTGATCGCTTATCACGCGTTGTTGGC
Bo101	CCTCTCTATGGGCAGTCGGTGATCAGCACGCCGTCAGAGTTTC
Bo105	CCTCTCTATGGGCAGTCGGTGATGAGCCGATCAGCGACCACC
Bo118	CCTCTCTATGGGCAGTCGGTGATGCGTCCAGCTACCACCAC
Bo130	CCTCTCTATGGGCAGTCGGTGATTCTGGGTGAGCCGCGTTC
Bo132	CCTCTCTATGGGCAGTCGGTGATAAGGAAGACCTCGGCGTGTC
Bo27	CCTCTCTATGGGCAGTCGGTGATGCTAGCGTGACAGGCGTC
Bo29	CCTCTCTATGGGCAGTCGGTGATCTGCCGCGAAATCCGGC
Universal 5' primer	CCATCTCATCCCTGCGTGTCTCCGACTCAGCACTGCGTTTGCTGGCTTTGATG

Primers for 3’ Deep-RACE PCR

Sm11	CCTCTCTATGGGCAGTCGGTGATCGCTATCGCAACCCGTGC
Sm76	CCTCTCTATGGGCAGTCGGTGATGGAGTCCTGAGGGCTGC
Sm64	CCTCTCTATGGGCAGTCGGTGATGCAGCAGGCAGGCACTAC
Sm49	CCTCTCTATGGGCAGTCGGTGATGGCCACTAGGTGTAGTGTCTGA
Sm82	CCTCTCTATGGGCAGTCGGTGATCCTCGACATCAGGCTGATGC
Sm90	CCTCTCTATGGGCAGTCGGTGATGGGGACACCCCGCAAC
Sm38	CCTCTCTATGGGCAGTCGGTGATCGAGGCGATCGCGACC
Sm42	CCTCTCTATGGGCAGTCGGTGATCCACCTCGATCCCGCAC
Sm19	CCTCTCTATGGGCAGTCGGTGATGTTGTTTTTTGTCCGCCTCTTTTTCC
Sm41	CCTCTCTATGGGCAGTCGGTGATCCTGCACCTGCTGGGC
Sm93	CCTCTCTATGGGCAGTCGGTGATCGCGTCGGCACTGC
Sm67	CCTCTCTATGGGCAGTCGGTGATCGGAGACACACGGGGC
Sm68	CCTCTCTATGGGCAGTCGGTGATGCAGGTGGTAGGAACCGTC
Bo13	CCTCTCTATGGGCAGTCGGTGATCCACATGCTGGCTGTCGC
Bo32	CCTCTCTATGGGCAGTCGGTGATGCGTAGCGCCGCAC
Bo35	CCTCTCTATGGGCAGTCGGTGATCAACGAACCCGGAAATGTTGTCC
Bo46	CCTCTCTATGGGCAGTCGGTGATCCTAGGTCGACGGCCAC
Bo47	CCTCTCTATGGGCAGTCGGTGATGCAGCCATCAGCGGTCC
Bo48	CCTCTCTATGGGCAGTCGGTGATACCGGCGTCGGCTGAATC
Bo53	CCTCTCTATGGGCAGTCGGTGATAGGCGGCCATTGATGGC
Bo60	CCTCTCTATGGGCAGTCGGTGATGCTCCACCCGATGTTCAAGC
Bo71	CCTCTCTATGGGCAGTCGGTGATCGTCGCAACTCGCAGTGC
Bo73	CCTCTCTATGGGCAGTCGGTGATGCGCAGCTGCTCTGTCA
Bo78	CCTCTCTATGGGCAGTCGGTGATATGCGGCGTCTTGGTGC
Bo81	CCTCTCTATGGGCAGTCGGTGATGGGCCCTTAGAGCCTAGAGC
Bo82	CCTCTCTATGGGCAGTCGGTGATCCTGGGGGAGCGTGAATC
Bo86	CCTCTCTATGGGCAGTCGGTGATCGGGTGCACGCGATCAC
Bo87	CCTCTCTATGGGCAGTCGGTGATCCGGTAGGGTAGAGAAAGACAGC
Bo96	CCTCTCTATGGGCAGTCGGTGATGCCGTGGGCCAACAAC
Bo101	CCTCTCTATGGGCAGTCGGTGATCCCGTGAAACTCTGACGGC
Bo105	CCTCTCTATGGGCAGTCGGTGATTGACAGGGTGCGGTGGTC
Bo118	CCTCTCTATGGGCAGTCGGTGATCGGTGAAGGTGTCACCGC
Bo130	CCTCTCTATGGGCAGTCGGTGATGACGGGGCCAGCTCC
Bo132	CCTCTCTATGGGCAGTCGGTGATGGACCTCGGGCAGACAC
Bo27	CCTCTCTATGGGCAGTCGGTGATGGCGATCGGGTCCTAGC
Bo29	CCTCTCTATGGGCAGTCGGTGATGGATCCGTCGTGGTAGAAAGCC
Universal 3’ primer	CCATCTCATCCCTGCGTGTCTCCGACTCAGCATGCCGAGGTCGACTTCCTAG
