# Supplementary material for: Identification of Novel sRNAs in Mycobacterial Species
Source: PLoS One. 2013 Nov 14;8(11):e79411. doi: 10.1371/journal.pone.0079411 (PMC3828370; doi:10.1371/journal.pone.0079411)
Supplement: Table S3 — All 93 sRNA sequences predicted by SIPHT in M. smegmatis. (PDF) [file pone.0079411.s007.pdf]

Supplementary Table 3. *M. Smegmatis* MC155 8596 ALL SIPHT Predictions

sRNAName<br>~		sRNAstart	end	deep-sequenced in Mtb	dirsRNA	UpORFname	UpORFdir.	DnORFname	DnORFdir.
Candidate_1_NC_008596	Sm1	572813	572985		>>>	hypothetical protein	<<<	hypothetical protein	<<<
Candidate_2_NC_008596	Sm2	1077596	1077652		>>>	transport protein	<<<	hypothetical protein	>>>
Candidate_3_NC_008596	Sm3	1303781	1304147		>>>	ABC transporter substrate-bir	>>>	taurine import ATP-binding p	>>>
Candidate_4_NC_008596	Sm4	1460993	1461033		>>>	ABC transporter, ATP-binding	>>>	DNA-directed RNA polymera	>>>
Candidate_5_NC_008596	Sm5	1611415	1611475		>>>	hypothetical protein	<<<	translation initiation factor I	>>>
Candidate_6_NC_008596	Sm6	2016019	2016073	v	>>>	hypothetical protein	>>>	6-O-methylguanine DNA me	<<<
Candidate_7_NC_008596	Sm7	2047749	2047819		>>>	trRNA	>>>	hypothetical protein	<<<
Candidate_8_NC_008596	Sm8	2157557	2157607	v	>>>	alcohol dehydrogenase	<<<	putative acyl-CoA dehydroge	<<<
Candidate_9_NC_008596	Sm9	2236980	2237100		>>>	hypothetical protein	>>>	MmcJ protein	>>>
Candidate_10_NC_008596	Sm10	2370858	2370914		>>>	transport protein	<<<	hypothetical protein	>>>
Candidate_11_NC_008596	Sm11	2835902	2835989		>>>	TrkA protein	<<<	amino acid permease	>>>
Candidate_12_NC_008596	Sm12	2840315	2840380		>>>	diguanylate cyclase (ggdef) d	<<<	Na+/H+ antiporter NhaA	>>>
Candidate_13_NC_008596	Sm13	2937288	2937328		>>>	hypothetical protein	<<<	esterase	<<<
Candidate_14_NC_008596	Sm14	4208054	4208099		>>>	hydrolase, isochorismatase fa	>>>	TnpC protein	<<<
Candidate_15_NC_008596	Sm15	5519032	5519151		>>>	acyl-CoA synthase	<<<	4-diphosphocytidyl-2C-meth	<<<
Candidate_16_NC_008596	Sm16	6705492	6705532		>>>	hypothetical protein	<<<	TnpC protein	>>>
Candidate_17_NC_008596	Sm17	6709815	6709868		<<<	TnpC protein	>>>	hypothetical protein	<<<
Candidate_18_NC_008596	Sm18	5595798	5596221		<<<	hypothetical protein	>>>	von Willebrand factor, type A	<<<
Candidate_19_NC_008596	Sm19	5029530	5029661		<<<	trRNA	<<<	UDP-N-acetylglucosamine 1-	<<<
Candidate_20_NC_008596	Sm20	4761354	4761439		<<<	ATP-dependent Clp protease,	<<<	Clp protease	<<<
Candidate_21_NC_008596	Sm21	4663132	4663320		<<<	hypothetical protein	<<<	ribosomal protein S20	>>>
Candidate_22_NC_008596	Sm22	4625445	4625507		<<<	hypothetical protein	<<<	major membrane protein I	>>>
Candidate_23_NC_008596	Sm23	4154750	4154817		<<<	putative hydrolase	<<<	Transposase IS116/IS110/IS	>>>
Candidate_24_NC_008596	Sm24	4132172	4132623		<<<	haloacid dehalogenase, type I	>>>	translation initiation inhibito	>>>
Candidate_25_NC_008596	Sm25	3825197	3825307		<<<	trRNA	<<<	tyrosyl-tRNA synthetase	<<<
Candidate_26_NC_008596	Sm26	2887974	2888060		<<<	IS1549, transposase	>>>	hypothetical protein	<<<
Candidate_27_NC_008596	Sm27	2745574	2745649		<<<	hypothetical protein	<<<	dihydrodipicolinate reductas	>>>
Candidate_28_NC_008596	Sm28	2745536	2745649		<<<	hypothetical protein	<<<	dihydrodipicolinate reductas	>>>
Candidate_29_NC_008596	Sm29	1679794	1679850		<<<	TnpC protein	>>>	hypothetical protein	>>>
Candidate_30_NC_008596	Sm30	1403893	1403949		<<<	TnpC protein	>>>	oligopeptide transporter, OP	<<<
Candidate_31_NC_008596	Sm31	98574	98660		<<<	IS1549, transposase	>>>	hypothetical protein	>>>
Candidate_32_NC_008596	Sm32	417734	417792		<<<	3-ketoacyl-CoA thiolase	>>>	glycolate oxidase subunit	<<<
Candidate_33_NC_008596	Sm33	417709	417796		>>>	3-ketoacyl-CoA thiolase	>>>	glycolate oxidase subunit	<<<
Candidate_34_NC_008596	Sm34	996519	996570		>>>	isocitrate lyase	>>>	3-hydroxybutyryl-CoA dehyd	>>>
Candidate_35_NC_008596	Sm35	1458488	1458562		>>>	glucokinase	>>>	50S ribosomal protein L10	>>>
Candidate_36_NC_008596	Sm36	1532956	1533042		<<<	cytochrome P450-terp	<<<	cytochrome P450-terp	<<<
Candidate_37_NC_008596	Sm37	2031528	2031689		>>>	ATP-dependent DNA helicase	>>>	transcription factor WhiB	>>>
Candidate_38_NC_008596	Sm38	2236980	2237466		>>>	hypothetical protein	>>>	MmcJ protein	>>>
Candidate_39_NC_008596	Sm39	2428547	2428899		<<<	phytoene dehydrogenase	<<<	glycosyl hydrolase, family pr	<<<
Candidate_40_NC_008596	Sm40	3745897	3745992		>>>	hypothetical protein	>>>	phosphohydrolase	<<<
Candidate_41_NC_008596	Sm41	3815683	3815763		<<<	thiamin pyrophosphokinase, c	<<<	inorganic polyphosphate/ATP	<<<
Candidate_42_NC_008596	Sm42	4290257	4290618		>>>	putative transcriptional regula	>>>	universal stress protein fami	<<<
Candidate_43_NC_008596	Sm43	4392788	4392818	v	>>>	adenylate cyclase, putative	>>>	methyltransferase	<<<

Candidate_44_NC_008596	Sm44	5058951	5059019		<<<	trRNA	>>>	hypothetical protein	<<<
Candidate_45_NC_008596	Sm45	5736178	5736245		>>>	hypothetical protein	>>>	hypothetical protein	<<<
Candidate_46_NC_008596	Sm46	5864890	5864989	v	>>>	Glycine cleavage T-protein (a	>>>	hypothetical protein	>>>
Candidate_47_NC_008596	Sm47	6242319	6242668	v	>>>	morphological differentiation-	>>>	transcriptional regulator, Icl	<<<
Candidate_48_NC_008596	Sm48	6358104	6358152		>>>	D-amino-acid dehydrogenase	>>>	transcription elongation fact	>>>
Candidate_49_NC_008596	Sm49	1086797	1087035		>>>	secreted protein	>>>	glutaredoxin	>>>
Candidate_50_NC_008596	Sm50	1227456	1227772		>>>	hypothetical protein	>>>	hypothetical protein	<<<
Candidate_51_NC_008596	Sm51	1502615	1502749		<<<	translation elongation factor	>>>	hypothetical protein	>>>
Candidate_52_NC_008596	Sm52	1508311	1508345		>>>	hypothetical protein	>>>	arginine deiminase	>>>
Candidate_53_NC_008596	Sm53	1640871	1640914		<<<	propanediol utilization: dehyc	>>>	PduH protein	>>>
Candidate_54_NC_008596	Sm54	1733665	1733740	v	>>>	hypothetical protein	>>>	ABC transporter, ATP-bindin	<<<
Candidate_55_NC_008596	Sm55	1813059	1813310		<<<	IS3 family protein element, tı	>>>	ISMsm8, transposase	<<<
Candidate_56_NC_008596	Sm56	1895642	1896000		<<<	phosphoribosylaminoimidazol	>>>	acyl-CoA dehydrogenase	>>>
Candidate_57_NC_008596	Sm57	1923146	1923188		>>>	4Fe-4S ferredoxin, iron-sulfui	<<<	selenocysteine-specific trans	<<<
Candidate_58_NC_008596	Sm58	2000403	2000446		>>>	Transcription factor WhiB	<<<	diacylglycerol kinase, catalyi	<<<
Candidate_59_NC_008596	Sm59	2031644	2031683		<<<	ATP-dependent DNA helicase	>>>	transcription factor WhiB	>>>
Candidate_60_NC_008596	Sm60	2236287	2236418		>>>	hypothetical protein	>>>	MmcJ protein	>>>
Candidate_61_NC_008596	Sm61	2454079	2454123		>>>	Low molecular weight protein	<<<	acetolactate synthase, large	>>>
Candidate_62_NC_008596	Sm62	2482844	2482941	v	<<<	ribosomal protein L28	<<<	dihydroxyacetone kinase	>>>
Candidate_63_NC_008596	Sm63	2519697	2519753	v	<<<	hypothetical protein	>>>	30S ribosomal protein S16	>>>
Candidate_64_NC_008596	Sm64	2522821	2522986		<<<	LppW protein	<<<	ribosomal protein L19	>>>
Candidate_65_NC_008596	Sm65	2599592	2599668		>>>	hypothetical protein	>>>	tyrosine recombinase XerC	>>>
Candidate_66_NC_008596	Sm66	2600189	2600262		>>>	hypothetical protein	>>>	tyrosine recombinase XerC	>>>
Candidate_67_NC_008596	Sm67	2600389	2600484		>>>	hypothetical protein	>>>	tyrosine recombinase XerC	>>>
Candidate_68_NC_008596	Sm68	2600389	2600701		>>>	hypothetical protein	>>>	tyrosine recombinase XerC	>>>
Candidate_69_NC_008596	Sm69	2707720	2707775	v	<<<	hypothetical protein	>>>	hypothetical protein	<<<
Candidate_70_NC_008596	Sm70	2765999	2766035		>>>	hypothetical protein	<<<	acetyltransferase, gnat fami	<<<
Candidate_71_NC_008596	Sm71	2833294	2833408		<<<	hypothetical protein	<<<	OB-fold nucleic acid binding	>>>
Candidate_72_NC_008596	Sm72	2876967	2877054		>>>	sensor kinase	>>>	ABC efflux pump, fused inne	>>>
Candidate_73_NC_008596	Sm73	3022452	3022504		>>>	4-aminobutyrate transaminas	<<<	preprotein translocase, YajC	>>>
Candidate_74_NC_008596	Sm74	3111233	3111268		<<<	transcription antitermination	>>>	hypothetical protein	>>>
Candidate_75_NC_008596	Sm75	3124771	3124840		>>>	hypothetical protein	<<<	integration host factor	>>>
Candidate_76_NC_008596	Sm76	3690270	3690375		<<<	ComA operon protein 2	<<<	hypothetical protein	<<<
Candidate_77_NC_008596	Sm77	3755016	3755057		>>>	sodium:solute symporter	<<<	galactose-1-phosphate uridy	>>>
Candidate_78_NC_008596	Sm78	3811999	3812035		>>>	CTP synthase	<<<	thiamin pyrophosphokinase,	<<<
Candidate_79_NC_008596	Sm79	4215256	4215294		<<<	transposase	>>>	possible lysine decarboxylas	<<<
Candidate_80_NC_008596	Sm80	4376376	4376452		<<<	glutamine synthetase, type I	>>>	D-tyrosyl-tRNA(Tyr) deacyla	>>>
Candidate_81_NC_008596	Sm81	4376321	4376412		>>>	glutamine synthetase, type I	>>>	D-tyrosyl-tRNA(Tyr) deacyla	>>>
Candidate_82_NC_008596	Sm82	4392981	4393035	v	>>>	adenylate cyclase, putative	>>>	methyltransferase	<<<
Candidate_83_NC_008596	Sm83	5462387	5462435		<<<	hypothetical protein	<<<	dehydrogenase/reductase S	<<<
Candidate_84_NC_008596	Sm84	5519175	5519336		<<<	acyl-CoA synthase	<<<	4-diphosphocytidyl-2C-meth	<<<
Candidate_85_NC_008596	Sm85	5855903	5855951		>>>	phosphate ABC transporter, p	<<<	transcriptional regulatory pr	<<<
Candidate_86_NC_008596	Sm86	5963439	5963628		<<<	hypothetical protein	<<<	oxidoreductase, short chain	<<<
Candidate_87_NC_008596	Sm87	6078041	6078185		>>>	putative acyl-CoA dehydroger	<<<	putative acyl-CoA dehydroge	>>>
Candidate_88_NC_008596	Sm88	6152897	6152933		<<<	hypothetical protein	<<<	negative regulator of geneti	<<<
Candidate_89_NC_008596	Sm89	6707964	6708011		<<<	hypothetical protein	<<<	TnpC protein	>>>
Candidate_90_NC_008596	Sm90	6845964	6846035		>>>	carboxymuconolactone decarl	>>>	ribose operon repressor	<<<

Candidate_91_NC_008596	Sm91	6872580	6872809	<<<	NLP/P60 family protein	<<<	beta-lactamase	>>>
Candidate_92_NC_008596	Sm92	781990	782035	>>>	hypothetical protein	>>>	isoniazid inductible protein I	>>>
Candidate_93_NC_008596	Sm93	858466	858598	>>>	acetyltransferase	>>>	hypothetical protein	>>>

10 out of 93
