# Supplementary material for: Identification of Novel sRNAs in Mycobacterial Species
Source: PLoS One. 2013 Nov 14;8(11):e79411. doi: 10.1371/journal.pone.0079411 (PMC3828370; doi:10.1371/journal.pone.0079411)
Supplement: Table S4 — All 144 sRNA sequences predicted by SIPHT in M. bovis BCG. This list excludes predicted tRNAs. (PDF) [file pone.0079411.s008.pdf]

Supplementary Table 4. *M. bovis* BCG_008769 ALL SIPHT PREDICTIONS

sRNAName		sRNAstart	end	deep-sequenced in Mtb	dirsRNA	UpORFname	UpORFdir.	DnORFname	DnORFdir.
Candidate_1_NC_008769	Bo1	98023	98096	v	>>>	putative oxidoreductase	>>>	putative transmembrane pı	>>>
Candidate_2_NC_008769	Bo2	798782	798967	v	>>>	DNA-directed RNA polyn	>>>	putative hydrolase	<<<
Candidate_3_NC_008769	Bo3	1047536	1047598	v	>>>	putative transmembrane	>>>	putative dioxygenase	<<<
Candidate_4_NC_008769	Bo4	1047536	1047673	v	>>>	putative transmembrane	>>>	putative dioxygenase	<<<
Candidate_5_NC_008769	Bo5	1500061	1500136	v	>>>	trRNA	>>>	trRNA	>>>
Candidate_6_NC_008769	Bo6	1718898	1719041	v	>>>	hypothetical protein	>>>	hypothetical protein	<<<
Candidate_7_NC_008769	Bo7	1748024	1748149	v	>>>	putative transcriptional ı	>>>	hypothetical protein	>>>
Candidate_8_NC_008769	Bo8	2026073	2026203	v	>>>	putative ferredoxin	>>>	PPE family protein	>>>
Candidate_9_NC_008769	Bo9	2350888	2350947	v	>>>	hypothetical protein	<<<	hypothetical protein	<<<
Candidate_10_NC_008769	Bo10	2542128	2542214	v	>>>	hypothetical protein	<<<	putative aminotransferase	>>>
Candidate_11_NC_008769	Bo11	2716746	2716811	v	>>>	hypothetical protein	<<<	putative resuscitation-pron	<<<
Candidate_12_NC_008769	Bo12	2813325	2813408	v	>>>	putative fatty acid synth	<<<	hypothetical protein	<<<
Candidate_13_NC_008769	Bo13	3126934	3127070	v	>>>	putative glutamine synth	<<<	putative methionine amino	<<<
Candidate_14_NC_008769	Bo14	3375461	3375983	v	>>>	putative cytochrome P45	>>>	putative transcriptional reg	<<<
Candidate_15_NC_008769	Bo15	3505357	3505445	v	>>>	hypothetical protein	<<<	hypothetical protein	>>>
Candidate_16_NC_008769	Bo16	3777614	3777736	v	>>>	putative amidase amiD	>>>	hypothetical protein	>>>
Candidate_17_NC_008769	Bo17	3987546	3987591	v	>>>	hypothetical protein	<<<	putative oxidoreductase	<<<
Candidate_18_NC_008769	Bo18	4345842	4345991	v	>>>	hypothetical protein	<<<	hypothetical protein	<<<
Candidate_19_NC_008769	Bo19	3875949	3875999		<<<	PE family protein	>>>	hypothetical protein	>>>
Candidate_20_NC_008769	Bo20	3319114	3319148	v	<<<	hypothetical protein	<<<	putative lipoprotein lppZ	>>>
Candidate_21_NC_008769	Bo21	3236892	3236949	v	<<<	putative multifunctional	<<<	fatty-acid-CoA ligase fadD2	>>>
Candidate_22_NC_008769	Bo22	3131299	3131335	v	<<<	putative penicillin-bindin	<<<	hypothetical protein	>>>
Candidate_23_NC_008769	Bo23	2991016	2991111	v	<<<	putative acyl-CoA dehyd	<<<	putative GTP-binding prote	<<<
Candidate_24_NC_008769	Bo24	2985327	2985361	v	<<<	hypothetical protein	<<<	Repressor lexA	>>>
Candidate_25_NC_008769	Bo25	2247580	2247991		<<<	hypothetical protein	<<<	hypothetical protein	<<<
Candidate_26_NC_008769	Bo26	2200711	2200873	v	<<<	hypothetical protein	>>>	hypothetical protein	<<<
Candidate_27_NC_008769	Bo27	2157633	2157735	v	<<<	Ferric uptake regulation	<<<	hypothetical protein	<<<
Candidate_28_NC_008769	Bo28	2065765	2065809	v	<<<	putative drugs-transport	<<<	putative Acetolactate synth	>>>
Candidate_29_NC_008769	Bo29	1769806	1770012	v	<<<	putative acyltransferase	>>>	putative fumarate reductas	>>>
Candidate_30_NC_008769	Bo30	1234453	1234611	v	<<<	putative cystathionine b	>>>	putative Proline-rich antige	>>>
Candidate_31_NC_008769	Bo31	1013063	1013213	v	<<<	putative phosphoserine a	<<<	hypothetical protein	>>>
Candidate_32_NC_008769	Bo32	817449	817557	v	<<<	putative elongation facto	>>>	hypothetical protein	>>>
Candidate_33_NC_008769	Bo33	621733	621908		<<<	putative pyrroline-5-carl	>>>	hypothetical protein	>>>
Candidate_34_NC_008769	Bo34	589648	589705	v	<<<	Isocitrate lyase icl	>>>	putative 3-hydroxybutyryl-	>>>
Candidate_35_NC_008769	Bo35	576077	576189	v	<<<	hypothetical protein	>>>	hypothetical protein	<<<
Candidate_36_NC_008769	Bo36	358379	358411	v	<<<	putative transcriptional ı	<<<	hypothetical protein	>>>
Candidate_37_NC_008769	Bo37	305783	305928	v	<<<	putative acyl-CoA dehyd	>>>	putative transcriptional reg	>>>
Candidate_38_NC_008769	Bo38	129001	129398	v	<<<	hypothetical protein	>>>	putative bifunctional mta/s	>>>
Candidate_39_NC_008769	Bo39	186345	186468	v	>>>	putative transmembrane	>>>	Secreted antigen 85-c fbpC	<<<
Candidate_40_NC_008769	Bo40	798737	798796	v	<<<	DNA-directed RNA polyn	>>>	putative hydrolase	<<<
Candidate_41_NC_008769	Bo41	935527	935628	v	<<<	hypothetical protein	<<<	hypothetical protein	<<<
Candidate_42_NC_008769	Bo42	2439908	2440111	v	<<<	putative Ubiquinol-cytoc	>>>	putative transmembrane pı	<<<

Candidate_43_NC_008769	Bo43	2439965	2440111	v	<<<	putative Ubiquinol-cytoc	>>>	putative transmembrane p	<<<
Candidate_44_NC_008769	Bo44	2440022	2440111	v	<<<	putative Ubiquinol-cytoc	>>>	putative transmembrane p	<<<
Candidate_45_NC_008769	Bo45	2531945	2532009	v	>>>	putative phosphate-tran	>>>	putative transcription regul	<<<
Candidate_46_NC_008769	Bo46	2602899	2602984	v	<<<	putative transmembrane	>>>	putative ESAT-6 like protei	<<<
Candidate_47_NC_008769	Bo47	2705736	2705878	v	<<<	putative 50S ribosomal	<<<	putative C4-dicarboxylate-	>>>
Candidate_48_NC_008769	Bo48	3028829	3029036	v	<<<	putative alanine rich hyc	>>>	putative short-chain type d	<<<
Candidate_49_NC_008769	Bo49	3089636	3089773	v	<<<	hypothetical protein	<<<	hypothetical protein	<<<
Candidate_50_NC_008769	Bo50	3512558	3512644	v	>>>	hypothetical alanine and	>>>	putative transmembrane p	<<<
Candidate_51_NC_008769	Bo51	3560769	3560845	v	<<<	putative oxidoreductase	<<<	hypothetical protein	<<<
Candidate_52_NC_008769	Bo52	4073793	4074096	v	>>>	hypothetical protein	>>>	hypothetical protein	<<<
Candidate_53_NC_008769	Bo53	1044604	1044769	v	>>>	putative metal cation tra	>>>	hypothetical protein	>>>
Candidate_54_NC_008769	Bo54	1050442	1050536	v	>>>	putative lipid carrier pro	<<<	PPE family protein	<<<
Candidate_55_NC_008769	Bo55	1088544	1088593	v	<<<	hypothetical protein	<<<	putative ATP dependent dn	>>>
Candidate_57_NC_008769	Bo57	1158391	1158486	v	>>>	putative deoxyribonucle	>>>	putative resuscitation-pron	>>>
Candidate_58_NC_008769	Bo58	1205651	1205934	v	<<<	hypothetical protein	<<<	hypothetical protein	>>>
Candidate_59_NC_008769	Bo59	1240958	1241026	v	>>>	hypothetical protein	>>>	putative hemolysin-like pro	<<<
Candidate_60_NC_008769	Bo60	1247488	1247725	v	<<<	putative cellulase celA2b	>>>	PE-PGRS family protein	>>>
Candidate_61_NC_008769	Bo61	1303330	1303446	v	<<<	putative short-chain typ	>>>	putative transmembrane tr	>>>
Candidate_62_NC_008769	Bo62	1309167	1309308	v	<<<	hypothetical protein	<<<	putative transposase	>>>
Candidate_63_NC_008769	Bo63	1344627	1344782	v	<<<	hypothetical protein	<<<	putative polyketide beta-ke	>>>
Candidate_64_NC_008769	Bo64	1344598	1344666	v	>>>	hypothetical protein	<<<	putative polyketide beta-ke	>>>
Candidate_65_NC_008769	Bo65	1393139	1393208	v	>>>	putative transcriptional	<<<	putative methyltransferase	<<<
Candidate_66_NC_008769	Bo66	1393968	1394033	v	>>>	putative methyltransfera	<<<	Alternative RNA polymeras	>>>
Candidate_67_NC_008769	Bo67	1394909	1395111	v	>>>	Alternative RNA polymer	>>>	hypothetical protein	>>>
Candidate_68_NC_008769	Bo68	1498195	1498308	v	>>>	putative UDP-N-acetylgl	>>>	trRNA	>>>
Candidate_69_NC_008769	Bo69	1499908	1499978	v	<<<	trRNA	>>>	trRNA	>>>
Candidate_70_NC_008769	Bo70	1503529	1503647	v	<<<	trRNA	>>>	putative methylated-DNA--	<<<
Candidate_71_NC_008769	Bo71	1588693	1588853	v	<<<	PPE family protein	>>>	putative integration host fa	>>>
Candidate_72_NC_008769	Bo72	1589405	1589534	v	<<<	putative integration host	>>>	putative guanylate kinase	>>>
Candidate_73_NC_008769	Bo73	1647817	1647853	v	>>>	hypothetical protein	<<<	hypothetical protein	<<<
Candidate_74_NC_008769	Bo74	1717786	1718027	v	>>>	hypothetical protein	>>>	hypothetical protein	>>>
Candidate_75_NC_008769	Bo75	1857323	1857431	v	>>>	putative lysyl-tRNA synt	<<<	putative INITIATION FACT	>>>
Candidate_76_NC_008769	Bo76	1952894	1952974	v	>>>	hypothetical protein	<<<	putative carboxylase	>>>
Candidate_77_NC_008769	Bo77	2025595	2025832	v	<<<	putative cytochrome P4	<<<	putative ferredoxin	>>>
Candidate_78_NC_008769	Bo78	207040	207399	v	<<<	hypothetical protein	<<<	PE family protein	<<<
Candidate_79_NC_008769	Bo79	210820	210981	v	>>>	PE family protein	<<<	Phosphotyrosine protein ph	<<<
Candidate_80_NC_008769	Bo80	2109967	2110040	v	<<<	alanine and proline rich	>>>	putative transmembrane p	>>>
Candidate_81_NC_008769	Bo81	218738	218805	v	>>>	PE family protein	<<<	PE family protein	<<<
Candidate_82_NC_008769	Bo82	2235199	2235271	v	<<<	putative ferredoxin fdxA	<<<	hypothetical protein	<<<
Candidate_83_NC_008769	Bo83	2241726	2242050	v	>>>	hypothetical protein	<<<	hypothetical protein	>>>
Candidate_84_NC_008769	Bo84	2290721	2290870	v	<<<	putative polyketide synt	<<<	hypothetical protein	<<<
Candidate_85_NC_008769	Bo85	2300720	2300817	v	<<<	hypothetical protein	<<<	putative cobalamin biosynt	<<<
Candidate_86_NC_008769	Bo86	2325795	2325960	v	>>>	hypothetical protein	>>>	putative transmembrane se	>>>
Candidate_87_NC_008769	Bo87	2350914	2350995	v	<<<	hypothetical protein	<<<	hypothetical protein	<<<
Candidate_88_NC_008769	Bo88	2389602	2389705	v	>>>	cell division protein FtsZ	<<<	putative cell division protei	<<<
Candidate_89_NC_008769	Bo89	2404835	2404935	v	<<<	PE-PGRS family protein	<<<	putative penicillin-binding	<<<
Candidate_90_NC_008769	Bo90	2417687	2417812	v	<<<	putative transmembrane	>>>	putative transposase	<<<

Candidate_91_NC_008769	Bo91	2443488	2443536	v	<<<	putative transmembrane	<<<	putative asparagine synthe	>>>
Candidate_92_NC_008769	Bo92	2454616	2454699	v	>>>	putative branched-chain	<<<	putative aminomethyltrans	<<<
Candidate_93_NC_008769	Bo93	2519844	2519973	v	>>>	putative cytochrome P45	>>>	hypothetical protein	<<<
Candidate_95_NC_008769	Bo95	2685842	2685967	v	<<<	hypothetical protein	>>>	putative transposase	<<<
Candidate_96_NC_008769	Bo96	2686969	2687035	v	>>>	putative transposase	<<<	hypothetical protein	<<<
Candidate_97_NC_008769	Bo97	2704858	2704902	v	>>>	putative GTP1/obg-famil	<<<	putative 50S ribosomal pro	<<<
Candidate_98_NC_008769	Bo98	2757824	2757868	v	>>>	putative carboxylesteras	<<<	trRNA	>>>
Candidate_99_NC_008769	Bo99	2845488	2845859	v	<<<	putative proline and glyc	>>>	hypothetical protein	>>>
Candidate_100_NC_008769	Bo100	2846599	2846750	v	>>>	hypothetical protein	>>>	putative glutamine-transpo	>>>
Candidate_101_NC_008769	Bo101	2919147	2919502	v	<<<	hypothetical protein	<<<	hypothetical protein	>>>
Candidate_102_NC_008769	Bo102	2949474	2949742	v	<<<	putative enoyl-CoA hydr	>>>	hypothetical protein	>>>
Candidate_103_NC_008769	Bo103	2977134	2977209	v	>>>	RNA polymerase sigma f	>>>	Iron-dependent repressor a	>>>
Candidate_104_NC_008769	Bo104	3058401	3058476	v	>>>	hypothetical protein	<<<	putative lipoprotein lppV	<<<
Candidate_105_NC_008769	Bo105	3073445	3073541	v	>>>	hypothetical protein	>>>	putative transposase	<<<
Candidate_106_NC_008769	Bo106	3076849	3077266	v	<<<	putative transposase	<<<	hypothetical protein	<<<
Candidate_107_NC_008769	Bo107	3077361	3077492	v	<<<	putative transposase	<<<	hypothetical protein	<<<
Candidate_108_NC_008769	Bo108	3087488	3087694	v	<<<	hypothetical protein	<<<	hypothetical protein	<<<
Candidate_109_NC_008769	Bo109	315103	315226		>>>	Small secreted protein	<<<	putative lipoprotein lpqI	>>>
Candidate_110_NC_008769	Bo110	3171597	3171645	v	>>>	putative 30S ribosomal p	<<<	hypothetical protein	<<<
Candidate_111_NC_008769	Bo111	321585	321741	v	>>>	putative acetyl-CoA acyl	>>>	putative acyl-CoA dehydro	<<<
Candidate_112_NC_008769	Bo112	3261506	3261740	v	<<<	hypothetical protein	<<<	hypothetical protein	<<<
Candidate_113_NC_008769	Bo113	3317520	3317587	v	<<<	putative acetolactate syn	<<<	Low molecular weight prote	>>>
Candidate_114_NC_008769	Bo114	3341355	3341487	v	>>>	hypothetical protein	<<<	putative electron transfer f	<<<
Candidate_115_NC_008769	Bo115	3506359	3506470	v	<<<	putative transcriptional r	>>>	hypothetical protein	>>>
Candidate_116_NC_008769	Bo116	3602556	3602704	v	<<<	hypothetical protein	>>>	hypothetical protein	>>>
Candidate_117_NC_008769	Bo117	3731003	3731279	v	<<<	hypothetical protein	<<<	pseudogene	<<<
Candidate_118_NC_008769	Bo118	3765894	3765980	v	<<<	putative tRNA/rRNA met	>>>	PE-PGRS family protein	>>>
Candidate_119_NC_008769	Bo119	3827924	3828035	v	>>>	putative alanine racema	<<<	hypothetical protein	<<<
Candidate_120_NC_008769	Bo120	3919308	3919530	v	>>>	hypothetical protein	<<<	PE-PGRS family protein	>>>
Candidate_121_NC_008769	Bo121	3944210	3944265	v	>>>	hypothetical protein	<<<	hypothetical protein	<<<
Candidate_122_NC_008769	Bo122	4019787	4019854	v	<<<	putative ATP-dependent	<<<	putative lsr2 protein precu	<<<
Candidate_123_NC_008769	Bo123	4042104	4042175	v	>>>	putative integral membr	<<<	putative integral membran	>>>
Candidate_124_NC_008769	Bo124	4061216	4061329	v	>>>	hypothetical protein	<<<	putative cold shock protein	<<<
Candidate_125_NC_008769	Bo125	4066488	4066544	v	<<<	hypothetical protein	>>>	PE-PGRS family protein	>>>
Candidate_126_NC_008769	Bo126	4072493	4072633	v	<<<	hypothetical protein	<<<	hypothetical protein	>>>
Candidate_127_NC_008769	Bo127	4083543	4083676	v	<<<	putative protease	<<<	putative transmembrane p	>>>
Candidate_128_NC_008769	Bo128	4099732	4100035	v	>>>	trRNA	>>>	putative cytochrome P450	<<<
Candidate_129_NC_008769	Bo129	4130474	4130544	v	<<<	putative dna polymerase	<<<	putative ligase	>>>
Candidate_130_NC_008769	Bo130	413288	413416	v	<<<	hypothetical proline and	>>>	hypothetical protein	>>>
Candidate_131_NC_008769	Bo131	4142003	4142145	v	>>>	trRNA	>>>	putative transmembrane p	>>>
Candidate_132_NC_008769	Bo132	4260533	4260570	v	>>>	hypothetical protein	>>>	putative integral membran	<<<
Candidate_133_NC_008769	Bo133	4350049	4350265	v	<<<	hypothetical protein	<<<	hypothetical protein	<<<
Candidate_134_NC_008769	Bo134	505658	505708	v	>>>	putative secreted protei	<<<	hypothetical protein	>>>
Candidate_136_NC_008769	Bo136	610116	610263	v	>>>	putative phosphoglycera	>>>	putative two component se	>>>
Candidate_137_NC_008769	Bo137	779373	779435	v	>>>	putative sugar kinase	>>>	putative 50S ribosomal pro	>>>
Candidate_138_NC_008769	Bo138	817494	817557	v	<<<	putative elongation facto	>>>	hypothetical protein	>>>
Candidate_139_NC_008769	Bo139	818357	818428	v	>>>	hypothetical protein	>>>	putative short-chain type d	>>>

Candidate_140_NC_008769	Bo140	837644	837759	v	<<<	putative 50S ribosomal		>>>	putative arylsulfatase atsA	>>>
Candidate_141_NC_008769	Bo141	908489	908530	v	<<<	putative protease II ptrE	>>>	putative multidrug resistan	<<<
Candidate_142_NC_008769	Bo142	908364	908555	v	>>>	putative protease II ptrE	>>>	putative multidrug resistan	<<<
Candidate_143_NC_008769	Bo143	936775	936827	v	<<<	hypothetical protein	<<<	putative amino acid aminot	>>>
Candidate_144_NC_008769	Bo144	954667	954869	v	<<<	trRNA	>>>	PE-PGRS family protein	>>>
