# Supplementary material for: Identification of Novel sRNAs in Mycobacterial Species
Source: PLoS One. 2013 Nov 14;8(11):e79411. doi: 10.1371/journal.pone.0079411 (PMC3828370; doi:10.1371/journal.pone.0079411)
Supplement: Table S5 — All transcription factor ChIP-seq peaks located within 100 bp upstream and 20 bp downstream of sRNA 5′ ends. (PDF) [file pone.0079411.s009.pdf]

Supplementary Table 5. Transcription factor ChIP-seq peaks located within 100 bp upstream and 20 bp downstream of sRNA 5' ends.

sRNA	New nomenclature	Transcription Factor	5' end - peak distance
B55[a]	ncRv10609A	Rv3249c	-29[e]
B11[a]	ncRv13660Ac	Rv0081[d]	-56
B11[a]	ncRv13660Ac	Rv0081[d]	-45
B11[a]	ncRv13660Ac	Rv3249c	-25
B11[a]	ncRv13660Ac	Rv2034	-20.5
Mpr5[b]	ncBCG11109A/ncRv11051A	Rv0324	-27
Mcr11[b]	ncBCG11323Ac/ncRv11264A	Rv3574	-52
Mcr8/Mpr4[b]	ncBCG13719A/ncRv13661A	Rv0821c (PhoY2)	-38[e]
Bo29[c]	ncBCG11603Ac	Rv2021c	-19
Bo132[c]	ncBCG13885A	Rv2887	-44[e]

[a] Arnvig and Young, 2009
[b] DiChiara *et al.*, 2010
[c] this work
[d] replicate ChIP-seq datasets are available for Rv0081 and we required that sRNA-proximal ChIP-seq peaks be present in both replicates for inclusion in this table
[e] unambiguously associated with an sRNA 5' end
